# Clinical Significance of *SERPINA1* Gene and Its Encoded Alpha1-Antitrypsin Protein in NSCLC

**DOI:** 10.3390/cancers11091306

**Published:** 2019-09-04

**Authors:** Evrim Ercetin, Sarah Richtmann, Beatriz Martinez Delgado, Gema Gomez-Mariano, Sabine Wrenger, Elena Korenbaum, Bin Liu, David DeLuca, Mark P. Kühnel, Danny Jonigk, Kadriya Yuskaeva, Arne Warth, Thomas Muley, Hauke Winter, Michael Meister, Tobias Welte, Sabina Janciauskiene, Marc A. Schneider

**Affiliations:** 1 Department of Respiratory Medicine, Hannover Medical School, 30625 Hannover, Germany; 2 Biomedical Research in Endstage and Obstructive Lung Disease Hannover (BREATH), Member of the German Center for Lung Research (DZL), 30625 Hannover, Germany; 3 Translational Research Unit, Thoraxklinik at Heidelberg University Hospital, 69126 Heidelberg, Germany; 4 Translational Research Center Heidelberg (TLRC), Member of the German Center for Lung Research (DZL), 69120 Heidelberg, Germany; 5 Department of Molecular Genetics, Institute of Health Carlos III, Center for Biomedical Research in the Network of Rare Diseases (CIBERER), 28220 Majadahonda (Madrid), Spain; 6 Institute of Biophysical Chemistry and Anatomy, Hannover Medical School, 30625 Hannover, Germany; 7 Institute of Pathology, Hannover Medical School, 30625 Hannover, Germany; 8 Institute of Pathology, Heidelberg University Hospital, D-69120 Heidelberg, Germany; 9 Department of Surgery, Thoraxklinik at Heidelberg University Hospital, D-69126 Heidelberg, Germany

**Keywords:** alpha1-antitrypsin, *SERPINA1*, lung cancer, inflammation, acute phase proteins, apoptosis, migration

## Abstract

High expression of *SERPINA1* gene encoding acute phase protein, alpha1-antitrypsin (AAT), is associated with various tumors. We sought to examine the significance of *SERPINA1* and AAT protein in non-small-cell lung cancer (NSCLC) patients and NSCLC cell lines. Tumor and adjacent non-tumor lung tissues and serum samples from 351 NSCLC patients were analyzed for *SERPINA1* expression and AAT protein levels. We also studied the impact of *SERPINA1* expression and AAT protein on H1975 and H661 cell behavior, *in vitro*. Lower *SERPINA1* expression in tumor but higher in adjacent non-tumor lung tissues (*n* = 351, *p* = 0.016) as well as higher serum levels of AAT protein (*n* = 170, *p* = 0.033) were associated with worse survival rates. Specifically, in NSCLC stage III patients, higher blood AAT levels (>2.66 mg/mL) correlated with a poor survival (*p* = 0.002). Intriguingly, levels of serum AAT do not correlate with levels of C-reactive protein, neutrophils-to-leukocyte ratio, and do not correlate with *SERPINA1* expression or AAT staining in the tumor tissue. Additional experiments *in vitro* revealed that external AAT and/or overexpressed *SERPINA1* gene significantly improve cancer cell migration, colony formation and resistance to apoptosis. *SERPINA1* gene and AAT protein play an active role in the pathogenesis of lung cancer and not just reflect inflammatory reaction related to cancer development.

## 1. Introduction

Lung cancer is divided in non-small cell lung cancer (NSCLC, 85% of all lung cancers) and small cell lung cancer (SCLC, 15% of all lung cancers). NSCLC can be further classified into the main histological groups such as large-cell neuroendocrine carcinoma (LCNEC), squamous cell carcinoma (SQCC), and adenocarcinoma (ADC) [1]. Lung cancer is a leading cause of cancer-related death worldwide [2] because the majority of patients are diagnosed at a late-stage of the disease, making successful treatment more difficult and survival outcomes poor [3]. Moreover, invasiveness and metastasis are major threats to successful treatment of lung cancer. In the metastatic process of lung carcinomas, many mechanisms and involved genes/proteins have been identified, but the breakthrough is still not achieved. The treatments for lung cancer have improved recent years, and therefore the identification of prognostic factors is of great importance because they can contribute to clinical decision making and help to individualize treatments for this heterogeneous patient population.

A systemic inflammatory response is more and more being recognized as a prognostic factor in cancer patients. The hallmarks of cancer-related inflammation include inflammatory cells and inflammatory mediators, tissue remodeling and angiogenesis factors, similar like those seen in chronic inflammatory conditions or in tissue repair [4]. For example, several studies have shown that neutrophil-to-lymphocyte ratio has a prognostic value in predicting the survival of patients with NSCLC [5,6]. The increased levels of circulating acute phase proteins are also considered as prognostic factors for a poor survival in various cancers. For instance, C-reactive protein (CRP) is negatively associated with lung cancer outcome. The Rotterdam Study [7] demonstrated that high levels (>3 mg/L) of CRP are associated with an increased incidence of lung cancer. Moreover, elevated CRP levels are considered as an independent and significant prognostic indicator in patients with NSCLC [8,9,10]. Other acute phase proteins, including haptoglobin, alpha-2 macroglobulin and alpha1-antitrypsin (AAT) are also suggested as suitable targets for therapeutic and biomarker discoveries in cancer [11,12].

Alpha-1-antitrypsin (AAT), encoded by *SERPINA1* gene, is an acute phase glycoprotein mainly (by 80%) synthesized in human liver and is a major blood protein after albumin and the immunoglobulins [13]. The promoter of the *SERPINA1* is responsive to the IL-6 and IL-1 pathways, and to hypoxia [14]. The AAT is an archetype member of SERPIN (serine protease inhibitor) super-family and best characterized as a controller of lung tissue damage through its inhibitory effect on neutrophil serine proteases [15]. Recent findings support AAT’s broader role in modulating acute inflammatory processes via protease inhibitory and non-inhibitory mechanisms. In general, the biological role of AAT seems to be one of maintaining homeostasis and improving tissue repair and regeneration. It is proposed that during chronic inflammation, which is a driving force in cancer development, increased levels and functional activity of AAT may favor cancer progression. Several studies demonstrated that higher levels of AAT correlate with more advanced cancer stages [16]. A high level of AAT in breast cancer patients has been associated with poor clinical prognosis [17]. Elevated serum levels of AAT have been reported in patients with lung cancer as compared to those without lung cancer [18,19]. Some studies have shown that patients with *SERPINA1* expression in their tumor cells have worse prognosis than those without expression [20]. So far, the associations between clinical prognosis of NSCLC patients and *SERPINA1* expression and AAT levels in tumor and adjacent non-tumor tissues as well as serum AAT concentrations have not been reported. It is also of interest to clarify whether *SERPINA1* gene and AAT protein play an active role in the pathogenesis of lung cancer or just reflect inflammatory reaction related to cancer development.

## 2. Results

### 2.1. SERPINA1 Gene Expression Is Lower in NSCLC Tumors as Compared to the Normal Adjacent Lung Tissue and Prognostic for Patients’ Survival

**Figure 1:**
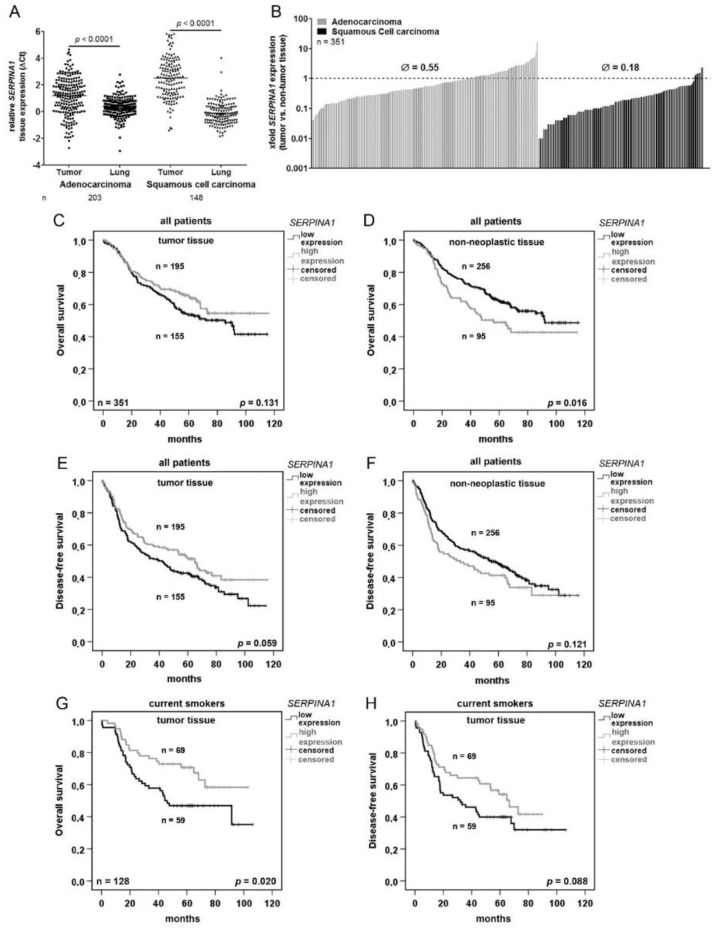
*SERPINA1* expression is downregulated in NSCLC and is a prognostic factor for overall and disease-free survival. (**A**) Relative expression (ΔCt) of *SERPINA1* in tumor and in paired non-tumor lung tissues. *SERPINA1* expression was normalized to reference genes *ESD* and *RPS18*. A higher relative value means a lower gene expression. (**B**) *SERPINA1* expression ratio (tumor vs. non-tumor lung tissue) in 351 NSCLC patients. Dotted line indicates equal expression in tumor and lung tissue. (**C**–**F**). Kaplan-Meier overall and disease-free survival curves for all patients. (**G**) and (**H**) Kaplan-Meier overall and disease-free survival curves for current smokers. The program cut-off finder (http://molpath.charite.de/cutoff/) was used to define the values separating the groups. *p* < 0.05 was considered significant.

To get an insight of *SERPINA1* expression in NSLC, in a first step, *SERPINA1* gene expression was analyzed in a cohort of tumor and adjacent non-tumor tissues of the lung obtained from 351 patients (Table 1). In general, *SERPINA1* expression was significantly lower in tumor tissues than in adjacent normal lung tissue. A high variability in expression levels was detected in both, ADC and SQCC cases but not in the non-neoplastic tissues (Figure 1A). Further statistical analyses revealed that *SERPINA1* expression is by 0.55-fold lower in ADC and by 0.18-fold lower in SQCC if compared to the corresponding non-neoplastic tissue (Figure 1B). 

For survival analyses, we used *SERPINA1* expression cut-offs based on a software tool “Cutoff-Finder” (see the Material and Methods section and Supplementary Figure S1A–D). Based on the Cox regression univariate analysis we found that higher *SERPINA1* expression in adjacent non-tumor tissue is related to worse overall survival (Table 2). As illustrated in Figure 1C,D, the better overall survival of the patients was related to higher *SERPINA1* expression in the tumor but lower expression in non-neoplastic tissue. The same trend was seen regarding disease-free survival (Figure 1E,F). Specifically, in current smokers a higher tumor expression of *SERPINA1* was significantly related to the better overall survival as well as disease-free survival (Figure 1G,H). Survival prognoses of ex-smokers and non-smokers were not related to *SERPINA1* expression levels (*p* = 0.520 and *p* = 0.592, data not shown). The Kaplan-Meier curves based on the ratio between relative *SERPINA1* expression in tumor and in paired lung tissue show that overall and disease-free patient’s survival is better when tumor vs non-tumor ratio for *SERPINA1* expression is >0.26-fold (Supplementary Figure S1E,F).

To check whether our results in NSCLS patient cohort align with data from NSCLC-TCGA we performed survival analyses using TCGA data [21]. Using a similar expression cut-off for an equal group separation, we found no significant correlation between tumor *SERPINA1* expression and overall patient survival (Supplementary Figure S1G).

### 2.2. NSCLC Tumor Cells Are Heterogeneously Stained for AAT and Positive Tumor Cells May Serve as A Prognostic Marker

**Figure 2:**
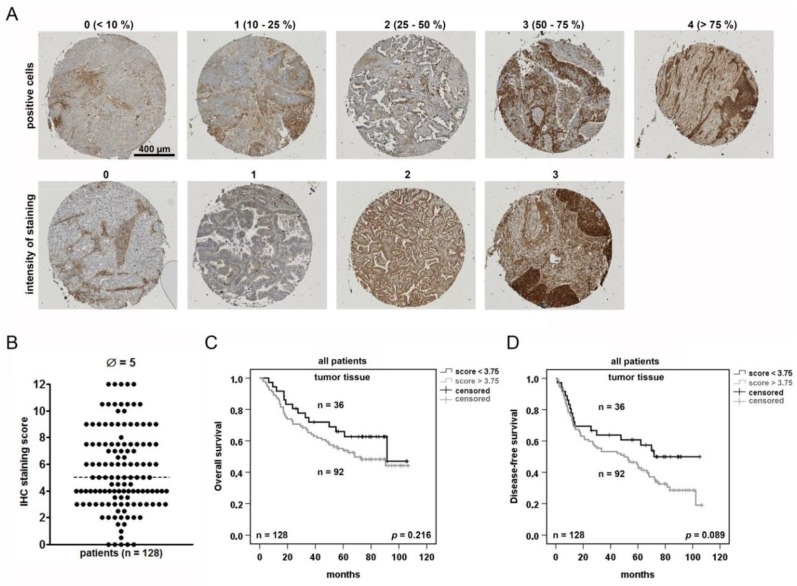
Tumor cells positive for AAT are prognostic indicator for the patient’s disease-free survival. (**A**) Scoring of NSCLC according to a positive AAT staining. Tissue micro array (TMA) with two cores for each sample was stained for AAT. (**B**) Immunohistochemistry (IHC) score generated from multiplication of intensity of staining and positivity for AAT protein. (**C**) and (**D**) Kaplan-Meier overall and disease-free survival curves using the staining score of 3.75 as cut-off to separate the two groups. *p* < 0.05 was considered significant.

Tumor tissue contains not only tumor cells, but also numbers of other cells, including tumor-associated macrophages, lymphocytes, blood vessels, and fibroblasts that affect tumor development in various ways. Therefore, we performed tumor tissue immunostainings to check for AAT-positive tumor cells and their putative prognostic relevance. The tissue micro array (TMA) cores were generated from a part (*n* = 128) of the NSCLC patient cohort (Table 1), stained for AAT and analyzed with an IHC score (Figure 2A). The antibody dilutions and controls guaranteed an optimal staining (Supplementary Figure S2A–C). For the score calculation, the proportion of positive tumor cells (0–4) was multiplied with the staining intensity (0–3). A median IHC score was 5 with a wide range of staining intensities in the tumor but also in the non-tumor areas (Supplementary Figure S2D). Based on the software tool “Cutoff-Finder”, the cut-off was selected for statistical analyses (Supplementary Figure S2E,F). The Cox regression analyses (Table 2) and the Kaplan-Meier plots (Figure 2C) revealed that AAT IHC score has no significant influence on the patient survival. Nevertheless, by using the cut-off value of a staining score of 3.75, we found a trend between tumor AAT IHC score < 3.75 and a better disease-free survival (Figure 2D).

### 2.3. Serum AAT Concentration does not Correlate With SERPINA1 Expression and AAT IHC Staining but Is Prognostic Depending on the Disease Stage 

**Figure 3:**
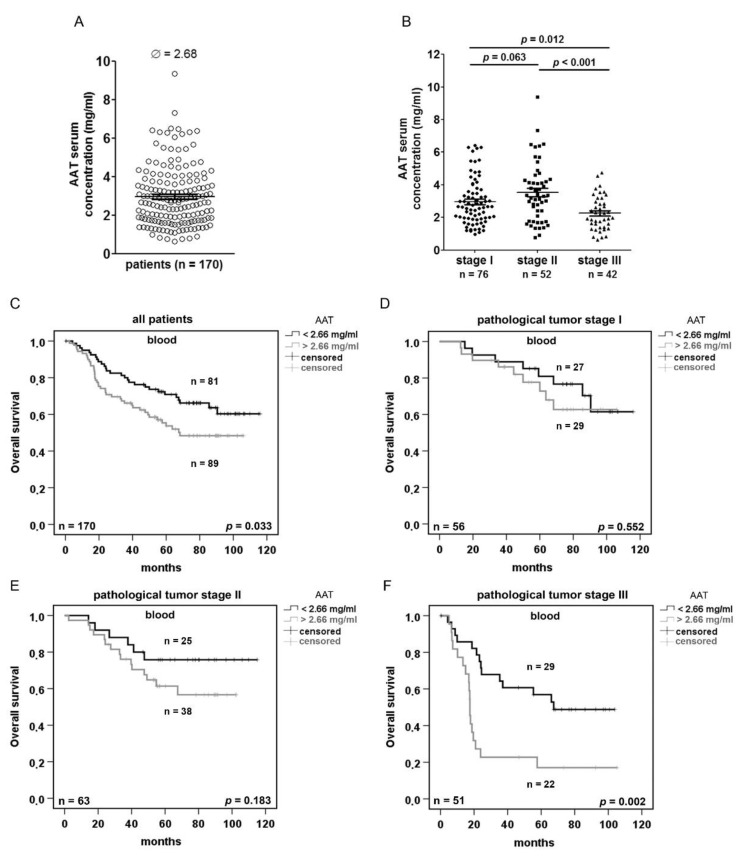
Serum levels of AAT in NSCLC patients increase with advanced tumor stages and are prognostic for the patient’s outcome. (**A**) ELISA based quantification of serum AAT in 170 NSCLC patients. (**B**) Serum concentrations of AAT in patients with stage cancer I, II and III. (**C–F**) Kaplan-Meier overall survival curves using 2.66 mg/mL AAT as cut-off to separate the two groups. *p* < 0.05 was considered significant.

We next were interested in the serum levels of AAT at the diagnosis and their prognostic relevance for the NSCLC patients. We measured AAT concentrations in approx. the half of the NSCLC patient cohort (*n* = 170). As illustrated in Figure 3A, a median serum concentration of AAT was 2.68 mg/mL. However, we found no correlation between serum concentrations of AAT, tumor or non-tumor tissue expression of *SERPINA1* gene and the IHC score, respectively (Supplementary Figure S3A–C). In general, however, serum levels of AAT were significantly lower in patients diagnosed with stage III as compared to patients with stage I and II lung cancer (Figure 3B).

Based on the software tool “Cutoff-Finder”, we introduced the cut-off of AAT at 2.66 mg/mL (Supplementary Figure S3D). Indeed, serum concentrations of AAT < 2.66 mg/mL were associated with a better overall patient survival than compared to those with higher AAT concentrations (Figure 3C). The comparison between pathological tumor stages and patient overall and disease-free survival based on the cut-off of AAT, clearly show that lower serum levels of AAT associate with better survival, especially in patients with tumor stage III (Figure 3D–F and Supplementary Figure S3E–G). 

### 2.4. Serum Concentration of C-reactive Protein (CRP) and Neutrophils/Lymphocytes Ratio (NLR) Increase in Advanced Tumor Stage but do not Correlated with AAT Serum Concentration

**Figure 4:**
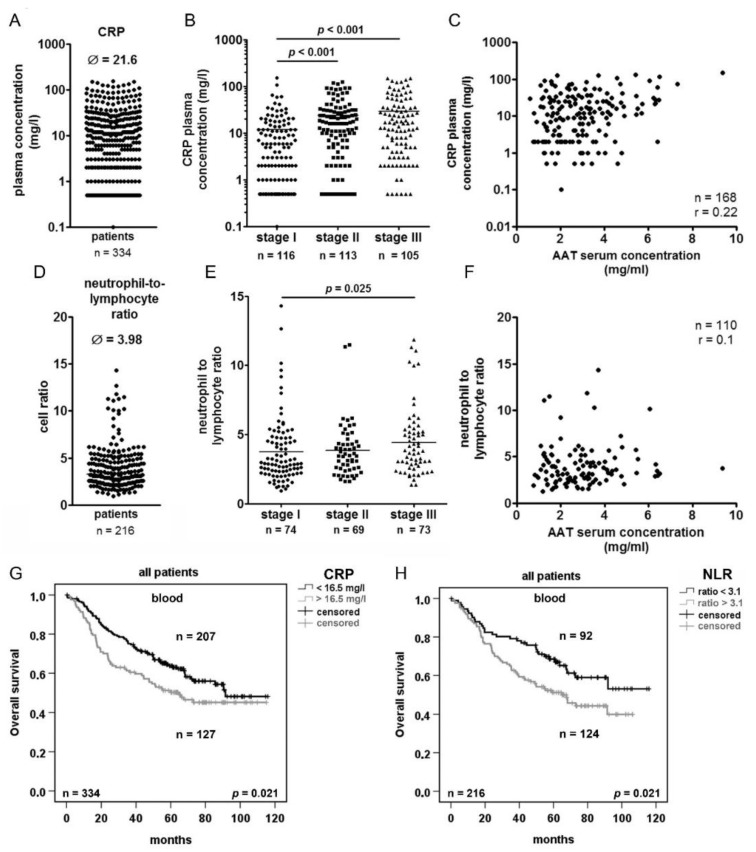

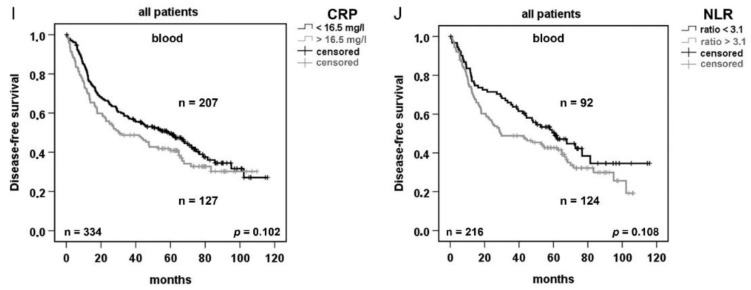
Elevated plasma CRP levels and a high neutrophil to lymphocyte ratio (NLR) are prognostic factors for the survival of NSCLC patients. (**A**) ELISA- based quantification of serum CRP levels in 338 NSCLC patients. (**B**) Serum concentrations of CRP in patients with cancer stage I, II and III. (**C**) Correlation between serum concentration of AAT and CRP. (**D**) Neutrophil-to lymphocyte ratio in 120 NSCLC patients. (**E**) Neutrophil-to-lymphocyte ratio in patients with cancer stage I, II and III. (**F**) Correlation between neutrophil-to-lymphocyte ratio and serum AAT concentration. (**G**) and (**I**) Kaplan-Meier overall and disease-free survival curves generated based on 16.5 mg/l cut-off of CRP to separate the two groups of patients. (**H**) and (**J**) Kaplan-Meier overall and disease-free survival curves generated based on cut-off of 3.1 NLR to separate the two groups of patients. *p* < 0.05 was considered significant. *r* > 0.5 was considered as correlation.

Because AAT is an acute phase protein, we wanted to investigate its relationship to other acute phase markers of inflammation and tumorigenesis such as CRP [22] and neutrophil-to-lymphocyte ratio (NLR) [23]. We analyzed serum CRP concentrations and NLR in 334 and 216 out of the 351 NSCLC patients, respectively. The median serum concentration of CRP was 21.6 mg/L and showed significant increase with pathological stage of the tumor (Figure 4A,B). Interestingly, we did not find any correlation between serum CRP and AAT concentrations or between serum CRP and *SERPINA1* gene expression in tumor or normal lung tissue of the corresponding patients (Figure 4C, Supplementary Figure S4A,B). On the other hand, based on the calculated cut-off of 16.5 mg/L CRP (Suppl. Figure S4C) we found that patients with a higher CRP (>16.5 mg/L) had significantly shorter overall but not disease-free survival (Figure 4G,I). In the same patients, the median NLR was 3.98 and showed significant increase with advanced tumor stages (Figure 4D,E). Similar like for CRP, we did not observe any correlation between NLR and AAT serum levels and *SERPINA1* gene expression (Figure 4F, Supplementary Figure S4D,E). However, patients with higher ratio of NLR (>3.1, Supplementary Figure S4F) showed significantly shorter overall, but not disease-free survival (Figure 4H,J).

### 2.5. Exogenous AAT Is Taken Up by NSCLC Cells and Regulates Key Hallmarks of Cancer Cells

In the next set of experiments, we aimed to investigate effects of *SERPINA1* and AAT protein levels in NSLC cell cultures, in vitro.

**Figure 5:**
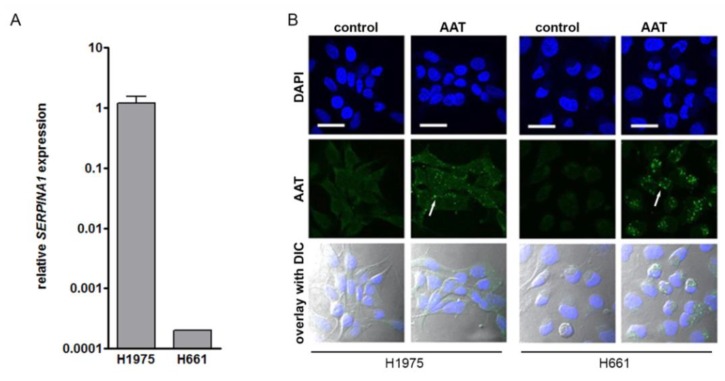

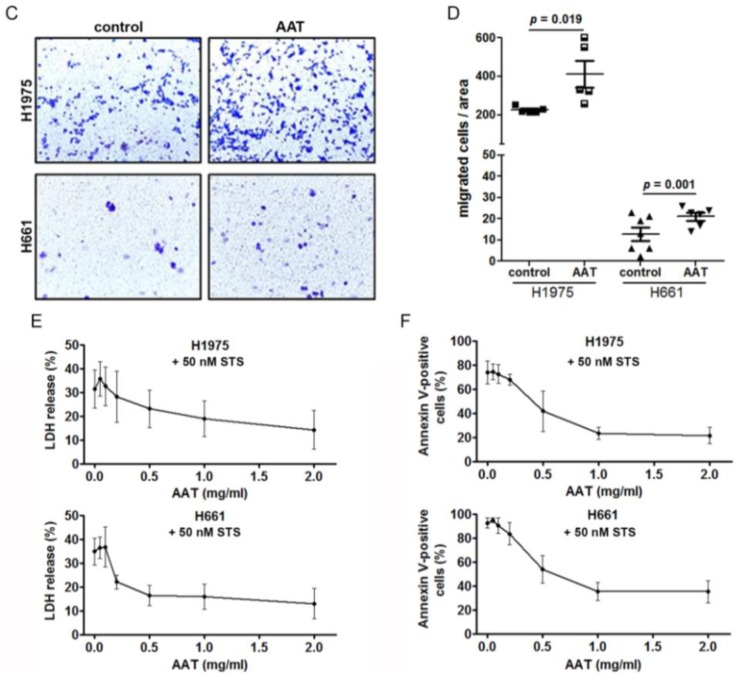
Exogenously added AAT induces cancer cell migration, increases viability and resistance against staurosporine-induced apoptosis. (**A**) *SERPINA1* expression relative to housekeeping gene *POLR2A* in H1975 and H661 cell lines. Data presented as the mean ± SD from three independent measurements. (**B**) Confocal immunofluorescence staining images of AAT protein (green) in H1975 and H661 in control and AAT (2 mg/mL) treated cells for 18 hours in serum-free medium. Nuclei were defined by DAPI (blue). Scale bar: 30 µm. (**C**) and (**D**) Migratory properties of H1975 and H661 cells without and with pre-treatment of AAT (2 mg/mL). The migrated cells were stained with 0.1% crystal violet. The graphs indicate data from three independent experiments as mean ± SD. *p* < 0.05 was considered as significant. (**E**) H1975 and H661 cells were treated with 50 nM staurosporine (STS) alone or together with AAT (0.05–2 mg/mL) for 18 hours and lactate dehydrogenase (LDH) release was measured. Each point is a mean ± SD from six independent experiments. (**F**) H1975 and H661 cells were treated with 50 nM STS alone or together with AAT (0.05–2 mg/mL) for 18 hours. Annexin V surface expression was determined by flow cytometry. Experiments were repeated four times. Each point represents a mean ± SD. *p* < 0.05 was considered as significant.

Based on the comparative analysis of 15 NSCLC cell lines for their *SERPINA1* gene and protein expression, we selected a cell line having high (H1975) and a low (H661) *SERPINA1* expression (Figure 5A and Supplementary Figure S5A,B). Since serum contains AAT, which can be taken up by cancer cells (Supplementary Figure S5C,D), all experiments were performed in serum-free media. Remarkably, despite the differences in the *SERPINA1* expression levels, untreated cell lines did not show a positive staining for AAT protein (Figure 5B). However, when exposed to the purified AAT (2 mg/mL) overnight, both H1975 and H661 showed a specific staining for intracellular AAT. Thus, cancer cells vary in the magnitude of *SERPINA1* gene expression at baseline, but independently on this, produce little of endogenous AAT protein. On the other hand, cancer cells can take up exogenous AAT.

Recent studies have linked AAT to invasion and metastasis in human cancers [24]. It is important to point out, that for functional studies, cancer cells were cultured in complete medium containing 10% fetal calf serum. Alike, our results show that H1975 having high baseline *SERPINA1* expression migrate markedly better than H611 cells having very low *SERPINA1* expression. Nevertheless, in the presence of exogenous AAT (2 mg/mL), both cell lines significantly enhanced (by approx. 50%) migratory properties (Figure 5C,D). H1975 and H661 cells lines were cultured for 18 h in the medium supplemented with different concentrations of AAT (from 0.05 to 2 mg/mL) alone or together with staurosporine (STS). As illustrated in Figure 5E, AAT inhibited lactate dehydrogenase (LDH) release in a concentration dependent manner, which reflects the lower disruption of plasma membrane and decreased cell death. In parallel flow cytometry experiments with annexin V/7AAD double staining revealed an elevated percentage of apoptotic cancer cells in the presence of STS, a substance that triggers cancer cell death via intrinsic apoptotic pathways. The pro-apoptotic effect of STS was markedly lowered with an addition of increasing amounts of AAT (Figure 5F and Supplementary Figure S5E).

### 2.6. Silencing or Overexpression of SERPINA1 Regulates Colony Formation, Migration and Survival of NSCLC Cell Lines

**Figure 6:**
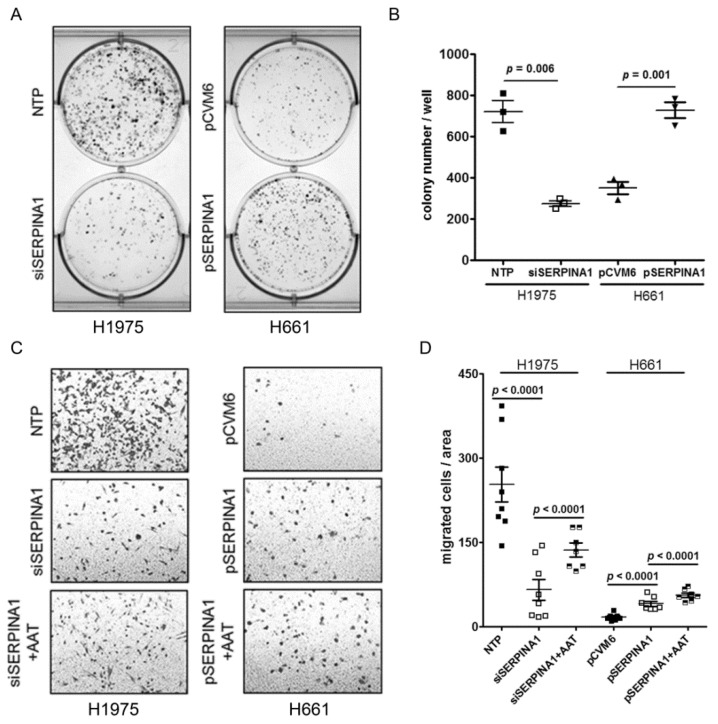

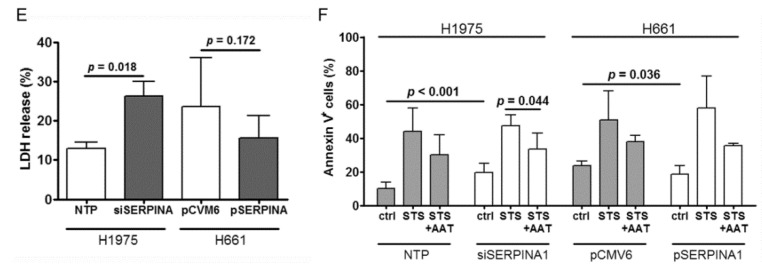
Silencing or overexpressing *SERPINA1* gene affects cancer cell colony formation, migration, viability and resistance to apoptosis. (**A**) and (**B**) Cancer colony number per area in *SERPINA1* siRNA-treated H1975 cells or *SERPINA1* overexpressing H661 cells. Each point represents a separate experiment. Data presented as mean ± SD. *p* < 0.05 was considered as significant. (**C**) and (**D**) The migratory abilities of H1975 cells after *SERPINA1*-knockdown and H661 cells after *SERPINA1* overexpression with/without addition of AAT. The graphs indicate data from three independent experiments as mean ± SD. *p* < 0.05 was considered as significant. (**E**) LDH release rate after silencing or overexpression of *SERPINA1*. H1975 and H661 cells were treated with 50 nM STS alone or together with AAT (2 mg/mL) for 18 h after *SERPINA1* knockout or overexpression, respectively. All experiments were performed in duplicates and repeated three times. Each point represents a mean ± SD. *p* < 0.05 was considered as significant. (**F**) H1975 and H661 cells were treated with 50 nM STS alone or together with AAT (2 mg/mL) for 18 h. Annexin V surface expression was determined by flow cytometry. All experiments were performed in duplicates and repeated three times. Each point represents a mean ± SD. p < 0.05 was considered as significant. NTP: non-target-pool siRNA and pCVM6 as control vector.

In the following experiments, we aimed to investigate the effect of *SERPINA1* gene knockdown in H1975 (*siSERPINA1*) and overexpression in H661 (*pSERPINA1)* cells, respectively. Cancer cell colony number per area decreased significantly in *siSERPINA1* H1975 cells but increased in *pSERPINA1* H661cells (Figure 6A,B). The average colony number from three experiments was reduced by about 62% in knockdown H1975 cells compared to the non-target-pool (NTP) -transfected cells (mean ± SD 720 ± 92 NTP vs 275 ± 24 *siSERPINA1*). By contrast, the mean colony number was increased by about 50% in *pSERPINA1* H661 as compared to the empty vector-transfected cells (mean±SD: 6 351 ± 51 pCVM vs 728 ± 66*p SERPINA1*).

As illustrated in Supplementary Figure S6A,B, we were able to successfully silence the *SERPINA1* gene in H1975 cells and to increase its expression in H661 cells. Accordingly, cell-associated AAT protein significantly increased in H661 cells and decreased in H1975 (Supplementary Figure S6C). Noticeably, interference with *SERPINA1* gene expression was not possible for cells cultured in serum-free medium. Therefore, cell lysates prepared from control cells are positive for AAT, which reflects cell-associated, serum derived AAT (Supplementary Figure S6C). In fact, cancer cells grown in serum containing media were always positive for AAT protein independently of *SERPINA1* expression levels (Supplementary Figure S6C).

As shown in Figure 6C,D, siRNA treated H1975 cells significantly lost migratory properties relative to the control cells whereas the migratory properties were to the large extend restored in the presence of exogenous AAT (2 mg/mL) (Figure 6D). By contrast, *SERPINA1* overexpressing H661 cells strongly improved migratory properties (2.4-fold, *p* < 0.001) relative to controls and even more strongly exaggerated migration in the presence of AAT (Figure 6D).

According to LDH assay, siRNA treated H1975 cells showed significantly higher LDH release relative to control-treated cells (Figure 6E). In parallel, annexin V analysis revealed a lower viability of silenced cells compared to controls (mean ±SD, 79.54 ± 2.47 vs 90.27 ± 2.14, *p* < 0.001). The treatment with STS resulted in a similar rate of cell apoptosis in both conditions whereas STS effect was reduced in the presence of exogenous AAT (Figure 6F and Supplementary Figure S6D). The *SERPINA1* overexpressing H661 cells did not show significant difference in the LDH release if compared to controls but the apoptosis rate was about 15% higher. Again, exogenously added AAT lowered STS-induced cell apoptosis (Figure 6F and Supplementary Figure S6E).

### 2.7. Genes Most Related to SERPINA1 in H1975 and H661 Cells 

Finally, we expanded our analysis to investigate a putative relationship of *SERPINA1* to specific inflammation, proliferation and apoptosis-related genes. For this, we performed RNA-seq of H1975 and H661 cells cultures, both untreated and in the presence of AAT (2 mg/mL). Using GFOLD for single-replicate analysis, we found 5778 and 8879 candidate genes for AAT response in H661 and H1975 cells, respectively (GFOLD values not equal to 0). We ran the gene enrichment analysis on PANTHER to get the significantly enriched GO (Gene Ontology) BP (biological process) terms induced by the treatment with AAT. This analysis generated a list of 681 significant GO terms for the H661 and 949 for the H1975 cells (Supplementary Table S1). The GO enrichment analysis results contained general terms such as cellular process, metabolic process or non-specific biological regulations not particularly related to tumorigenesis. Therefore, the lists were further shortened semantically to ones only consisting GO terms specifically related to our experimental observations in inflammation, apoptosis, and migration. This selection trimmed the list to 15 and 36 GO terms for H661 and H1975, separately.

**Figure 7:**
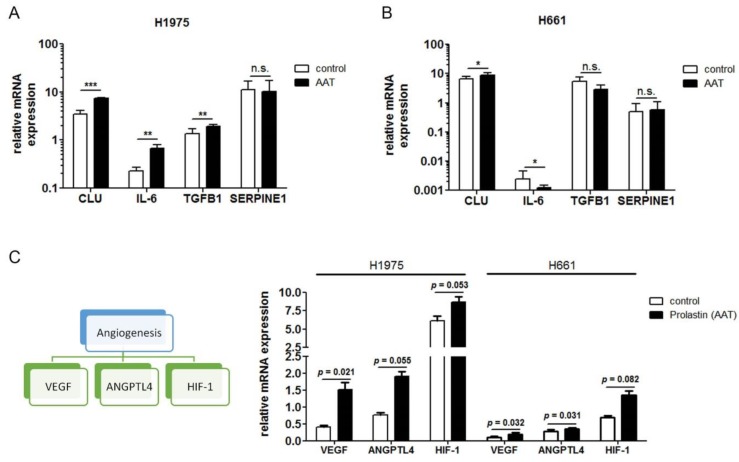
Validation of AAT-regulated genes in H1975 and H661 by qPCR. (**A**) and (**B**) Gene expression relative to housekeeping gene *POLR2A* of selected genes from RNA-seq analysis of H1975 and H661. Data presented as the mean ± SD from three independent measurements. (**C**) Gene expression relative to housekeeping gene *POLR2A* of genes involved in angiogenesis and hypoxia. * *p* < 0.05, ** *p* < 0.005, *** *p* < 0.001.

The PPI (protein-protein interaction) networks of the genes in these selected GO terms and SERPINA1 were analyzed by String DB to understand the potential relationship between genes within the processes and the SERPINA1 gene. To increase the reliability of the putative genes, the confidence level of the PPI was set to the highest of 0.9000 and only database sourced interactions were chosen for the network. To further eliminate the cascade processes not directly regulated by the SERPINA1 gene, only the neighbor nodes on the network were selected for the interpretation. Four enriched GO terms, namely inflammatory response, cell population proliferation, apoptotic process and regulation of inflammatory response, involved in the AAT treatment of H661 cell-line and consisted putative DE genes directly interacted with SERPINA1. For H1975 cell-line, inflammatory response, apoptotic process, positive regulation of apoptotic process and regulation of inflammatory response were significantly enriched and equally covered putative DE genes neighboring to SERPINA1. The detailed information is available in Table 3 A and B. Interestingly, we found five common *SERPINA1* related genes, namely *APOE, APP, CLU, SERPINE1 and SHISA5* in both cell lines. Moreover, as shown in Table 3, in H1975 cells, *SERPINA1* expression was related to the upregulation of *TGFB1* and *TGFB2* whereas in H661, having almost no *SERPINA1* expression, relationship was towards downregulation of *TGFB1* and *TGFB2.* We therefore investigated the expression of interesting genes in H1975 and H661 by qPCR and could validate our findings for CLU, IL-6 and TGFB1 (Figure 7A,B). According to RNA-seq analysis, some genes related to tumor angiogenesis and hypoxia were induced by the treatments with AAT. Therefore, we validate few genes by RT-PCR. As illustrated in Figure 7C, AAT markedly upregulates hypoxia and angiogenesis-related genes like *HIF1*, *ANGPTL4* and *VEGF* expression in H1975 and H661 cells.. Thus, AAT might protect cancer cells from apoptosis during oxygen deprivation.

## 3. Discussion

The link between inflammation and the development of cancer is an area of growing interest in cancer research. Chronic inflammation has been associated with tumor initiation, invasive potential and progression [25]. An increase in serum levels of acute-phase proteins, as markers of inflammation, has been reported in various forms and stages of cancer, including lung cancer [26,27]. Furthermore, a positive correlation has been shown between serum levels of acute phase proteins and tumor progression, alongside with a negative prognosis. On the other hand, the alterations in acute phase protein concentrations are observed during a wide range of inflammatory conditions, therefore many researchers are skeptical to consider these proteins as specific biomarkers for cancer. Despite that, proteomic studies continue to provide evidence that acute phase proteins, like CRP and AAT, could be useful biomarkers in early detection of lung cancer and in monitoring its evolution [12].

To the best of our knowledge, *SERPINA1* gene expression and serum levels of AAT protein have not been investigated as independent prognostic markers in lung cancer. To address this limitation, we focused on the *SERPINA1* gene expression in tumor and adjacent non-tumor lung tissues and on the tumor tissue and serum levels of AAT protein in samples obtained from more than 300 patients enrolled in a prospective longitudinal study at the Thorax Clinic of University Hospital Heidelberg (Germany). According to our findings, higher serum levels of AAT in NSCLC patients are prognostic for the patient’s worse outcome but do not correlate with *SERPINA1* expression in tumor or non-tumor lung tissue or with staining intensities for tumor-related AAT protein. Previous data have shown that AAT levels may depend on BMI [28]. However, in our cohort we did not find any relationship between BMI and *SERPINA1* and AAT protein levels (data not shown). Unfortunately, in our cohort of cancer patients, we do not have the complete data on chronic inflammatory diseases, such as rheumatoid arthritis, inflammatory bowel diseases, cardiovascular diseases, diabetes and others. Therefore, we were not able to investigate any putative relationships with AAT.

It is known that protein measurements are not necessarily mirrored by gene expression, and that protein and protein-coding gene can reflect different processes in tumor biology [29,30]. The AAT gene, *SERPINA1*, expresses a number of mRNA isoforms generated by alternative splicing in the 5′-untranslated region (5′-UTR). Although all *SERPINA1* mRNAs encode the same protein, expression levels of the individual mRNAs vary substantially in different human tissues. In fact, the *SERPINA1* gene belongs to the top 0.5% of human genes in terms of transcriptional complexity—the eleven different splicing isoforms occur in human tissues [31]. The amount of AAT protein produced from a gene is not a simple function of the abundance of the transcript [32]. Therefore, we focused our investigations on *SERPINA1* expression and AAT protein, as individual putative prognostic markers in NSCLC.

Data from lung tissue analysis clearly show that the expression levels of *SERPINA1* are highly variable but significantly lower in tumor tissue compared to adjacent normal lung tissue. This was true for both squamous cell carcinoma (SQCC), and adenocarcinoma (ADC) subtypes of NSCLC. Regarding the prognostic value of *SERPINA1*, higher expression of this gene in tumor tissue, especially in current smokers, was associated with better overall and disease-free survival. Whereas in non-neoplastic tissues was an opposite- lower expression of *SERPINA1* turned to be significantly associated with better overall and disease-free survival. The Kaplan Meier survival analysis based on the ratio between relative *SERPINA1* expression in tumor and paired non-tumor lung tissue confirmed that overall and disease-free patient`s survival is better when tumor vs non-tumor ratio for *SERPINA1* is higher. Based on these findings, we hypothesize that a high tumor/non-tumor expression ratio of this gene may indicate the inverse relationship between the signatures of tumor and non-tumor signaling pathways. For example, one of the hallmarks of any tumor-associated environment is the lack of sufficient tissue vascularization, which leads to hypoxic conditions and the activation of hypoxia-associated pathways in tumor cells as well as in infiltrating immune cells [33]. It is well- documented that in the hypoxic regions cancer cells switch on an adaptive responses mediated primarily by the hypoxia-inducible factors (HIFs) [34]. The HIF-driven transcriptional response is of critical importance for hypoxic adaptation, however still remains not totally clear which genes downstream of HIFs are most critical for cell survival. For example, Greenhough and co-authors recently identified that HIFs are activators of GPRC5A transcription (G Protein-coupled Receptor Class C, Group 5, Member A), a new mediator of cancer cell survival during hypoxia [35]. Vascular endothelial growth factor (VEGF) and angiopoietin-like 4 (ANGPTL4) are also HIF-1-regulated angiogenic genes playing a pivotal role in tumor invasiveness and metastasis [36]. We previously found that AAT induces *ANGPTL4* and *HIF-1* expression in human adherent PBMCs [37,38]. Here we show that AAT significantly upregulates *ANGPTL4* and *VEGF* expression in H1975 and H661 cells.

Indeed, AAT might be a novel hypoxia-induced protein that functions to protect cancer cells from apoptosis during oxygen deprivation. If *SERPINA1* expression and AAT levels relate to hypoxia during tumor development in vivo, warrants further studies. In fact, *SERPINA1* expression has been shown to increase during hypoxic conditions [30], and hypoxia-related tissue damage during ischemia-reperfusion injury, stroke, ischemic heart disease has been lowered by the therapy with AAT [39]. On the other hand, systemic inflammation, which assists tumor grow, is related to the activation of IL-6 and IL-1*β* pathways which are responsible for induction of *SERPINA1* expression. Thus, systemic inflammation and tumor microenvironment may reflect higher tumor/non-tumor expression ratio of *SERPINA1* gene. In support, a recent study by Hei Jason Chan et al. [40] reported that although the expression level of *SERPINA1* was higher in endocrine resistant cells than responsive breast cancer cells, the Kaplan Meier survival analysis revealed that high expression of this gene is associated with better survival in the estrogen receptor alpha resistant breast cancer. These findings point to a potentially multifaceted mechanism involved in *SERPINA1* expression regulation, which should be studied further.

Since tumor tissue used for *SERPINA1* expression analysis contains not only tumor but also other cell types expressing *SERPINA1* gene, we performed immunostainings for AAT protein in tumor tissues. A large variability in staining patterns and intensity for AAT protein did not relate to overall survival of the patients. However, by introducing the cut-off value of a staining score, we saw a tendency towards the lower tumor-AAT staining and a better overall and disease-free survival of NSCLC patients. This result is not so easy to interpret because AAT is one of the most abundant proteins in human circulation and is commonly present in all tissues. Our *in vitro* experiments clearly show that lung cancer cells highly differ in *SERPINA1* expression levels and can uptake exogenous AAT protein. Therefore, we thought that higher number of AAT-positive cancer cells may reflect higher levels of circulating AAT.

Previous studies linking higher levels of serum AAT with a worse lung cancer prognosis [41], prompted us to analyze serum levels of AAT and to correlate them with *SERPINA1* expression and AAT immunostainings in tumor and adjacent normal lung tissues. Serum samples collected at the time of diagnosis were available from 170 NSCLC patients and showed 2.68 mg/mL median level of AAT. Unfortunately, serum levels of AAT did not correlate either with *SERPINA1* expression or with AAT protein staining in lung tissues. Our results contrast the paradigm that lung tissue derives its AAT mainly from the bloodstream [42]. The detection of *SERPINA1* transcripts in cultured lung cancer cells and the quantification of *SERPINA1* expression in tumor and adjacent non-neoplastic lung tissue support the conclusion that cells in the lung express AAT protein itself. With an aim to investigate whether the differences in serum AAT levels have any prognostic value, we introduced 2.66 mg/mL as a serum cut-off value of AAT. In agreement with previously reported data, higher AAT levels were associated with worse overall patient survival and with more advanced pathological tumor stage. Even though higher levels of AAT were linked to the worse prognosis, in general AAT levels decreased with advanced tumor stages. Recent study by Boccellino et al., also reported a progressive decline in serum AAT concentration starting from health to advanced lung cancer, in opposition to increased inflammatory response as demonstrated in other studies [43]. The reason for this observation remains under investigations.

In general, our findings support a notion that different pathways of inflammation may follow tumor development [44]. The changes in systemic levels of AAT probably reflect early lung cancer stages and the progression rather when chronic inflammation *per se*. This concept is in part supported by two findings: i) CRP levels and NLR increase with advanced cancer stages while levels of AAT decrease, and ii) serum CRP and AAT, and NLR and AAT did not correlate. The relationship between AAT and the lung cancer can be dual sided. First, tumor progression itself can cause systemic inflammation resulting in increased circulating levels of AAT together with other acute phase proteins [45]. Second, cancer cells and cancer-associated cells express *SERPINA1* gene themselves and can also uptake exogenous AAT, which may help to develop a favorable microenvironment for the tumor progression. Similar scenario has been suggested for CRP in lung cancer [46].

During the chronic inflammation, which is a driving force in tumor development, *SERPINA1* expression and AAT levels increase substantially and, together with other inflammatory molecules, may activate tissue repair responses, induce proliferation of premalignant cells and enhance their expansion. This may also trigger tumor`s intrinsic inflammatory response that builds up a pro-tumorigenic microenvironment [47]. We therefore sought to determine whether increased *SERPINA1* expression and AAT protein levels affect lung cancer cell behavior *in vitro*. For these experiments we selected two NSLC cell lines having fundamentally low (H1661) and high (H1975) *SERPINA1* expression levels. Remarkably, both cancer cells, independently on *SERPINA1* expression levels, have taken up exogenous AAT which was found to be localized in cytosol. The presence of AAT in the cytosol after incubation with exogenous AAT has also been reported in lung endothelial cells, human monocyte-derived macrophages, primary rat alveolar macrophages, neutrophils, CD4+ T cells, mesenchymal stem cells, Min6 cells and human islets [48,49,50,51]. Thus, like other cells, cancer cells can uptake AAT from their environment, which according to our results will favor cell viability, resistance to apoptosis and migration. Similarly, studies in gastric, ovarian, breast, colorectal, and lung cancer cells have shown that AAT promotes tumor cell migration and invasion capacity [40,52,53,54,55]. According to our results, not only the uptake of exogenous AAT protein but also increased expression of *SERPINA1* gene significantly enhances lung cancer cell migration, viability and resistance towards staurosporin-induced apoptosis. However, as mentioned above, higher *SERPINA1* expression within a tumor tissue is related to better patient`s prognosis. This contradiction between results in tumor tissues and in cell lines remains to be addressed in further studies.

Several tumorigenic mechanisms related to higher AAT levels can be suggested. For example, AAT mediates anti-apoptotic effects through the TNFα-signaling pathway or directly via blocking caspase-3 activity [56]. AAT also interacts with NF-κB-TNF*α*-axis, an important pathway in cancer development [57]. Furthermore, AAT has been found to increase the expression of fibronectin and promote cancer cell migration [55]. Seung-Hee Chang and co-authors have used proteomic analyses to compare protein levels following AAT overexpression in normal lung cells containing fundamentally low level of AAT. These authors found that an overexpression of AAT increase levels of proteins involved in transcription and translation, such as signal transducer and activator of transcription 5B (STAT5B) and eukaryotic translation elongation factor 1-alpha 2 (EEF1A2). Moreover, overexpression of AAT inhibited the expression of the autophagy protein, BECN1, thereby possibly increasing cell survival [58].

Our findings illustrate the significance of *SERPINA1* expression and AAT protein levels in lung cancer, however, detail investigations on the mechanisms behind the regulation of *SERPINA1* expression and AAT protein production in tumor cells are beyond the scope on this work. Presently, based on the preliminary RNA-seq data obtained from H1975 and H661 cells exposed to AAT protein, we can only suggest few putative pro-tumorigenic pathways linked to *SERPINA1* gene and AAT protein. Although the significant GO terms identified by PANTHER platform are not identical in H661 and H1975 cell-lines, both results highlight several inflammation-, apoptosis- and proliferation-related GO terms, which are consistent with our observations in the experiment. The constructed PPI network indicates few tightly connected interactions between differential expression of genes in these GO terms and *SERPINA1* gene in the form of direct protein-protein interaction. The significant GO terms of inflammatory response and regulation of inflammatory response as well as the significant upregulation in inflammatory-related genes such as *APOE*, *APP* and *SERPINA1* all putatively suggest that the tumorigenesis of AAT might be partially caused by its increased levels during inflammation.

AAT possesses anti-inflammatory activity against immune cells, affects leukotriene B4, TNF-*α*, IL-8, IFN-*γ* and IL-1*β* production and release, and expresses potent anti-apoptotic properties [15,59]. In addition, AAT has been shown to inhibit the activity of natural killer cells against tumor cells [60], which indicates that in a situation of occurring cancer cells higher AAT levels may favor microenvironment towards malignant progression.

Taken together, the relation of *SERPINA1* gene expression, AAT tumor levels and patients’ serum concentrations in the situation of an acute NSCLC is very complex. Nevertheless, since AAT reflects the inflammatory situation and shows a high influence on survival prognoses, further investigations shall clarify how levels of AAT are regulated in healthy lung and tumor tissue, and if can be considered as prognostic marker and target for NSCLC.

## 4. Materials and Methods

### 4.1. Sample Collection, Characterization and Preparation

Tissue samples were provided by the Lung Biobank Heidelberg, a member of the accredited Tissue Bank of the National Center for Tumor Diseases (NCT) Heidelberg, the Biomaterial Bank Heidelberg, and the Biobank platform of the German Center for Lung Research (DZL). The use of biomaterial and data for this study was approved by the local ethics committee of the Medical Faculty Heidelberg (S-270/2001). All patients included in the study signed an informed consent and the study performed according to the principles set out in the WMA Declaration of Helsinki. Tumor and matched distant (>5 cm) normal lung tissue samples from NSCLC patients who underwent therapy-naive resection for primary lung cancer at Thoraxklinik at University Hospital Heidelberg, Germany were collected between 2006 and 2011. Tissues were snap-frozen within 30 minutes after resection and stored at −80 °C until the time of analysis. More detailed information is described elsewhere [61]. Serial blood sampling is conducted at time point of diagnosis prior to surgical intervention. Blood was collected and processed within 1 h. Serum aliquots were stored at −80 °C until measurements. The patient cohort is described in Table 1. All following measurements were performed using the whole cohort or parts of it.

### 4.2. Total RNA Isolation and cDNA Synthesis 

For RNA isolation from patient tumor tissue, a tumor content of ≥50% was the minimum prerequisite. A total of 10–15 tumor cryosections (10–15 µm) from each patient were sliced, and the first as well as the last section of the series was stained with H&E. A lung pathologist determined the proportion of viable tumor cells, stromal cells, healthy lung cells, and necrotic areas. Total RNA was isolated from patient tissue using an AllPrep DNA/RNA/miRNA Universal kit (Qiagen, Hilden, Germany) according to the manufacturer’s instructions. An RNeasy Mini kit (Qiagen) was used to isolate RNA from the cell lines. Afterwards, the quality of total RNA was assessed by utilizing an Agilent 2100 Bioanalyser and an Agilent RNA 6000 Nano kit (Agilent Technologies, Boeblingen, Germany). With the Transcriptor First Strand cDNA Synthesis kit (Roche, Basel, Switzerland), total RNA was transcribed to complementary DNA and used for quantitative polymerase chain reaction (qPCR). A complete description of the procedure is provided elsewhere [62]. Total RNA from lung cancer cells was isolated using the RNeasy Mini Kit (Qiagen) following the manufacturer’s instructions. RNA was reverse transcribed with High Capacity cDNA Reverse Transcription Kit (Applied Biosystems, Thermofisher Scientific, Waltham, MA, USA).

### 4.3. Real-Time Polymerase Chain Reaction (RT-PCR) Analysis

For gene expression analyses of patient tissues, volumes of 5 µL cDNA (corresponding to 5 ng of isolated total RNA) were utilized for qPCR with the LightCycler480^®^ (Roche) in 384-well plates according to the Minimum Information for Publication of qPCR Experiments (MIQE) guidelines [63]. Universal ProbeLibrary (UPL) assay (Roche) was used as the amplification and detection system. Gene-specific primers (TIB Molbiol, Berlin, Germany) were combined with the primaQuant 2 × qPCR Probe-MasterMix (Steinbrenner Laborsysteme, Wiesenbach, Germany). Threshold cycle (Ct) values were evaluated with the LightCycler480^®^ software release 1.5 and the 2nd derivative maximum method (Roche). For the comparison of gene expression in tumor and non-malignant samples, the relative expression of the genes (normalized to the housekeepers Esterase D (POLR2AESD) and Ribosomal Protein S18 (RPS18)) was calculated (ΔCt values). The following primers and UPL were used for the detection of *SERPINA1*:

*SERPINA1 forward (UPL #82, 5′-AATGGGGCTGACCTCTCC-3″),*

*SERPINA1 reverse (UPL #82, 5′-GTCAGCACAGCCTTATGCAC-3″),*

*ESD forward (UPL#50, 5′-TCAGTCTGCTTCAGAACATGG-3′),*

*ESD reverse (UPL#50 5′-CCTTTAATATTGCAGCCACGA-3′),*

*RPS18 forward (UPL#46, 5′-CTTCCACAGGAGGCCTACAC-3′),*

*RPS18 reverse (UPL#46, 5′-CGCAAAATATGCTGGAACTTT-3′).*

The complete procedure is described elsewhere [62]. For the analyses of cDNA from lung cancer cell lines the following assayswere used: SERPINA1 Assay ID: Hs00165475_m1; CLU: Hs00156548_m1; IL6: Hs00985639_m1; TGFB1: Hs00998133_m1; ANGPTL4: Hs01101127_m1, SERPINE1: Hs01126606_m1, HIF-1: Hs00153193_m1, VEGFA: Hs00900055_m1 and, POLR2A (Housekeeper) Assay ID Hs00172187_m1 (Applied Biosystems, Thermofisher Scientific, Waltham, MA, USA). Results were visualized with TaqManTM Gene Expression Master Mix (Applied Biosystems) using the fluorescence reader StepOne Real-Time PCR Systems (Applied Biosystems). The following amplification profile program was used: enzyme activation at 95 °C [10 min] followed by 40 cycles of a two-step PCR; 95 °C [15 s], 60 °C [60 s]. Data were normalized to *POLR2A,* and relative mRNA level (ΔCt) was calculated. 

### 4.4. Immunohistochemistry (IHC)

For the detection of AAT, the monoclonal AAT antibody (sc-59438, Santa Cruz Biotechnology, Heidelberg, Germany) was used. Before tissue micro array (TMA) construction, a hematoxylin and eosin (H&E)-stained slide of each block was analyzed to select tumor-containing regions. A TMA machine (AlphaMetrix Biotech, Roedermark, Germany) was used to extract tandem 1.0-mm cylindrical core sample from each tissue donor block. Paraffin-embedded tissue sections were deparaffinized with the following steps: 2 × 10 min in xylol, 2 × 5 min in 100% ethanol, 1 × 3 min in 98% ethanol and 1 × 3 min in 70% ethanol. Antigen retrieval was performed in a steamer with sodium-citrate-buffer (10 mM sodium citrate, 0.05% Tween 20, pH 6.0) for 15 min. Peroxidases were blocked for 10 min at room temperature (RT) using 3% H_2_O_2_ (Applichem, Darmstadt, Germany). Slides were incubated with normal goat serum for 1 h at RT to avoid unspecific background staining (Cell Signaling, Frankfurt, Germany). First antibody (sc-59438, Santa Cruz Biotechnology) was incubated over night at 4 °C in a humid chamber. The staining procedure was performed with SignalStain^®^ DAB Substrate Kit (#8059, Cell Signaling) according to manufacturer’s instructions. The last developing step was performed for 2 min. Cell nuclei were stained using Mayer’s Hematoxylin Solution (Sigma-Aldrich, Munich, Germany). Slides were mounted using ImmunoHistoMount^TM^ (Sigma-Aldrich). Staining was observed with an Olympus IX-71 inverted microscope. Pictures were taken with an Olympus Color View II digital camera and Olympus Cell-F software (cellSense dimension, V1.11, Olympus, Hamburg, Germany). Tiffs were assembled into figures using Photoshop CS6 (Adobe, San José, CA, USA). Only changes in brightness and contrast were applied. TMA slides were scanned and analyzed using Aperio ImageScope (v12.3.2.8013, Leica Biosystems, Wetzlar, Germany). Scoring was performed by multiplication of staining intensity (0-3) with the proportion of positive cells (0–4) (see Figure 2A).

### 4.5. Analysis of Serum AAT Protein Concentration

The levels of serum AAT were analyzed by using an enzyme-linked immunosorbent assay kit (Human Alpha-1-Antitrypsin ELISA Kit, Abbexa Ltd, Cambridge, UK) with 100 µL of each serum in two technical replicates according to the manufacturer’s instructions. The readout and standard curves were performed with an ELISA Reader (Tecan Group Ltd., Crailsheim, Germany). The results of the ELISA were visualized with GraphPad Prism 5 (GraphPad Software, San Diego, CA, USA).

### 4.6. CRP, Neutrophil and Lymphocyte Analysis

The levels of serum CRP (mg/L) were measured during clinical routine using Siemens Dimension ExL with LM (Siemens, Erlangen, Germany) and the Siemens CRP_2 Kit (#11097631, Siemens). Absolute neutrophil and lymphocyte number was determined using a Siemens ADVIA 2120i system.

### 4.7. Cell Culture Experiments

The squamous cell carcinoma cell lines 2106T and 2427T (in-house derived cell lines) were cultivated in DMEM/Ham’s F-12 (Thermo Fisher Scientific, Schwerte, Germany) with 10% fetal bovine serum (Gibco^®^ Thermo Fisher Scientific, Waltham, MA, USA). The primary cell lines 4950T, 170162T, 161652N, and 161683N (in-house derived) were cultivated in serum-free DMEM/Ham’s F-12 with epithelial airway growth factors (Promocell, Heidelberg, Germany) and ROCK inhibitor (Rho-associated, coiled-coil containing protein kinase inhibitor; Stemcell Technologies, Cologne, Germany).

NSCLC cell lines NCI-H460 (ATCC^®^ HTB-177™), NCI-H520 (ATCC^®^ HTB-182™), NCI-H661 (ATCC^®^ HTB-183™ Lot No. 63087049), NCI-H838 (ATCC^®^ CRL-5844™), NCI-H1299 (ATCC^®^ CLR-5803™ Lot No. 63777499), NCI-H1437 (ATCC^®^ CRL-587™ Lot No. 62818189), NCI-H1563 (ATCC^®^ CRL-5875™ Lot No. 61465128), NCI-H1573 (ATCC^®^ CRL-5877™ Lot No. 62486980), NCI-H1650 (ATCC^®^ CRL-5883), NCI-H1869 (ATCC^®^ CRL-5900™), NCI-H1975 (ATCC^®^ CRL-5908™ Lot No. 70000787), NCI-H2126 (ATCC^®^ CCL-256™ Lot No. 63493587), H2228 (ATCC^®^ CRL-5935™), A549 (ATCC^®^ CCL-185™) and HCC827 (ATCC^®^ CRL-2868™) were purchased from ATCC the Global Bioresource Center (Manassas, VA, USA). Tests for mycoplasma (Hoechst DNA stain, Agar culture, PCR-based assay) were performed by the supplier. HCC-15 cells (DSMZ #ACC 496) were purchased from German Collection of Microorganisms and Cell Cultures GmbH (Braunschweig, Germany). Cells were maintained in RPMI 1640 with fetal bovine serum or DMEM/F12 supplemented with insulin, L-glutamine (Gibco^®^ Thermo Fisher Scientific), transferrin, sodium selenite, hydrocortisone, beta-estradiol (Sigma-Aldrich, St. Louis, MO, USA), and fetal bovine serum according to the protocol of the supplier at 37 °C and 5% CO_2_. Cells from passages 3 to 15 were used for experiments. During experiments cells were cultured in serum-free media.

### 4.8. SERPINA1 Gene Knockdown and Overexpression

For transient depletion of *SERPINA1*gene, we used a pool of four *SERPINA1* siRNAs: J-008847-05 (5′-GAACUCACCCACGAUAUCA-3′), J-008847-06 (5′GAUGAAGCGUUUAGGAUG-3′), J-008847-07 (5′-CCUAUGAUCUGAAGAGCGU-3′) and J-008847-08 (5′-CCAAGAAACAG AUCAACGA-3′). As control, ON-TARGET plus Non-Targeting Pool (D-001810-10-05) was used. siRNA transfections were performed using Lipofectamine 2000 (Invitrogen, Thermo Fisher Scientific, Waltham, MA, USA) according to the manufacturer’s instructions. For overexpression of *SERPINA1*, cells were transfected with the pCMV6-SERPINA1 (SC119951, OriGene, Rockville, MD, United States) vector or with the pCMV6 (PS100020, OriGene) vector as a control using Lipofectamine 2000 (Invitrogen) according to the manufacturer’s protocol. Transfection medium were replaced after 12 h and cells were used for experiments 72 h after transfection. 

### 4.9. Western Blots

Cells were lysed in RIPA buffer (20 mM Tris-HCl pH 7.5, 150 mM NaCl, 9.5 mM EDTA, 1% Triton X-100, 0.1% SDS, and 1% sodium deoxycholate) (Sigma-Aldrich) supplemented with protease inhibitor cocktail (Sigma-Aldrich). Protein concentrations were assessed using the bicinchoninic acid (BCA) assay and protein contents were measured using a Tecan Infinite 200 PRO (Männedorf, Switzerland). Equal amounts of proteins were separated by 7.5% or 10% SDS-polyacrylamide gels prior to transfer onto a polyvinylidene difluoride (PVDF) membranes (Millipore, Billerica, MA, USA). Membranes were blocked for 1 h with PBS containing 5% low fat milk powder (Roth, Karlsruhe, Germany) followed by overnight incubation at 4 °C with primary polyclonal rabbit anti-human AAT (1:800) (DAKO, city, Denmark) and anti-*β*-actin 1:20.000 (Sigma-Aldrich) in blocking solution. The immune complexes were visualized with horseradish peroxidase–conjugated antibodies (DAKO A/S) and ECL western blotting substrate (Thermo Fisher Scientific) in Chemidoc Touch imaging system (BioRad, Hercules, CA, USA).

## 5. Functional Cell Assays

### 5.1. Analysis of Intracellular AAT

Cells were grown on coverslips. At 70% of confluency, cell growth medium was replaced with serum free medium with or without AAT (2 mg/mL), and cell were incubated for 18 h. Afterwards, cells were fixed with 4% paraformaldehyde for 30 min at room temperature, permeabilized with 0.1% Triton X-100 in PBS for 10 min , blocked for 30 min with blocking solution (0.3% glycine, 1% bovine serum albumin (BSA), 5% goat-serum, 0.1% Tween-20 in PBS) and incubated for 1 h with primary mouse monoclonal anti-human AAT antibody at room temperature in blocking solution. Coverslips were washed 3 times with PBS, followed by the incubation for 1 h at room temperature with AlexaFluor-488-labeled goat anti-mouse IgG (1:400 dilution, Thermo Fisher Scientific). The cells were then washed in PBS and mounted on microscope slides using DAPI containing medium (counterstained with 4′-6-diamidino-2-phenylindole, Thermo Fisher Scientific). Images were made with an Olympus FluorVIew 1000 scanning confocal microscope equipped with a 60 × oil immersion objective.

### 5.2. Cell Migration

Cellular migration was determined by the Transwell assay using 8-μm pore size membrane filters in 24-well chemotaxis chambers (Corning™ Transwell™ PC inserts, Corning, NY, USA). After 24 h culture in serum free medium cells were trypsinized with Trypsin/EDTA 0.025% solution (Lonza), suspended in serum-free medium and added to the upper chamber (1 × 10^4^ cells, 250 μL). Media supplemented with 10% FCS was added to the lower chamber. Following incubation at 37  °C for 48 h, the cells migrated to the lower side of the insert were fixed in 100% methanol and stained with 0.1% crystal violet. For quantification, stained cells were counted under a microscope (Leica DM750 equipped with Leica ICC50 HD camera, Leica Microsystems) with 10x objective from 6 fields. Experiments were performed in biological triplicates.

### 5.3. Lactate Dehydrogenase (LDH) Cytotoxicity Assay 

Native or transfected/transduced cells were incubated alone and with staurosporin (STS, Sigma-Aldrich) without or with addition of AAT protein for 18 h. Afterwards plates were immediately set on ice, 100 μL culture media was collected for each condition and centrifuged at 300× *g* for 5 min 4 °C. The LDH release was measured using the LDH-Cytotoxicity Detection Kit (Roche) and analyzed on an Infinite 200 PRO Microplate reader (Tecan). Individual samples were run in duplicate. Experiments were repeated three times. 

### 5.4. Apoptosis Assay

Cancer cells were treated with 50 nM STS alone or together with AAT protein in the concentrations as indicated (0.05–2 mg/mL) for 18 h. Afterwards, cells were assessed using the PE Annexin VApoptosis Detection Kit (BD Pharmingen™, Franklin Lakes, NJ, USA) according to the manufacturer’s instructions. Briefly, the cells were harvested by trypsination and incubated with PE Annexin V and 7-AAD for 15 min in the dark. Cell apoptosis was analyzed in a flow cytometer (Guava^®^ easyCyte Flow Cytometer, Lunimex, Austin, TX, USA). Experiments were performed in duplicates and repeated three times. 

### 5.5. Cancer Colony Forming Assay

Cells (1000 cells/well) were seeded in six-well plates at 37 °C in 5% CO_2_ atmosphere overnight. The culture medium was replaced with fresh medium to keep the cells growing for 6 days. Colonies were washed with PBS, fixed with 100% methanol and stained with 0.1% crystal violet. The experiments were performed in triplicates. The automated colony counting tool of ImageJ was used for analysis.

### 5.6. RNA Sequencing (RNA-seq)

H1975 and H661 cells were grown in medium supplemented with 10% fetal calf serum without or with addition of 2 mg/mL AAT for 24 h. Afterwards, the total RNA was isolated, and quality was assessed using 1% agarose gels as well as by Agilent 2100 Bioanalyzer using Agilent RNA 6000 Nano Kit. Libraries were prepared in duplicates by using 200 ng RNA of each cell line. TruSeq Stranded mRNA Kit (Illumina) was used for library preparation according to the manufacturer protocol. Briefly, polyA-containing RNA molecules were purified using polyT oligo-attached magnetic beads followed by thermal fragmentation. The cleaved RNA fragments are copied into first strand cDNA using SuperScript II reverse transcriptase and random primers. This was followed by the second strand cDNA synthesis, end repair process, adenylation of 3′ ends and ligation of the adapters. The products were then purified with Ampure XP Beads (Beckman Coulter, Brea, CA) and enriched with 15 cycles of PCR to create the cDNA library. Sequencing was performed at the Genomics service (ISCIII) on a NextSeq 500 sequencer using 75 base read lengths in paired-end mode. The obtained RNA-Seq data was analyzed by the Bioinformatics Facility (ISCIII). First, quality control analysis involving fastQC v0.11.3 Shttp://www.bioinformatics.babraham.ac.uk/projects/fastqc/) was carried out and any adapter sequences and low quality 3′ ends were removed using Trimmomatic v0.36. High-quality reads were then mapped against Hg38 human genome using STAR v2.4.0.1 [64] and mapping quality control was performed using RseQC v2.6.4. Transcriptome prediction and gene/isoform quantification was calculated using HT-seq v0.11.1 [65] based on Hg38 RefSeq reference genes. Finally, differential expression analysis was carried out using GFOLD v1.1.4 [66] in generalized fold change (GFOLD) form. The CummeRbund R package (v2.14.0) was used for quality control and results visualization.

### 5.7. Analysis of RNA-Seq Data from H1975 and H661 Cell Lines

RNA sequencing data in FASTQ.gz form was aligned by STAR v2.4.0.1 [64] aligner to get the output BAM files. The mapped reads were counted by HT-seq v0.11.1 [65] to quantify the expression levels in counts. The differential expression (DE) levels were subsequently ranked by GFOLD v1.1.4 [66] in generalized fold change (GFOLD) form. In the next step, we performed the statistical enrichment test on PANTHER platform [67] to infer the biological functions potentially triggered by the AAT treatment in each cell line. The significant Gene Ontology (GO) terms were defined as those having false discovery rate (FDR) values no greater than 0.05 [68]. All GO enrichment results are provided as Supplementary Table S1.

### 5.8. Statistical Analyses

Data of qPCR, IHC and ELISA analyses were statistically analyzed under REMARK criteria [69] with SPSS 25.0 for Windows (IBM, Ehningen, Germany). The endpoint of the study was overall survival and disease-free survival. Overall survival time was calculated from the date of diagnosis until the last date of contact or death. Disease free-survival was calculated from the date of diagnosis until the first date of disease recurrence (primary tumor or metastases). The cut-offs used for survival analyses were selected using the software tool “Cutoff-Finder” (http://molpath.charite.de/cutoff/index.jsp). Uni- and multivariate survival analyzes were performed using the Cox proportional hazards model. Univariate analysis of survival data was performed according to Kaplan and Meier. The log-rank test was used to test the significance between the groups. The non-parametric, two-tailed Mann-Whitney U test was used to investigate significant differences between the patient groups. The t-test was applied for analyzes of functional cell experiments. Correlation analyzes were performed using the nonparametric Spearman’s rank correlation analyzes. A p-value of less than 0.05 was considered significant. Data were visualized with GraphPad Prism 5 (GraphPad Software, San Diego, CA, USA) and SPSS 25.0 (IBM, Ehningen, Germany).

## 6. Conclusions 

Our data demonstrate that SERPINA1 gene and AAT protein not just reflect inflammatory reaction related to cancer development but play an active role in the pathogenesis of NSCLC. However, further investigations on the mechanisms behind the regulation of *SERPINA1* expression and AAT protein production in tumor cells are needed to complete our understanding on the role of AAT in NSCLC.

## Figures and Tables

**Table 1:** Patient Cohort Characteristics.

Cohort Description		
Parameter	*n*	(%)
Median Age	65 (38–88)	
Total	351	100
Male	243	69
Female	108	31
Histology		
Adeno	205	58
Squamous	146	42
**Therapy**		
OP	205	58
OP/RT	13	4
OP/ChT	97	27
OP/RT/ChT	36	10
**Smoking Status**		
Non-Smoker	37	11
Ex-Smoker	184	52
Smoker	128	36
No data	2	1
**Pathological Stage (7th TNM Edition)**		
IA	37	11
IB	123	35
IIA	24	7
IIB	74	21
IIIA	83	24
IIIB	10	3
**ECOG**		
0	301	86
1	46	13
2	4	1
**BMI**		
<18.5	9	3
18.5 - <25	138	39
25 - <30	122	35
>30	81	23
No data	1	0

OP: operation, RT: radiotherapy, ChT: chemotherapy, ECOG: Eastern Cooperative Oncology Group Performance Status Scale, BMI: Body-Mass-Index.

**Table 2:** Cox Regression Analyses.

**Univariate Analysis (Gene Expression, Overall Survival)**		
**Variable (high vs. low)**	**Significance**	**Hazard Ratio (95% CI)**
*SERPINA1* (tumor)	0.132	0.781 (0.567–1.077)
*SERPINA1* (non-tumor)	0.017	1.508 (1.077–2.112)
*SERPINA1* (tumor vs. non-tumor)	0.495	1.036 (0.935–1.149)
**Multivariate Analysis (Gene Expression, Overall Survival)**		
**Variable**	**Significance**	**Hazard Ratio (95% CI)**
*SERPINA1* (tumor, high vs. low)	0.117	0.752 (0.526–1.074)
*SERPINA1* (non-tumor, high vs. low)	0.028	1.540 (1.047–2.265)
*SERPINA1* (tumor vs. non-tumor, high vs. low)	0.316	1.050 (0.954–1.155)
Sex (female vs. male)	0.105	0.727 (0.495–1.069)
pathological stage (7th edition)	<0.0001	1.069 (1.046–1.093)
Histology (ADC vs. SQCC)	0.389	1.175 (0.814–1.697)
age	<0.0001	1.038 (1.018–1.057)
**Univariate Analysis (IHC, Overall Survival)**		
**Variable**	**Significance**	**Hazard Ratio (95% CI)**
AAT (high vs. low)	0.219	1.454 (0.800–2.641)
**Univariate Analysis (Blood, Overall Survival)**		
**Variable**	**Significance**	**Hazard Ratio (95% CI)**
AAT (high vs. low)	0.035	1.674 (1.036–2.704)
**Multivariate Analysis (Blood, Overall Survival)**		
**Variable**	**Significance**	**Hazard Ratio (95% CI)**
AAT (high vs. low)	0.009	1.938 (1.176–3.194)
Sex (female vs. male)	0.259	0.725 (0.415–1.267)
pathological stage (7th edition)	<0.0001	2.211 (1.552–3.149)
Histology (ADC vs. SQCC)	0.731	0.918 (0.564–1.494)
age	0.220	1.017 (0.990–1.045)

CI: confidence interval, AAT: *α*1-antitrypsin; ADC: adenocarcinoma, SQCC: squamous cell carcinoma, IHC: Immunohistochemistry.

**Table 3:** S*ERPINA1*-related Differentially Expressed Genes in H1975 and H661 Cells.

**H1975**			
**Gene Symbol**	**Description to the Gene**	**Regulation Direction**	**Annotated GO Terms in the Analysis**
*APOE*	Apolipoprotein E	Upregulated	inflammatory response, regulation of inflammatory response
*APP*	Amyloid Beta Precursor Protein	Upregulated	inflammatory response, regulation of inflammatory response, positive regulation of apoptotic process, regulation of inflammatory response
*C3*	Complement C3	Upregulated	inflammatory response, regulation of inflammatory response
*CLU*	Clusterin	Upregulated	apoptotic process, positive regulation of apoptotic process
*CTSC*	Cathepsin C	Upregulated	apoptotic process, positive regulation of apoptotic process
*CYR61*	Cysteine-rich angiogenic inducer 61	Upregulated	apoptotic process, positive regulation of apoptotic process
*EVA1A*	Eva-1 Homolog A, Regulator Of Programmed Cell Death	Upregulated	apoptotic process, positive regulation of apoptotic process
*IGFBP3*	Insulin Like Growth Factor Binding Protein 3	Upregulated	apoptotic process, positive regulation of apoptotic process
*IL6*	Interleukin 6	Upregulated	inflammatory response, apoptotic process, positive regulation of apoptotic process, regulation of inflammatory response
*LGALS1*	Galectin 1	Upregulated	apoptotic process, positive regulation of apoptotic process
*PCSK9*	Proprotein Convertase Subtilisin/Kexin Type 9	Downregulated	apoptotic process, positive regulation of apoptotic process
*PROC*	Protein C, Inactivator Of Coagulation Factors Va And VIIIa	Upregulated	inflammatory response, regulation of inflammatory response
*SERPINE1*	Serpin Family E Member 1	Upregulated	inflammatory response, regulation of inflammatory response
*SHISA5*	Shisa Family Member 5	Upregulated	apoptotic process, positive regulation of apoptotic process
*TGFB1*	Transforming Growth Factor Beta 1	Upregulated	apoptotic process, positive regulation of apoptotic process
*TGFB2*	Transforming Growth Factor Beta 2	Upregulated	apoptotic process, positive regulation of apoptotic process
*TMSB4X*	Thymosin Beta 4 X-Linked	Upregulated	inflammatory response, regulation of inflammatory response
H661			
**Gene Symbol**	**Description to the Gene**	**Regulation Direction**	**Annotated GO Terms in the Analysis**
*APOE*	Apolipoprotein E	Upregulated	inflammatory response, regulation of inflammatory response
*APP*	Amyloid Beta Precursor Protein	Upregulated	inflammatory response, regulation of inflammatory response
*CLU*	Clusterin	Upregulated	cell population proliferation, apoptotic process
*CYR61*	Cysteine-rich angiogenic inducer 61	Downregulated	apoptotic process
*IGF1*	Insulin Like Growth Factor 1	Upregulated	inflammatory response, cell population proliferation
*LGALS1*	Galectin 1	Upregulated	apoptotic process
*SERPINE1*	Serpin Family E Member 1	Upregulated	inflammatory response, regulation of inflammatory response
*SHISA5*	Shisa Family Member 5	Upregulated	apoptotic process
*TGFB1*	Transforming Growth Factor Beta 1	Downregulated	cell population proliferation, apoptotic process
*TGFB2*	Transforming Growth Factor Beta 2	Downregulated	cell population proliferation, apoptotic process

GO: gene ontology.

## Supplementary Materials

The following are available online at https://www.mdpi.com/2072-6694/11/9/1306/s1, Figure S1: Hazard ratios and further survival plots based on *SERPINA1* gene expression; Figure S2: IHC controls, hazard ratios and examples of different AAT protein expression patterns; Figure S3: Correlation of AAT serum concentration with gene expression and lung tissue, hazard ratio and further survival plots based on AAT serum concentrations; Figure S4: CRP and neutrophil to lymphocyte ratio (NLR) hazard ratios used for survival analyses; Figure S5: Expression of *SERPINA1* in NSCLC cell lines and annexin V assay; Figure S6: Controls for siRNA knockdown and overexpression of *SERPINA1* in H1975 and H661 cells. Table S1: Gene ontology enrichment results for H1975 and H661 cells.
