# Supplementary material for: Clinical Significance of SERPINA1 Gene and Its Encoded Alpha1-Antitrypsin Protein in NSCLC
Source: Cancers (Basel). 2019 Sep 4;11(9):1306. doi: 10.3390/cancers11091306 (PMC6770941; doi:10.3390/cancers11091306)
Supplement: Supplementary file 1 [file cancers-11-01306-s001.zip › cancers-580565-suppl-figure.docx]

**Supplymentary:**


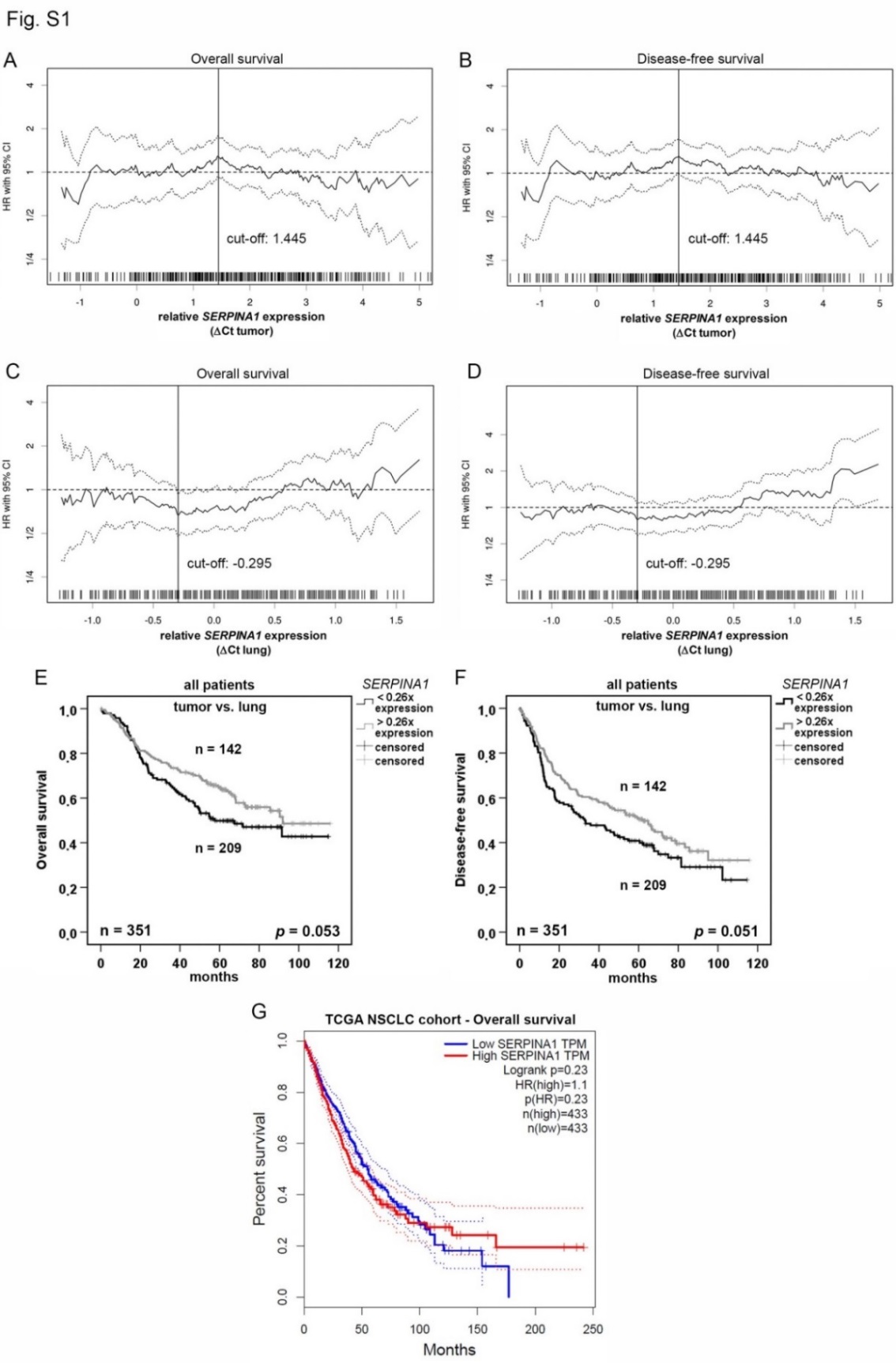


**Figure S1.** Hazard ratios and further survival plots based on *SERPINA1* gene expression**. (A–D)**. The program cut-off finder (http://molpath.charite.de/cutoff/) was used to define the values separating the groups. Dotted lines indicate a hazard ratio of 1. Vertical lines indicate the value used for survival analyses to separate between the two groups. (**E–G)**, Kaplan-Meier curves based on the expression ratio of *SERPINA1* in tumor and paired lung tissue and in TCGA NSCLC cohort. *p* < 0.05 was considered significant.


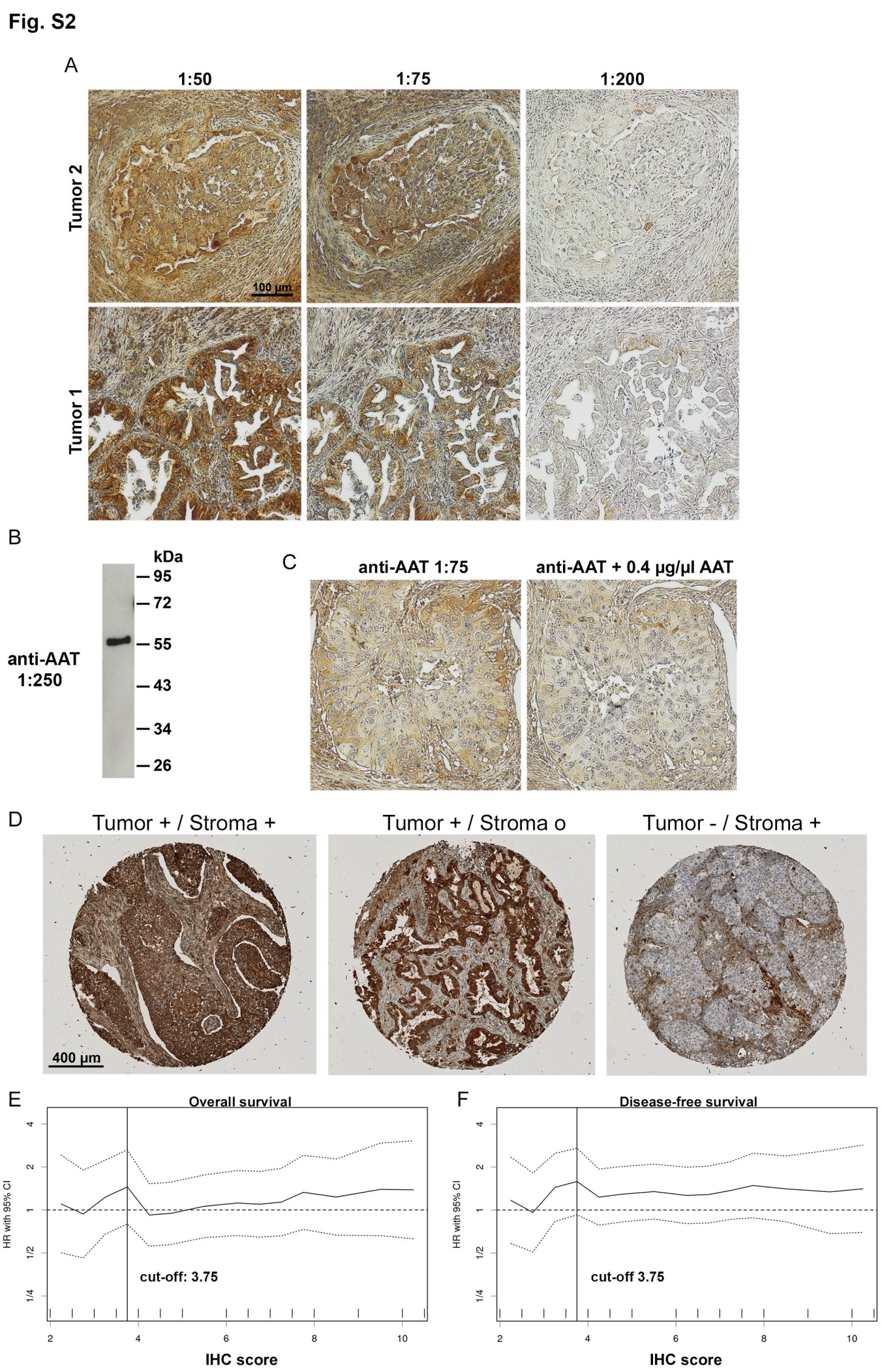


**
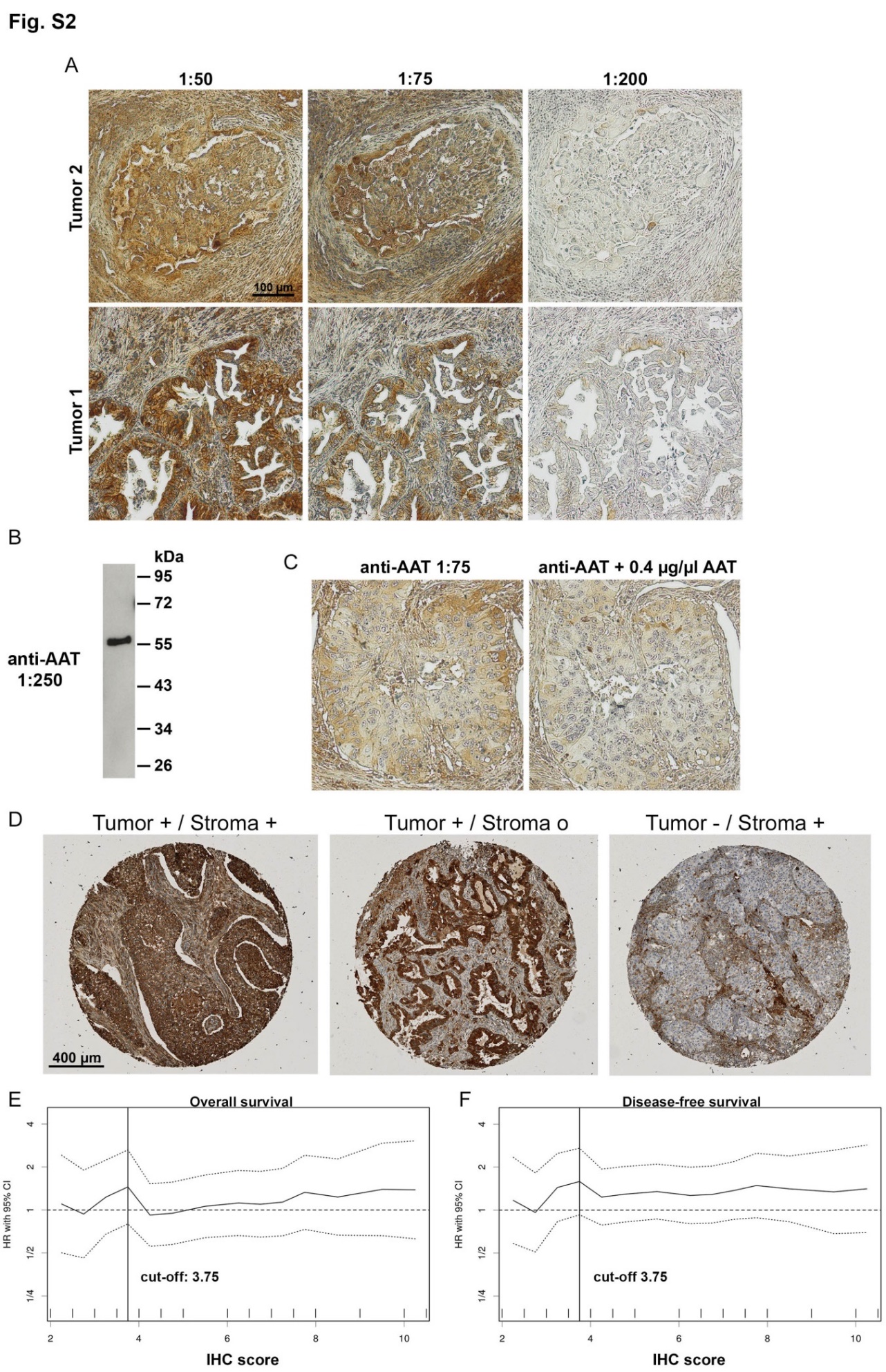
 Figure S2.** IHC controls, hazard ratios and examples of different AAT protein expression patterns. **(A**) Staining pattern and intensities of AAT in two tumor samples using different dilutions of the monoclonal anti-AAT antibody. (**B)** Validation of the antibody using 100 ng of purified AAT protein as positive control. (**C)** Positive and negative control of AAT staining. FFPE slides were incubated with the monoclonal antibody either alone or with the antibody pre-incubated with AAT for 1 h at room temperature before staining procedure. (**D)** Examples of AAT tumor and tumor stroma staining patterns. + = high expression, o = medium expression, - = low expression of AAT. (**E)** and (**F)** The program cut-off finder (http://molpath.charite.de/cutoff/) was used to define the values separating the groups. Dotted lines indicate a hazard ratio of 1. Vertical lines indicate the value used for survival analyses to separate between the two groups.


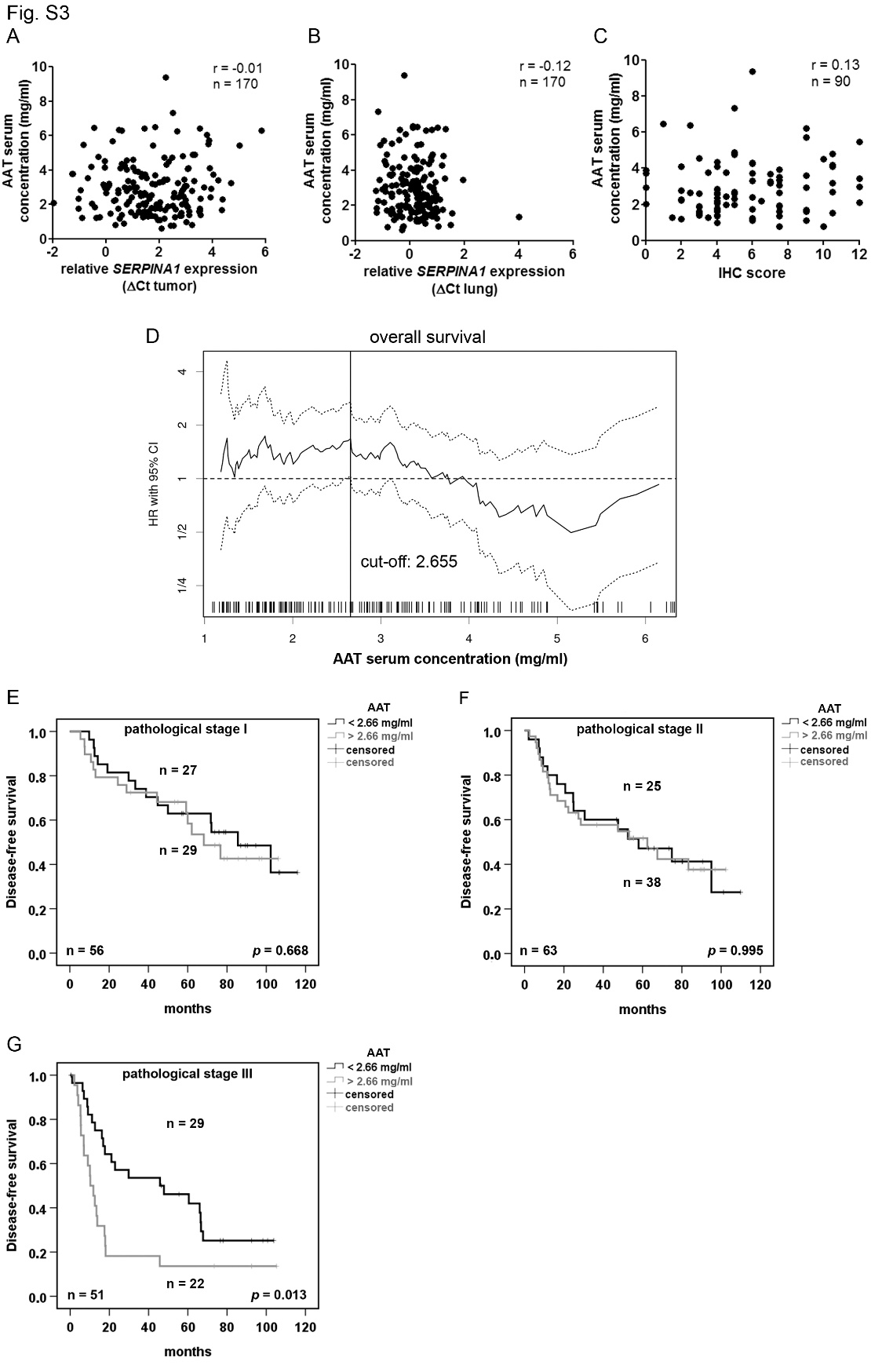


**Figure S3.** Correlation of AAT serum concentration with gene expression and lung tissue, hazard ratio and further survival plots based on AAT serum concentrations. (**A)** Correlation analyses of AAT serum concentrations and relative S*ERPINA1* gene expression of the same patients in normal lung. (**B)** hazard ratio plot was created using the program cut-off finder (http://molpath.charite.de/cutoff/). Dotted line indicates a hazard ratio of 1. Vertical line indicates the value used for survival analyses to separate between the two groups. (**C-G)** Kaplan-Meier curves of gender-specific and stage-specific survival prognoses based on AAT serum concentration. H, AAT serum concentrations depending on the pathological tumor stage of the patients. *p* < 0.05 was considered significant.


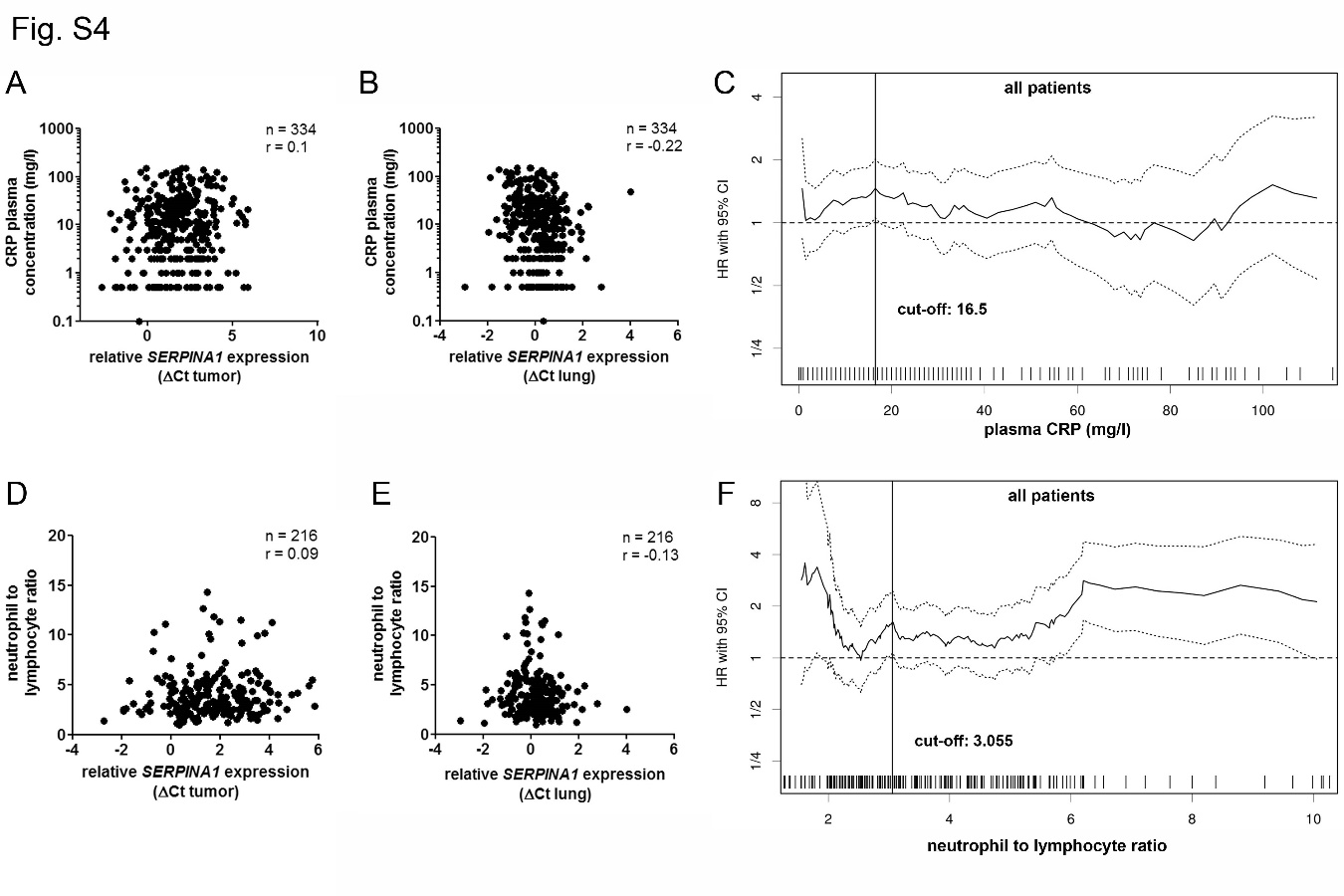


**
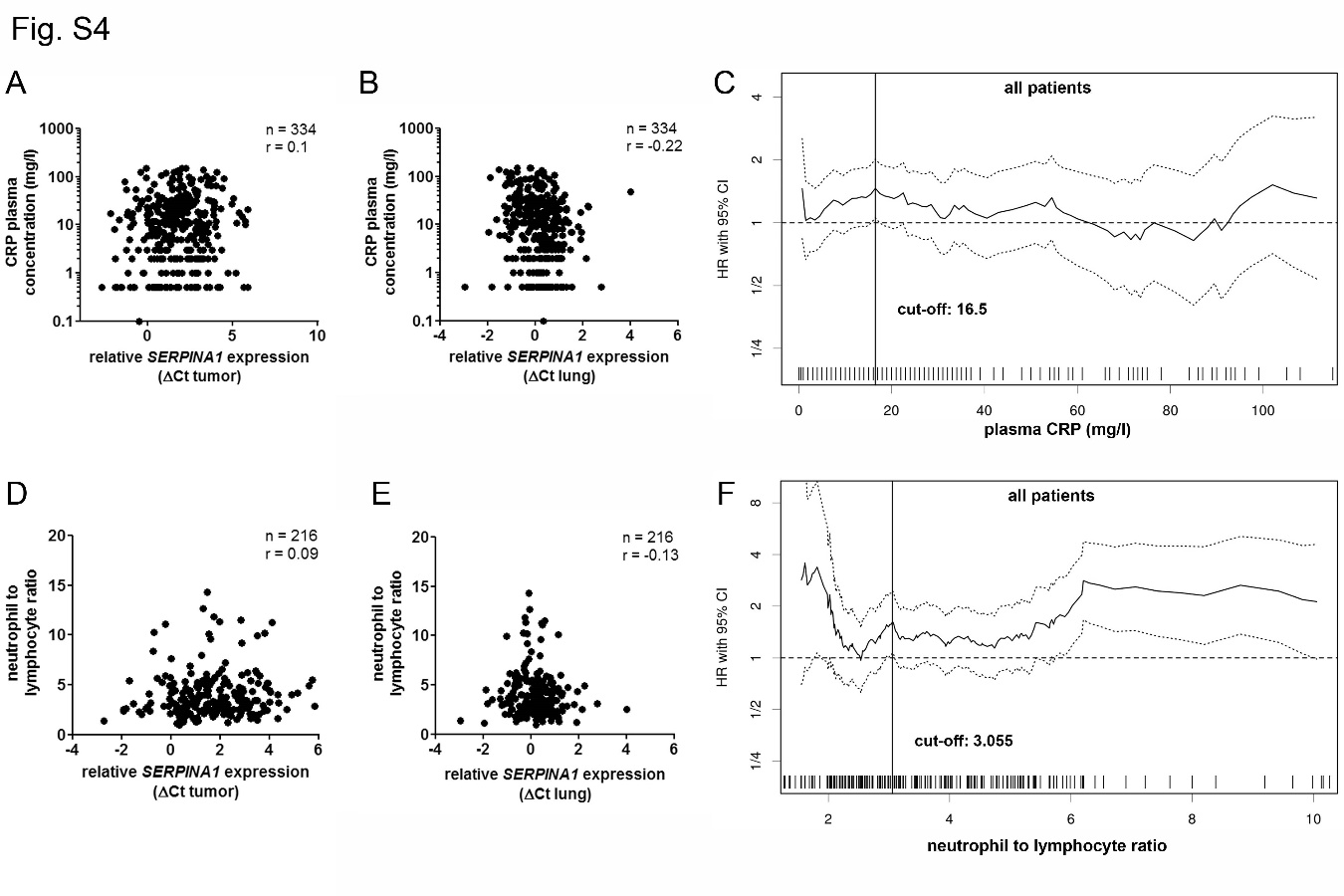
Figure S4.** CRP and neutrophil to lymphocyte ratio (NLR) hazard ratios used for survival analyses. **(A)** and (**B)** Correlation analyses of CRP plasma concentration and *SERPINA* gene expression in tumor and corresponding normal lung tissue. (**C)** and (**F**) Hazard ratio plots were created using the program cut-off finder (http://molpath.charite.de/cutoff/). Dotted line indicates a hazard ratio of 1. Vertical line indicates the value used for survival analyses to separate between the two groups. (**D)** and (**E)** Correlation analyses of neutrophil to lymphocyte ratio and *SERPINA* gene expression in tumor and corresponding normal lung tissue.


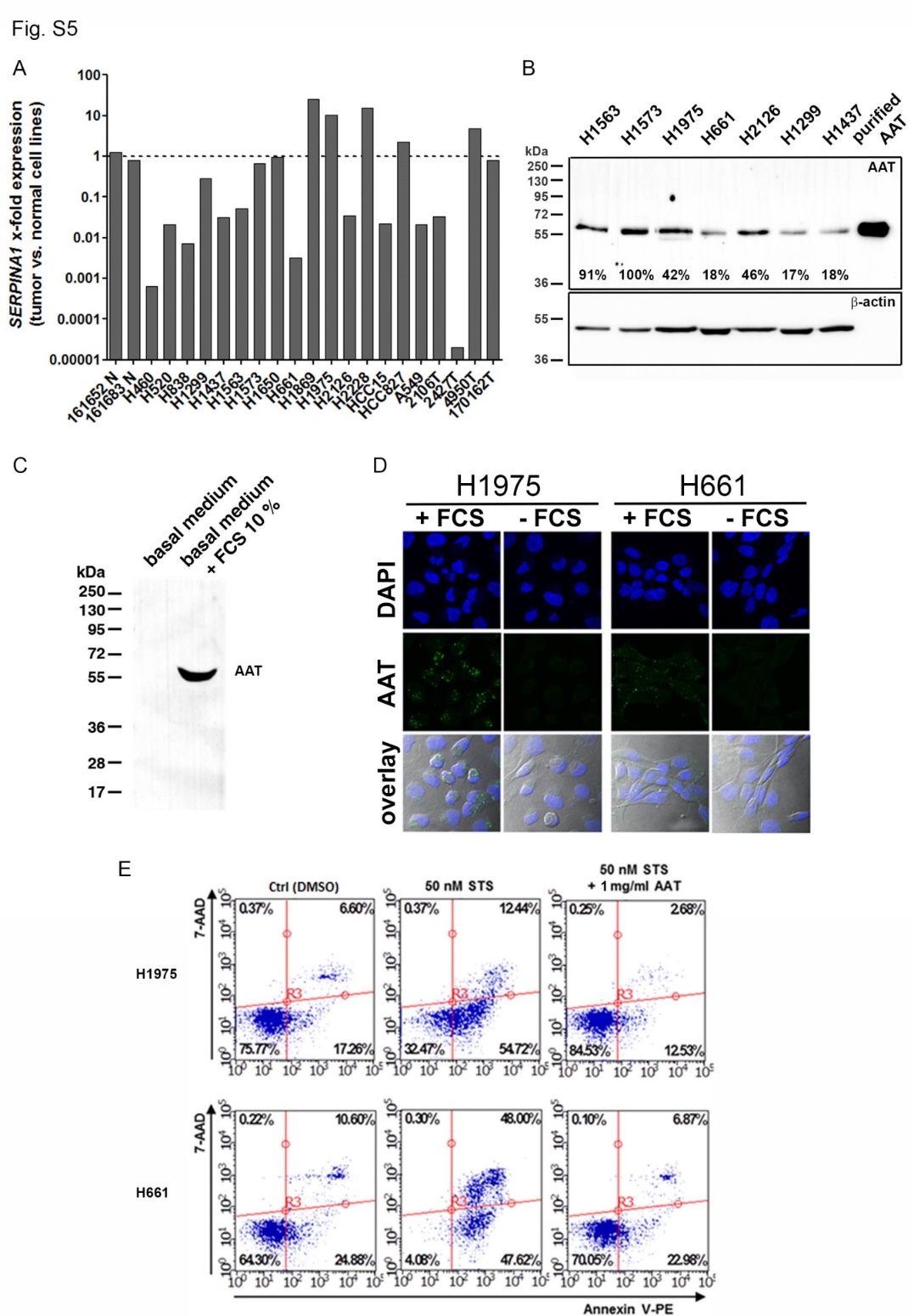
­

**Figure S5.** Expression of *SERPINA1* in NSCLC cell lines and annexin V assay. (**A**) *SERPINA1* expression levels of NSCLC cell lines compared to normal primary cell lines using qPCR. (**B**) AAT expression levels in selected NSCLC cell lines. Cells were incubated in serum-free medium for 18 h prior ty cell lysis. AAT was stained with a polyclonal rabbit anti-AAT antibody. Percentages indicate densitometric determination of AAT levels normalized to *β*-actin levels. AAT level of H1573 was set to 100%. (**C)** Western blot showing that anti-AAT antibody reacts specifically with serum AAT. (**D)** Confocal immunofluorescence staining images of AAT protein (green) in H1975 and H661 cells cultured for 18 hours in serum-containing medium. AAT was stained using a monoclonal anti-AAT antibody. Nuclei were defined by DAPI (blue). Scale bar: 30 µm. (**E)** Western blot showing that AAT in FCS is stained by rabbit polyclonal antibody. D. Flow cytometric analysis of apoptosis in native H1975 and H661 cells incubated with staurosporine (50 nM) with/without AAT (1 mg/mL) measured using phycoerythrin (PE)-labeled annexin V and 7-AAD. Cells are classified as “viable” (bottom left), “apoptotic” (bottom right and top right), or “necrotic” (top left).


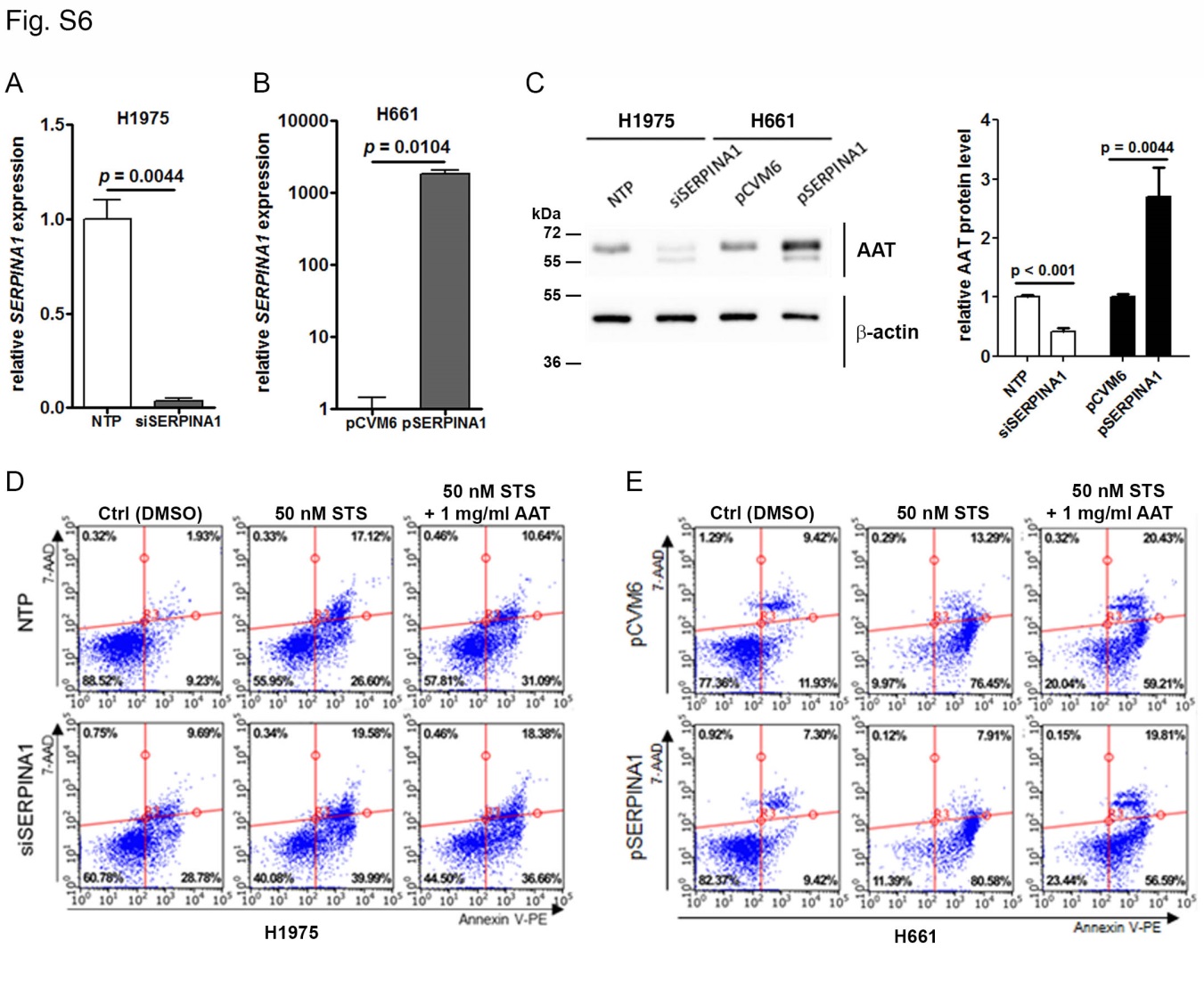


**
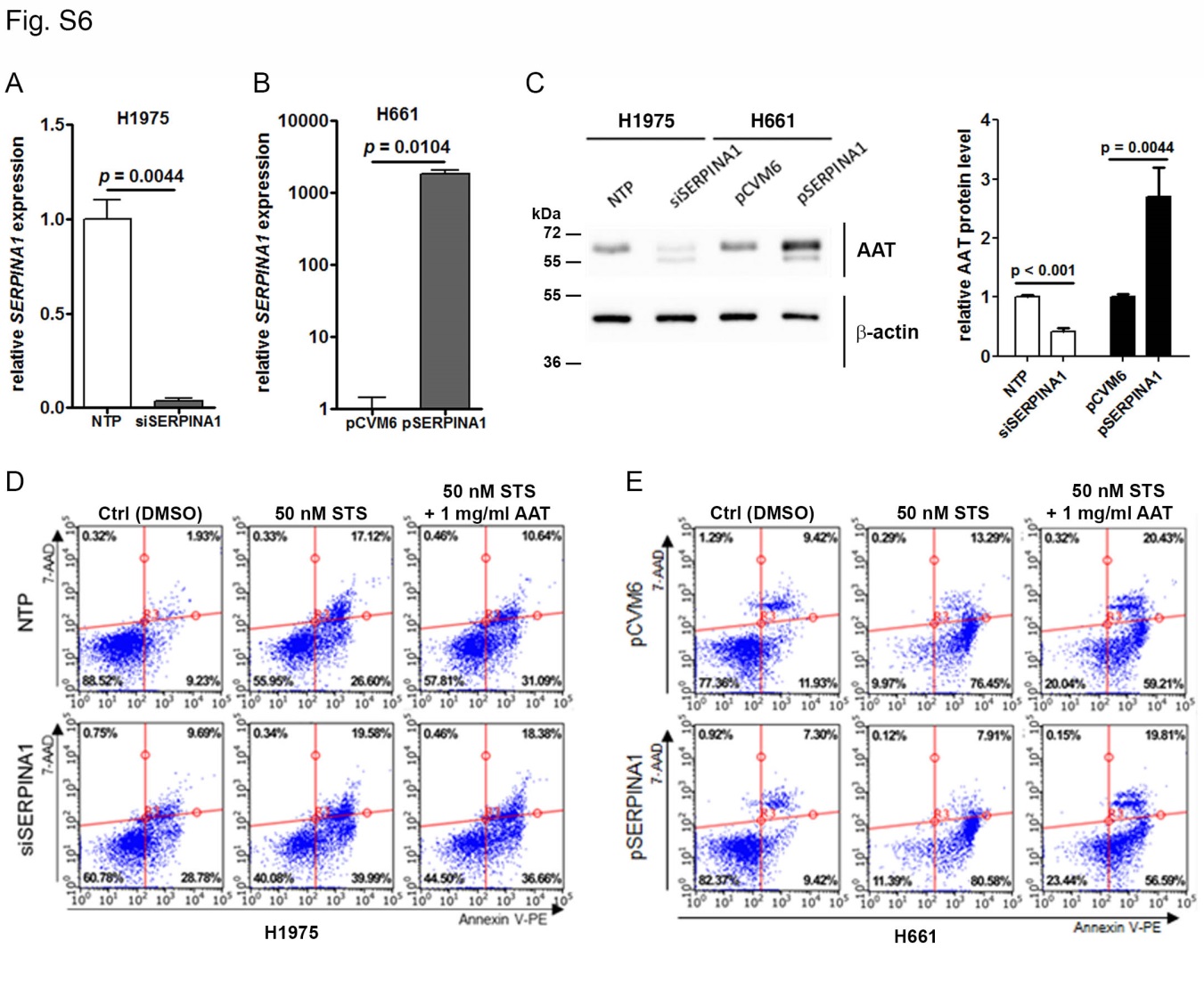
Figure S6.** Controls for siRNA knockdown and overexpression of *SERPINA1* in H1975 and H661 cells. (**A)** Proof of *SERPINA1* knockdown efficiency in H1975. (**B)** Efficiency of *SERPINA1* overexpression in H661. NTP: non-target-pool siRNA and pCVM6 as control vector. Each bar represents mean ± SD from three independent experiments. (**C)** Representative blot (out of 3 experiments) and densitometry evaluation after silencing or overexpression of *SERPINA1*. Beta (ß)-actin is used as loading control. (**D)** and (**E**) Flow cytometric analyses of apoptosis after *SERPINA1* knockdown in H1975 and *SERPINA1* overexpressing H661 cells. Cells were incubated with staurosporine (50 nM) with/without AAT (1 mg/mL) followed by measuring phycoerythrin (PE)-labeled annexin V and 7-AAD. Cells are classified as “viable” (bottom left), “apoptotic” (bottom right and top right), or “necrotic” (top left).
