# Supplementary material for: Clinical Significance of SERPINA1 Gene and Its Encoded Alpha1-Antitrypsin Protein in NSCLC
Source: Cancers (Basel). 2019 Sep 4;11(9):1306. doi: 10.3390/cancers11091306 (PMC6770941; doi:10.3390/cancers11091306)
Supplement: Supplementary file 1 [file cancers-11-01306-s001.zip › cancers-580565-suppl-table.docx]

**Supplementary Table 1**

| **Analysis Type:** | **PANTHER Enrichment Test (release 20190701)** |  | |  |
| --- | --- | --- | --- | --- |
| **Annotation Version and Release Date:** | **GO Ontology database Released 2019-02-02** |  | |  |
| **Analyzed List:** | **H1975_GFold.txt (Homo sapiens)** | |  |  |
| **Correction:** | **FDR** |  |  |  |
| **GO biological process complete** | **number** | **overUnder** | **pvalue** | **fdr** |
| immune response (GO:0006955) | 1529 | + | 0 | 0 |
| defense response (GO:0006952) | 1210 | + | 0 | 0 |
| response to cytokine (GO:0034097) | 1068 | + | 0 | 0 |
| type I interferon signaling pathway (GO:0060337) | 66 | + | 0 | 0 |
| mitotic cell cycle process (GO:1903047) | 575 | - | 0 | 0 |
| cytokine-mediated signaling pathway (GO:0019221) | 655 | + | 0 | 0 |
| immune system process (GO:0002376) | 2430 | + | 0 | 0 |
| SRP-dependent cotranslational protein targeting to membrane (GO:0006614) | 96 | + | 0 | 0 |
| cotranslational protein targeting to membrane (GO:0006613) | 100 | + | 0 | 0 |
| protein targeting to membrane (GO:0006612) | 158 | + | 0 | 0 |
| immune effector process (GO:0002252) | 942 | + | 0 | 0 |
| translational initiation (GO:0006413) | 144 | + | 0 | 0 |
| translation (GO:0006412) | 381 | + | 0 | 0 |
| mRNA catabolic process (GO:0006402) | 208 | + | 0 | 0 |
| protein localization to endoplasmic reticulum (GO:0070972) | 136 | + | 0 | 0 |
| cellular response to type I interferon (GO:0071357) | 66 | + | 0 | 0 |
| cellular response to cytokine stimulus (GO:0071345) | 981 | + | 0 | 0 |
| cellular response to organic substance (GO:0071310) | 2283 | + | 0 | 0 |
| protein targeting to ER (GO:0045047) | 109 | + | 0 | 0 |
| response to other organism (GO:0051707) | 853 | + | 0 | 0 |
| cellular response to chemical stimulus (GO:0070887) | 2790 | + | 0 | 0 |
| nuclear-transcribed mRNA catabolic process (GO:0000956) | 192 | + | 0 | 0 |
| establishment of protein localization to membrane (GO:0090150) | 258 | + | 0 | 0 |
| response to biotic stimulus (GO:0009607) | 881 | + | 0 | 0 |
| mitotic cell cycle (GO:0000278) | 670 | - | 0 | 0 |
| nuclear-transcribed mRNA catabolic process, nonsense-mediated decay (GO:0000184) | 119 | + | 0 | 0 |
| response to external biotic stimulus (GO:0043207) | 855 | + | 0 | 0 |
| peptide biosynthetic process (GO:0043043) | 403 | + | 0 | 0 |
| mRNA metabolic process (GO:0016071) | 662 | + | 0 | 0 |
| response to type I interferon (GO:0034340) | 71 | + | 0 | 0 |
| establishment of protein localization to endoplasmic reticulum (GO:0072599) | 113 | + | 0 | 0 |
| RNA catabolic process (GO:0006401) | 236 | + | 0 | 0 |
| response to virus (GO:0009615) | 279 | + | 0 | 0 |
| gene expression (GO:0010467) | 1940 | + | 0 | 0 |
| cell cycle process (GO:0022402) | 946 | - | 0 | 0 |
| innate immune response (GO:0045087) | 648 | + | 0 | 0 |
| ribonucleoprotein complex biogenesis (GO:0022613) | 428 | + | 0 | 0 |
| response to organic substance (GO:0010033) | 2889 | + | 0 | 0 |
| response to interferon-gamma (GO:0034341) | 181 | + | 0 | 0 |
| RNA metabolic process (GO:0016070) | 1602 | + | 0 | 0 |
| ribosome biogenesis (GO:0042254) | 279 | + | 0 | 0 |
| cell cycle (GO:0007049) | 1298 | - | 0 | 0 |
| peptide metabolic process (GO:0006518) | 520 | + | 0 | 0 |
| symbiont process (GO:0044403) | 757 | + | 0 | 0 |
| viral process (GO:0016032) | 681 | + | 0 | 0 |
| multi-organism process (GO:0051704) | 2387 | + | 0 | 0 |
| interspecies interaction between organisms (GO:0044419) | 800 | + | 0 | 0.000000000117 |
| nucleobase-containing compound catabolic process (GO:0034655) | 360 | + | 0 | 0.000000000137 |
| positive regulation of response to stimulus (GO:0048584) | 2243 | + | 0 | 0.000000000151 |
| positive regulation of immune system process (GO:0002684) | 988 | + | 0 | 0.000000000177 |
| defense response to virus (GO:0051607) | 193 | + | 0 | 0.000000000175 |
| cell surface receptor signaling pathway (GO:0007166) | 2291 | + | 0 | 0.00000000019 |
| RNA processing (GO:0006396) | 837 | + | 0 | 0.000000000192 |
| response to lipopolysaccharide (GO:0032496) | 309 | + | 0 | 0.000000000193 |
| cellular response to lipopolysaccharide (GO:0071222) | 182 | + | 0 | 0.000000000225 |
| programmed cell death (GO:0012501) | 1030 | + | 0 | 0.00000000024 |
| regulation of immune system process (GO:0002682) | 1481 | + | 0 | 0.000000000248 |
| cell division (GO:0051301) | 480 | - | 0 | 0.000000000264 |
| cell activation (GO:0001775) | 1045 | + | 0 | 0.000000000298 |
| cell death (GO:0008219) | 1064 | + | 0 | 0.000000000371 |
| rRNA processing (GO:0006364) | 201 | + | 0 | 0.000000000385 |
| regulation of chromosome segregation (GO:0051983) | 105 | - | 0 | 0.000000000384 |
| regulation of cytokine production (GO:0001817) | 679 | + | 0 | 0.000000000383 |
| response to molecule of bacterial origin (GO:0002237) | 322 | + | 0 | 0.000000000407 |
| regulation of response to stress (GO:0080134) | 1436 | + | 0 | 0.000000000462 |
| regulation of defense response (GO:0031347) | 735 | + | 0 | 0.000000000929 |
| nuclear division (GO:0000280) | 270 | - | 0 | 0.00000000109 |
| chromosome segregation (GO:0007059) | 260 | - | 0 | 0.00000000123 |
| regulation of immune response (GO:0050776) | 981 | + | 0 | 0.00000000124 |
| nuclear chromosome segregation (GO:0098813) | 210 | - | 0 | 0.00000000125 |
| defense response to other organism (GO:0098542) | 422 | + | 0 | 0.00000000124 |
| myeloid leukocyte activation (GO:0002274) | 572 | + | 0 | 0.0000000013 |
| cellular nitrogen compound catabolic process (GO:0044270) | 409 | + | 0 | 0.00000000132 |
| viral transcription (GO:0019083) | 115 | + | 0 | 0.00000000139 |
| heterocycle catabolic process (GO:0046700) | 408 | + | 0 | 0.00000000158 |
| cellular response to molecule of bacterial origin (GO:0071219) | 189 | + | 0 | 0.00000000168 |
| regulation of proteolysis (GO:0030162) | 710 | + | 0 | 0.00000000197 |
| response to bacterium (GO:0009617) | 580 | + | 0 | 0.00000000222 |
| viral gene expression (GO:0019080) | 129 | + | 0 | 0.00000000227 |
| response to external stimulus (GO:0009605) | 1959 | + | 0 | 0.00000000235 |
| leukocyte activation (GO:0045321) | 901 | + | 0 | 0.00000000264 |
| rRNA metabolic process (GO:0016072) | 211 | + | 0 | 0.00000000267 |
| regulation of mitotic sister chromatid separation (GO:0010965) | 60 | - | 0 | 0.00000000295 |
| regulation of cell death (GO:0010941) | 1661 | + | 0 | 0.00000000344 |
| protein targeting (GO:0006605) | 348 | + | 0 | 0.00000000372 |
| myeloid leukocyte mediated immunity (GO:0002444) | 514 | + | 0 | 0.00000000386 |
| DNA replication (GO:0006260) | 211 | - | 0 | 0.00000000432 |
| regulated exocytosis (GO:0045055) | 693 | + | 0 | 0.00000000471 |
| regulation of apoptotic process (GO:0042981) | 1520 | + | 0 | 0.00000000477 |
| regulation of cysteine-type endopeptidase activity (GO:2000116) | 235 | + | 0 | 0.00000000473 |
| chromosome organization (GO:0051276) | 1014 | - | 0 | 0.0000000047 |
| positive regulation of proteolysis (GO:0045862) | 349 | + | 0 | 0.00000000605 |
| regulation of chromosome separation (GO:1905818) | 65 | - | 0 | 0.00000000681 |
| organelle fission (GO:0048285) | 296 | - | 0 | 0.00000000819 |
| regulation of mitotic sister chromatid segregation (GO:0033047) | 71 | - | 0 | 0.00000000867 |
| cytoplasmic translation (GO:0002181) | 66 | + | 0 | 0.00000000873 |
| cellular response to unfolded protein (GO:0034620) | 120 | + | 0 | 0.0000000093 |
| regulation of protein metabolic process (GO:0051246) | 2718 | + | 0 | 0.00000000932 |
| negative regulation of metabolic process (GO:0009892) | 2815 | + | 0 | 0.00000000989 |
| regulation of cysteine-type endopeptidase activity involved in apoptotic process (GO:0043281) | 212 | + | 0 | 0.00000000993 |
| regulation of sister chromatid segregation (GO:0033045) | 83 | - | 0 | 0.00000000987 |
| leukocyte degranulation (GO:0043299) | 504 | + | 0 | 0.00000000987 |
| microtubule cytoskeleton organization (GO:0000226) | 450 | - | 0 | 0.00000000989 |
| granulocyte activation (GO:0036230) | 500 | + | 0 | 0.0000000106 |
| myeloid cell activation involved in immune response (GO:0002275) | 517 | + | 0 | 0.0000000111 |
| regulation of I-kappaB kinase/NF-kappaB signaling (GO:0043122) | 230 | + | 0 | 0.0000000119 |
| negative regulation of macromolecule metabolic process (GO:0010605) | 2571 | + | 0 | 0.0000000136 |
| regulation of programmed cell death (GO:0043067) | 1539 | + | 0.000000000109 | 0.0000000159 |
| positive regulation of signal transduction (GO:0009967) | 1624 | + | 0.000000000111 | 0.0000000161 |
| cellular response to interferon-gamma (GO:0071346) | 160 | + | 0.000000000117 | 0.0000000167 |
| positive regulation of signaling (GO:0023056) | 1786 | + | 0.000000000129 | 0.0000000184 |
| negative regulation of response to stimulus (GO:0048585) | 1572 | + | 0.00000000013 | 0.0000000184 |
| positive regulation of cell communication (GO:0010647) | 1778 | + | 0.000000000138 | 0.0000000193 |
| neutrophil activation (GO:0042119) | 495 | + | 0.000000000138 | 0.0000000192 |
| neutrophil mediated immunity (GO:0002446) | 493 | + | 0.000000000138 | 0.000000019 |
| regulation of cellular protein metabolic process (GO:0032268) | 2550 | + | 0.000000000144 | 0.0000000196 |
| neutrophil degranulation (GO:0043312) | 482 | + | 0.000000000159 | 0.0000000215 |
| regulation of multi-organism process (GO:0043900) | 401 | + | 0.000000000173 | 0.0000000232 |
| response to endoplasmic reticulum stress (GO:0034976) | 240 | + | 0.000000000175 | 0.0000000232 |
| mitotic cell cycle phase transition (GO:0044772) | 260 | - | 0.000000000179 | 0.0000000236 |
| microtubule-based process (GO:0007017) | 636 | - | 0.000000000193 | 0.0000000253 |
| cilium organization (GO:0044782) | 345 | - | 0.000000000203 | 0.0000000263 |
| amide biosynthetic process (GO:0043604) | 519 | + | 0.000000000204 | 0.0000000262 |
| negative regulation of gene expression (GO:0010629) | 1668 | + | 0.000000000212 | 0.0000000271 |
| organic cyclic compound catabolic process (GO:1901361) | 458 | + | 0.000000000219 | 0.0000000277 |
| apoptotic process (GO:0006915) | 900 | + | 0.00000000022 | 0.0000000275 |
| negative regulation of biological process (GO:0048519) | 5151 | + | 0.000000000228 | 0.0000000284 |
| neutrophil activation involved in immune response (GO:0002283) | 486 | + | 0.000000000229 | 0.0000000282 |
| aromatic compound catabolic process (GO:0019439) | 425 | + | 0.000000000238 | 0.0000000292 |
| DNA metabolic process (GO:0006259) | 713 | - | 0.000000000257 | 0.0000000313 |
| positive regulation of DNA-binding transcription factor activity (GO:0051091) | 264 | + | 0.000000000283 | 0.0000000342 |
| positive regulation of intracellular signal transduction (GO:1902533) | 994 | + | 0.000000000348 | 0.0000000417 |
| protein localization to membrane (GO:0072657) | 475 | + | 0.000000000354 | 0.0000000421 |
| response to stress (GO:0006950) | 3330 | + | 0.000000000477 | 0.0000000563 |
| regulation of mitotic metaphase/anaphase transition (GO:0030071) | 54 | - | 0.000000000502 | 0.0000000588 |
| ribosomal small subunit biogenesis (GO:0042274) | 64 | + | 0.000000000516 | 0.00000006 |
| regulation of endopeptidase activity (GO:0052548) | 407 | + | 0.000000000532 | 0.0000000614 |
| ciliary basal body-plasma membrane docking (GO:0097711) | 95 | - | 0.00000000057 | 0.0000000653 |
| positive regulation of cell death (GO:0010942) | 683 | + | 0.000000000599 | 0.0000000681 |
| cilium assembly (GO:0060271) | 328 | - | 0.000000000727 | 0.0000000821 |
| regulation of viral process (GO:0050792) | 200 | + | 0.000000000775 | 0.0000000869 |
| regulation of metaphase/anaphase transition of cell cycle (GO:1902099) | 56 | - | 0.000000000779 | 0.0000000868 |
| cellular response to topologically incorrect protein (GO:0035967) | 141 | + | 0.000000000809 | 0.0000000894 |
| cell cycle phase transition (GO:0044770) | 268 | - | 0.000000000932 | 0.000000102 |
| regulation of response to stimulus (GO:0048583) | 4199 | + | 0.000000000939 | 0.000000102 |
| response to chemical (GO:0042221) | 4288 | + | 0.000000000966 | 0.000000105 |
| cellular response to biotic stimulus (GO:0071216) | 211 | + | 0.000000000976 | 0.000000105 |
| positive regulation of apoptotic process (GO:0043065) | 629 | + | 0.00000000102 | 0.000000109 |
| regulation of peptidase activity (GO:0052547) | 433 | + | 0.00000000112 | 0.000000119 |
| regulation of apoptotic signaling pathway (GO:2001233) | 398 | + | 0.0000000012 | 0.000000127 |
| ribosome assembly (GO:0042255) | 62 | + | 0.00000000124 | 0.00000013 |
| DNA repair (GO:0006281) | 484 | - | 0.00000000141 | 0.000000147 |
| vesicle-mediated transport (GO:0016192) | 1766 | + | 0.00000000157 | 0.000000162 |
| positive regulation of programmed cell death (GO:0043068) | 633 | + | 0.00000000174 | 0.000000178 |
| positive regulation of immune response (GO:0050778) | 703 | + | 0.00000000177 | 0.00000018 |
| regulation of viral life cycle (GO:1903900) | 142 | + | 0.00000000198 | 0.0000002 |
| regulation of symbiosis, encompassing mutualism through parasitism (GO:0043903) | 229 | + | 0.000000002 | 0.000000202 |
| positive regulation of endopeptidase activity (GO:0010950) | 167 | + | 0.00000000201 | 0.000000201 |
| regulation of response to cytokine stimulus (GO:0060759) | 167 | + | 0.0000000022 | 0.000000219 |
| leukocyte mediated immunity (GO:0002443) | 633 | + | 0.00000000287 | 0.000000284 |
| secretion by cell (GO:0032940) | 986 | + | 0.00000000384 | 0.000000377 |
| meiotic cell cycle (GO:0051321) | 219 | - | 0.0000000039 | 0.000000381 |
| centrosome cycle (GO:0007098) | 83 | - | 0.00000000403 | 0.000000391 |
| exocytosis (GO:0006887) | 779 | + | 0.00000000405 | 0.000000391 |
| DNA-dependent DNA replication (GO:0006261) | 117 | - | 0.00000000468 | 0.000000449 |
| endoplasmic reticulum unfolded protein response (GO:0030968) | 99 | + | 0.00000000475 | 0.000000452 |
| response to tumor necrosis factor (GO:0034612) | 261 | + | 0.00000000555 | 0.000000525 |
| regulation of mRNA metabolic process (GO:1903311) | 323 | + | 0.00000000587 | 0.000000552 |
| spindle organization (GO:0007051) | 135 | - | 0.00000000632 | 0.000000591 |
| positive regulation of NF-kappaB transcription factor activity (GO:0051092) | 151 | + | 0.00000000657 | 0.000000611 |
| sister chromatid segregation (GO:0000819) | 136 | - | 0.00000000669 | 0.000000619 |
| posttranscriptional regulation of gene expression (GO:0010608) | 517 | + | 0.00000000697 | 0.00000064 |
| positive regulation of I-kappaB kinase/NF-kappaB signaling (GO:0043123) | 183 | + | 0.00000000705 | 0.000000644 |
| cellular response to interleukin-1 (GO:0071347) | 172 | + | 0.0000000072 | 0.000000654 |
| positive regulation of cytokine production (GO:0001819) | 436 | + | 0.00000000782 | 0.000000707 |
| plasma membrane bounded cell projection assembly (GO:0120031) | 419 | - | 0.000000008 | 0.000000718 |
| cellular lipid metabolic process (GO:0044255) | 934 | - | 0.00000000812 | 0.000000725 |
| response to unfolded protein (GO:0006986) | 157 | + | 0.00000000835 | 0.000000741 |
| ribonucleoprotein complex assembly (GO:0022618) | 219 | + | 0.00000000855 | 0.000000755 |
| interferon-gamma-mediated signaling pathway (GO:0060333) | 71 | + | 0.00000000866 | 0.00000076 |
| positive regulation of biological process (GO:0048518) | 5901 | + | 0.00000000896 | 0.000000782 |
| regulation of signal transduction (GO:0009966) | 3231 | + | 0.00000000948 | 0.000000823 |
| cell activation involved in immune response (GO:0002263) | 617 | + | 0.00000000966 | 0.000000835 |
| positive regulation of peptidase activity (GO:0010952) | 186 | + | 0.00000000975 | 0.000000838 |
| regulation of innate immune response (GO:0045088) | 437 | + | 0.0000000111 | 0.000000952 |
| regulation of cytokine-mediated signaling pathway (GO:0001959) | 157 | + | 0.0000000112 | 0.000000951 |
| regulation of response to external stimulus (GO:0032101) | 753 | + | 0.0000000122 | 0.00000103 |
| positive regulation of cellular metabolic process (GO:0031325) | 3236 | + | 0.0000000144 | 0.00000121 |
| microtubule organizing center organization (GO:0031023) | 93 | - | 0.0000000154 | 0.00000128 |
| leukocyte activation involved in immune response (GO:0002366) | 613 | + | 0.0000000157 | 0.00000131 |
| positive regulation of cysteine-type endopeptidase activity (GO:2001056) | 145 | + | 0.000000016 | 0.00000132 |
| establishment of protein localization (GO:0045184) | 1547 | + | 0.0000000161 | 0.00000132 |
| secretion (GO:0046903) | 1098 | + | 0.0000000161 | 0.00000132 |
| positive regulation of cellular process (GO:0048522) | 5197 | + | 0.0000000173 | 0.00000141 |
| regulation of cell communication (GO:0010646) | 3586 | + | 0.0000000179 | 0.00000145 |
| establishment of protein localization to organelle (GO:0072594) | 414 | + | 0.0000000188 | 0.00000152 |
| positive regulation of cell adhesion (GO:0045785) | 402 | + | 0.0000000204 | 0.00000163 |
| positive regulation of nitrogen compound metabolic process (GO:0051173) | 3094 | + | 0.0000000211 | 0.00000168 |
| regulation of cell adhesion (GO:0030155) | 665 | + | 0.0000000215 | 0.00000171 |
| positive regulation of protein metabolic process (GO:0051247) | 1662 | + | 0.0000000224 | 0.00000177 |
| negative regulation of protein metabolic process (GO:0051248) | 1077 | + | 0.0000000231 | 0.00000182 |
| positive regulation of macromolecule metabolic process (GO:0010604) | 3259 | + | 0.0000000235 | 0.00000184 |
| positive regulation of developmental process (GO:0051094) | 1343 | + | 0.0000000241 | 0.00000188 |
| regulation of signaling (GO:0023051) | 3626 | + | 0.0000000248 | 0.00000192 |
| protein transport (GO:0015031) | 1467 | + | 0.0000000253 | 0.00000195 |
| ribonucleoprotein complex subunit organization (GO:0071826) | 233 | + | 0.0000000254 | 0.00000195 |
| RNA splicing (GO:0008380) | 382 | + | 0.0000000254 | 0.00000194 |
| positive regulation of metabolic process (GO:0009893) | 3527 | + | 0.0000000269 | 0.00000204 |
| positive regulation of cysteine-type endopeptidase activity involved in apoptotic process (GO:0043280) | 128 | + | 0.0000000269 | 0.00000204 |
| centromere complex assembly (GO:0034508) | 36 | - | 0.0000000276 | 0.00000208 |
| regulation of cell migration (GO:0030334) | 819 | + | 0.0000000295 | 0.00000221 |
| lipid metabolic process (GO:0006629) | 1178 | - | 0.0000000316 | 0.00000236 |
| cell projection assembly (GO:0030031) | 433 | - | 0.0000000319 | 0.00000237 |
| positive regulation of locomotion (GO:0040017) | 537 | + | 0.0000000375 | 0.00000277 |
| response to interleukin-1 (GO:0070555) | 196 | + | 0.0000000423 | 0.00000311 |
| response to topologically incorrect protein (GO:0035966) | 180 | + | 0.0000000482 | 0.00000353 |
| regulation of DNA-binding transcription factor activity (GO:0051090) | 426 | + | 0.0000000482 | 0.00000351 |
| microtubule cytoskeleton organization involved in mitosis (GO:1902850) | 93 | - | 0.0000000516 | 0.00000374 |
| peptide transport (GO:0015833) | 1491 | + | 0.0000000528 | 0.00000381 |
| amide transport (GO:0042886) | 1522 | + | 0.0000000535 | 0.00000384 |
| positive regulation of multicellular organismal process (GO:0051240) | 1706 | + | 0.0000000585 | 0.00000418 |
| negative regulation of multi-organism process (GO:0043901) | 178 | + | 0.0000000618 | 0.0000044 |
| activation of immune response (GO:0002253) | 481 | + | 0.0000000626 | 0.00000444 |
| mitotic nuclear division (GO:0140014) | 137 | - | 0.0000000646 | 0.00000456 |
| positive regulation of defense response (GO:0031349) | 453 | + | 0.0000000664 | 0.00000467 |
| regulation of locomotion (GO:0040012) | 955 | + | 0.0000000716 | 0.00000501 |
| extrinsic apoptotic signaling pathway (GO:0097191) | 95 | + | 0.0000000748 | 0.00000521 |
| positive regulation of cellular component movement (GO:0051272) | 518 | + | 0.0000000755 | 0.00000524 |
| positive regulation of cellular protein metabolic process (GO:0032270) | 1561 | + | 0.0000000772 | 0.00000533 |
| cellular response to tumor necrosis factor (GO:0071356) | 237 | + | 0.0000000842 | 0.00000578 |
| antigen processing and presentation of endogenous peptide antigen (GO:0002483) | 13 | + | 0.0000000947 | 0.00000648 |
| antigen processing and presentation of endogenous peptide antigen via MHC class I (GO:0019885) | 13 | + | 0.0000000947 | 0.00000645 |
| intracellular protein transport (GO:0006886) | 970 | + | 0.000000107 | 0.00000724 |
| regulation of response to endoplasmic reticulum stress (GO:1905897) | 82 | + | 0.000000112 | 0.00000755 |
| mitotic spindle organization (GO:0007052) | 70 | - | 0.000000116 | 0.00000783 |
| positive regulation of cell motility (GO:2000147) | 505 | + | 0.000000117 | 0.00000785 |
| ribosomal large subunit biogenesis (GO:0042273) | 70 | + | 0.000000127 | 0.00000844 |
| lipid biosynthetic process (GO:0008610) | 566 | - | 0.00000013 | 0.00000862 |
| positive regulation of cell migration (GO:0030335) | 483 | + | 0.00000013 | 0.00000861 |
| negative regulation of signal transduction (GO:0009968) | 1213 | + | 0.000000134 | 0.00000884 |
| IRE1-mediated unfolded protein response (GO:0036498) | 55 | + | 0.000000137 | 0.00000899 |
| negative regulation of viral life cycle (GO:1903901) | 81 | + | 0.000000146 | 0.00000952 |
| meiotic nuclear division (GO:0140013) | 147 | - | 0.000000158 | 0.0000103 |
| negative regulation of cell communication (GO:0010648) | 1309 | + | 0.000000159 | 0.0000103 |
| innate immune response-activating signal transduction (GO:0002758) | 225 | + | 0.000000169 | 0.0000109 |
| immune response-activating signal transduction (GO:0002757) | 414 | + | 0.00000017 | 0.0000109 |
| meiotic cell cycle process (GO:1903046) | 161 | - | 0.000000177 | 0.0000113 |
| ATP metabolic process (GO:0046034) | 177 | + | 0.000000188 | 0.000012 |
| negative regulation of signaling (GO:0023057) | 1313 | + | 0.000000195 | 0.0000124 |
| G2/M transition of mitotic cell cycle (GO:0000086) | 135 | - | 0.000000207 | 0.0000131 |
| negative regulation of cellular protein metabolic process (GO:0032269) | 1011 | + | 0.000000217 | 0.0000137 |
| inflammatory response (GO:0006954) | 483 | + | 0.000000219 | 0.0000138 |
| regulation of inflammatory response (GO:0050727) | 325 | + | 0.000000238 | 0.0000149 |
| response to decreased oxygen levels (GO:0036293) | 349 | + | 0.000000238 | 0.0000148 |
| negative regulation of viral process (GO:0048525) | 97 | + | 0.00000025 | 0.0000155 |
| signal transduction (GO:0007165) | 4717 | + | 0.00000025 | 0.0000155 |
| mRNA processing (GO:0006397) | 454 | + | 0.000000274 | 0.0000169 |
| regulation of molecular function (GO:0065009) | 3214 | + | 0.000000279 | 0.0000171 |
| activation of cysteine-type endopeptidase activity involved in apoptotic process (GO:0006919) | 88 | + | 0.000000309 | 0.0000189 |
| positive regulation of cell differentiation (GO:0045597) | 954 | + | 0.00000031 | 0.0000189 |
| response to hypoxia (GO:0001666) | 338 | + | 0.000000311 | 0.0000188 |
| regulation of intrinsic apoptotic signaling pathway (GO:2001242) | 158 | + | 0.000000323 | 0.0000195 |
| positive regulation of cell-cell adhesion (GO:0022409) | 253 | + | 0.000000324 | 0.0000195 |
| response to oxygen levels (GO:0070482) | 372 | + | 0.000000327 | 0.0000196 |
| antigen processing and presentation of peptide antigen via MHC class I (GO:0002474) | 94 | + | 0.000000336 | 0.00002 |
| mitotic sister chromatid segregation (GO:0000070) | 104 | - | 0.000000351 | 0.0000208 |
| regulation of cellular catabolic process (GO:0031329) | 807 | + | 0.000000392 | 0.0000232 |
| cellular macromolecule catabolic process (GO:0044265) | 861 | + | 0.000000412 | 0.0000243 |
| regulation of cellular component movement (GO:0051270) | 960 | + | 0.000000451 | 0.0000265 |
| virion assembly (GO:0019068) | 37 | + | 0.000000506 | 0.0000296 |
| regulation of RNA splicing (GO:0043484) | 134 | + | 0.000000512 | 0.0000298 |
| negative regulation of nitrogen compound metabolic process (GO:0051172) | 2281 | + | 0.000000526 | 0.0000306 |
| regulation of cell motility (GO:2000145) | 880 | + | 0.000000568 | 0.0000329 |
| regulation of protein modification process (GO:0031399) | 1793 | + | 0.000000574 | 0.0000331 |
| negative regulation of cell death (GO:0060548) | 981 | + | 0.000000596 | 0.0000343 |
| macromolecule metabolic process (GO:0043170) | 5990 | + | 0.00000062 | 0.0000355 |
| activation of innate immune response (GO:0002218) | 245 | + | 0.000000634 | 0.0000362 |
| immune response-regulating signaling pathway (GO:0002764) | 451 | + | 0.000000646 | 0.0000367 |
| positive regulation of leukocyte cell-cell adhesion (GO:1903039) | 216 | + | 0.000000647 | 0.0000366 |
| cellular response to lipid (GO:0071396) | 519 | + | 0.000000651 | 0.0000367 |
| tumor necrosis factor-mediated signaling pathway (GO:0033209) | 118 | + | 0.000000657 | 0.0000369 |
| ncRNA processing (GO:0034470) | 372 | + | 0.000000668 | 0.0000375 |
| apoptotic signaling pathway (GO:0097190) | 281 | + | 0.000000672 | 0.0000376 |
| regulation of mitotic nuclear division (GO:0007088) | 189 | - | 0.000000673 | 0.0000375 |
| pattern recognition receptor signaling pathway (GO:0002221) | 122 | + | 0.000000746 | 0.0000413 |
| negative regulation of apoptotic signaling pathway (GO:2001234) | 224 | + | 0.000000747 | 0.0000413 |
| cell cycle G2/M phase transition (GO:0044839) | 137 | - | 0.000000753 | 0.0000415 |
| regulation of type I interferon production (GO:0032479) | 125 | + | 0.000000754 | 0.0000414 |
| positive regulation of response to external stimulus (GO:0032103) | 303 | + | 0.000000763 | 0.0000417 |
| antigen processing and presentation of exogenous peptide antigen via MHC class I, TAP-dependent (GO:0002479) | 76 | + | 0.000000799 | 0.0000436 |
| vesicle organization (GO:0016050) | 293 | + | 0.000000891 | 0.0000484 |
| regulation of protein modification by small protein conjugation or removal (GO:1903320) | 217 | + | 0.000000898 | 0.0000486 |
| transport (GO:0006810) | 4256 | + | 0.000000973 | 0.0000525 |
| positive regulation of gene expression (GO:0010628) | 1945 | + | 0.00000105 | 0.0000562 |
| intraciliary transport (GO:0042073) | 51 | - | 0.00000109 | 0.0000582 |
| DNA recombination (GO:0006310) | 202 | - | 0.00000109 | 0.0000583 |
| regulation of cell-cell adhesion (GO:0022407) | 387 | + | 0.00000111 | 0.0000591 |
| regulation of mRNA splicing, via spliceosome (GO:0048024) | 98 | + | 0.00000115 | 0.0000611 |
| chromatin remodeling at centromere (GO:0031055) | 32 | - | 0.00000115 | 0.0000609 |
| negative regulation of apoptotic process (GO:0043066) | 880 | + | 0.00000117 | 0.0000615 |
| positive regulation of innate immune response (GO:0045089) | 331 | + | 0.00000119 | 0.0000623 |
| regulation of cell cycle process (GO:0010564) | 759 | - | 0.00000119 | 0.0000623 |
| DNA conformation change (GO:0071103) | 266 | - | 0.00000119 | 0.0000621 |
| signaling (GO:0023052) | 5047 | + | 0.00000126 | 0.0000654 |
| cellular nitrogen compound biosynthetic process (GO:0044271) | 1571 | + | 0.00000127 | 0.0000658 |
| nitrogen compound transport (GO:0071705) | 1774 | + | 0.00000139 | 0.0000718 |
| intracellular signal transduction (GO:0035556) | 1656 | + | 0.0000016 | 0.0000821 |
| positive regulation of NIK/NF-kappaB signaling (GO:1901224) | 75 | + | 0.00000161 | 0.0000824 |
| regulation of NIK/NF-kappaB signaling (GO:1901222) | 105 | + | 0.00000161 | 0.0000822 |
| kinetochore organization (GO:0051383) | 17 | - | 0.00000165 | 0.0000843 |
| regulation of signaling receptor activity (GO:0010469) | 600 | + | 0.00000166 | 0.0000844 |
| DNA replication initiation (GO:0006270) | 32 | - | 0.00000166 | 0.0000842 |
| cell communication (GO:0007154) | 5150 | + | 0.00000167 | 0.0000842 |
| protein metabolic process (GO:0019538) | 4198 | + | 0.00000167 | 0.0000841 |
| organelle localization by membrane tethering (GO:0140056) | 168 | - | 0.00000169 | 0.000085 |
| negative regulation of cellular metabolic process (GO:0031324) | 2481 | + | 0.00000174 | 0.0000869 |
| negative regulation of programmed cell death (GO:0043069) | 897 | + | 0.00000179 | 0.0000891 |
| intrinsic apoptotic signaling pathway in response to endoplasmic reticulum stress (GO:0070059) | 32 | + | 0.00000182 | 0.0000905 |
| response to lipid (GO:0033993) | 851 | + | 0.00000187 | 0.0000928 |
| double-strand break repair (GO:0006302) | 167 | - | 0.00000196 | 0.0000966 |
| positive regulation of apoptotic signaling pathway (GO:2001235) | 177 | + | 0.00000204 | 0.0001 |
| negative regulation of mitotic sister chromatid separation (GO:2000816) | 30 | - | 0.00000205 | 0.000101 |
| antigen processing and presentation of peptide antigen via MHC class Ib (GO:0002428) | 9 | + | 0.00000214 | 0.000104 |
| regulation of G2/M transition of mitotic cell cycle (GO:0010389) | 195 | - | 0.00000215 | 0.000105 |
| antigen processing and presentation of exogenous peptide antigen via MHC class I (GO:0042590) | 80 | + | 0.00000216 | 0.000105 |
| meiosis I (GO:0007127) | 108 | - | 0.00000251 | 0.000122 |
| negative regulation of chromosome separation (GO:1905819) | 31 | - | 0.00000261 | 0.000126 |
| negative regulation of sister chromatid segregation (GO:0033046) | 35 | - | 0.00000267 | 0.000129 |
| meiosis I cell cycle process (GO:0061982) | 111 | - | 0.00000287 | 0.000138 |
| actin filament organization (GO:0007015) | 230 | + | 0.00000287 | 0.000137 |
| regulation of viral entry into host cell (GO:0046596) | 31 | + | 0.00000291 | 0.000139 |
| negative regulation of response to endoplasmic reticulum stress (GO:1903573) | 43 | + | 0.00000294 | 0.00014 |
| chromosome separation (GO:0051304) | 30 | - | 0.00000309 | 0.000147 |
| regulation of leukocyte cell-cell adhesion (GO:1903037) | 293 | + | 0.00000321 | 0.000152 |
| cellular response to oxygen-containing compound (GO:1901701) | 982 | + | 0.00000321 | 0.000151 |
| negative regulation of chromosome segregation (GO:0051985) | 36 | - | 0.00000323 | 0.000152 |
| regulation of leukocyte migration (GO:0002685) | 187 | + | 0.00000329 | 0.000154 |
| regulation of mitotic cell cycle phase transition (GO:1901990) | 412 | - | 0.00000337 | 0.000157 |
| double-strand break repair via homologous recombination (GO:0000724) | 90 | - | 0.00000349 | 0.000163 |
| intracellular transport (GO:0046907) | 1509 | + | 0.00000355 | 0.000165 |
| recombinational repair (GO:0000725) | 91 | - | 0.00000364 | 0.000169 |
| regulation of mRNA processing (GO:0050684) | 135 | + | 0.00000365 | 0.000169 |
| regulation of proteolysis involved in cellular protein catabolic process (GO:1903050) | 213 | + | 0.00000368 | 0.00017 |
| meiotic chromosome segregation (GO:0045132) | 88 | - | 0.00000369 | 0.00017 |
| macromolecule catabolic process (GO:0009057) | 1003 | + | 0.0000037 | 0.00017 |
| negative regulation of mitotic metaphase/anaphase transition (GO:0045841) | 27 | - | 0.00000379 | 0.000173 |
| regulation of cell cycle G2/M phase transition (GO:1902749) | 210 | - | 0.00000397 | 0.000181 |
| regulation of intracellular signal transduction (GO:1902531) | 1787 | + | 0.00000406 | 0.000184 |
| regulation of hydrolase activity (GO:0051336) | 1265 | + | 0.00000417 | 0.000189 |
| DNA replication-independent nucleosome assembly (GO:0006336) | 39 | - | 0.00000425 | 0.000192 |
| regulation of protein phosphorylation (GO:0001932) | 1406 | + | 0.00000475 | 0.000214 |
| negative regulation of metaphase/anaphase transition of cell cycle (GO:1902100) | 28 | - | 0.00000489 | 0.000219 |
| regulation of localization (GO:0032879) | 2672 | + | 0.00000492 | 0.00022 |
| DNA replication-independent nucleosome organization (GO:0034724) | 40 | - | 0.00000496 | 0.000222 |
| NADH regeneration (GO:0006735) | 25 | + | 0.00000559 | 0.000249 |
| glucose catabolic process to pyruvate (GO:0061718) | 25 | + | 0.00000559 | 0.000248 |
| canonical glycolysis (GO:0061621) | 25 | + | 0.00000559 | 0.000247 |
| cellular lipid catabolic process (GO:0044242) | 192 | - | 0.00000564 | 0.000249 |
| DNA strand elongation (GO:0022616) | 19 | - | 0.00000574 | 0.000253 |
| I-kappaB kinase/NF-kappaB signaling (GO:0007249) | 68 | + | 0.00000585 | 0.000257 |
| response to interleukin-12 (GO:0070671) | 49 | + | 0.00000629 | 0.000275 |
| protein-containing complex subunit organization (GO:0043933) | 1790 | + | 0.00000661 | 0.000289 |
| cellular response to hypoxia (GO:0071456) | 186 | + | 0.0000068 | 0.000296 |
| antigen processing and presentation of endogenous peptide antigen via MHC class Ib (GO:0002476) | 8 | + | 0.00000682 | 0.000296 |
| CENP-A containing nucleosome assembly (GO:0034080) | 30 | - | 0.000007 | 0.000303 |
| CENP-A containing chromatin organization (GO:0061641) | 30 | - | 0.000007 | 0.000302 |
| positive regulation of proteasomal ubiquitin-dependent protein catabolic process (GO:0032436) | 80 | + | 0.00000758 | 0.000326 |
| centrosome duplication (GO:0051298) | 29 | - | 0.0000076 | 0.000327 |
| cellular response to decreased oxygen levels (GO:0036294) | 196 | + | 0.00000786 | 0.000336 |
| positive regulation of leukocyte migration (GO:0002687) | 126 | + | 0.00000824 | 0.000352 |
| cellular protein metabolic process (GO:0044267) | 3626 | + | 0.00000834 | 0.000355 |
| intraciliary transport involved in cilium assembly (GO:0035735) | 40 | - | 0.00000852 | 0.000362 |
| antigen processing and presentation of endogenous antigen (GO:0019883) | 20 | + | 0.0000087 | 0.000369 |
| toll-like receptor signaling pathway (GO:0002224) | 93 | + | 0.00000876 | 0.00037 |
| regulation of cellular amide metabolic process (GO:0034248) | 391 | + | 0.00000918 | 0.000387 |
| glycolytic process through glucose-6-phosphate (GO:0061620) | 26 | + | 0.00000979 | 0.000412 |
| glycolytic process through fructose-6-phosphate (GO:0061615) | 26 | + | 0.00000979 | 0.000411 |
| positive regulation of RNA metabolic process (GO:0051254) | 1683 | + | 0.00000997 | 0.000417 |
| DNA geometric change (GO:0032392) | 93 | - | 0.00001 | 0.000418 |
| response to interferon-alpha (GO:0035455) | 22 | + | 0.00001 | 0.000417 |
| RNA splicing, via transesterification reactions (GO:0000375) | 287 | + | 0.0000105 | 0.000434 |
| ribosomal large subunit assembly (GO:0000027) | 30 | + | 0.0000107 | 0.000443 |
| maturation of SSU-rRNA from tricistronic rRNA transcript (SSU-rRNA, 5.8S rRNA, LSU-rRNA) (GO:0000462) | 34 | + | 0.0000108 | 0.000448 |
| spindle assembly (GO:0051225) | 80 | - | 0.000011 | 0.000453 |
| establishment of localization (GO:0051234) | 4374 | + | 0.0000113 | 0.000462 |
| regulation of immune effector process (GO:0002697) | 395 | + | 0.0000115 | 0.00047 |
| purine nucleoside bisphosphate metabolic process (GO:0034032) | 129 | - | 0.0000116 | 0.000472 |
| ribonucleoside bisphosphate metabolic process (GO:0033875) | 129 | - | 0.0000116 | 0.000471 |
| nucleoside bisphosphate metabolic process (GO:0033865) | 129 | - | 0.0000116 | 0.00047 |
| MAPK cascade (GO:0000165) | 366 | + | 0.0000118 | 0.000479 |
| regulation of cell cycle phase transition (GO:1901987) | 441 | - | 0.000012 | 0.000485 |
| cytoplasmic pattern recognition receptor signaling pathway (GO:0002753) | 35 | + | 0.000012 | 0.000484 |
| regulation of translation (GO:0006417) | 346 | + | 0.0000121 | 0.000487 |
| positive regulation of transcription by RNA polymerase II (GO:0045944) | 1184 | + | 0.000013 | 0.00052 |
| negative regulation of mitotic sister chromatid segregation (GO:0033048) | 33 | - | 0.0000136 | 0.000543 |
| regulation of MAPK cascade (GO:0043408) | 734 | + | 0.0000139 | 0.000555 |
| regulation of extrinsic apoptotic signaling pathway (GO:2001236) | 158 | + | 0.000015 | 0.000598 |
| cellular response to interleukin-12 (GO:0071349) | 48 | + | 0.0000154 | 0.000611 |
| regulation of anatomical structure morphogenesis (GO:0022603) | 1054 | + | 0.0000154 | 0.00061 |
| fatty acid oxidation (GO:0019395) | 78 | - | 0.0000162 | 0.00064 |
| regulation of alternative mRNA splicing, via spliceosome (GO:0000381) | 64 | + | 0.0000163 | 0.000642 |
| lipid oxidation (GO:0034440) | 79 | - | 0.0000169 | 0.000664 |
| regulation of ubiquitin-dependent protein catabolic process (GO:2000058) | 148 | + | 0.0000171 | 0.000671 |
| ADP metabolic process (GO:0046031) | 48 | + | 0.0000171 | 0.00067 |
| response to osmotic stress (GO:0006970) | 74 | + | 0.0000175 | 0.000684 |
| mRNA splicing, via spliceosome (GO:0000398) | 284 | + | 0.0000176 | 0.000684 |
| RNA splicing, via transesterification reactions with bulged adenosine as nucleophile (GO:0000377) | 284 | + | 0.0000176 | 0.000682 |
| non-motile cilium assembly (GO:1905515) | 46 | - | 0.0000178 | 0.000689 |
| positive regulation of T cell activation (GO:0050870) | 203 | + | 0.0000187 | 0.000724 |
| negative regulation of cellular process (GO:0048523) | 4583 | + | 0.000019 | 0.000733 |
| peptide cross-linking (GO:0018149) | 60 | + | 0.0000193 | 0.000744 |
| signal transduction by protein phosphorylation (GO:0023014) | 387 | + | 0.0000196 | 0.000751 |
| protein-containing complex assembly (GO:0065003) | 1511 | + | 0.0000199 | 0.000761 |
| response to oxygen-containing compound (GO:1901700) | 1502 | + | 0.00002 | 0.000764 |
| regulation of DNA-templated transcription in response to stress (GO:0043620) | 122 | + | 0.0000201 | 0.000764 |
| phospholipid metabolic process (GO:0006644) | 367 | - | 0.0000201 | 0.000764 |
| regulation of proteasomal protein catabolic process (GO:0061136) | 182 | + | 0.0000214 | 0.00081 |
| tRNA-containing ribonucleoprotein complex export from nucleus (GO:0071431) | 34 | - | 0.0000215 | 0.000812 |
| tRNA export from nucleus (GO:0006409) | 34 | - | 0.0000215 | 0.00081 |
| ribosomal small subunit assembly (GO:0000028) | 18 | + | 0.0000217 | 0.000818 |
| protein transport along microtubule (GO:0098840) | 66 | - | 0.000022 | 0.000824 |
| microtubule-based protein transport (GO:0099118) | 66 | - | 0.000022 | 0.000822 |
| regulation of cellular response to stress (GO:0080135) | 696 | + | 0.0000226 | 0.000844 |
| positive regulation of response to cytokine stimulus (GO:0060760) | 54 | + | 0.0000232 | 0.000863 |
| antigen processing and presentation of endogenous peptide antigen via MHC class I via ER pathway, TAP-independent (GO:0002486) | 7 | + | 0.0000232 | 0.000862 |
| antigen processing and presentation of endogenous peptide antigen via MHC class I via ER pathway (GO:0002484) | 7 | + | 0.0000232 | 0.00086 |
| regulation of protein transport (GO:0051223) | 681 | + | 0.0000234 | 0.000865 |
| mitotic spindle assembly checkpoint (GO:0007094) | 25 | - | 0.0000237 | 0.000874 |
| mitotic spindle checkpoint (GO:0071174) | 25 | - | 0.0000237 | 0.000872 |
| spindle assembly checkpoint (GO:0071173) | 25 | - | 0.0000237 | 0.00087 |
| spindle checkpoint (GO:0031577) | 25 | - | 0.0000237 | 0.000868 |
| cellular response to oxygen levels (GO:0071453) | 213 | + | 0.0000249 | 0.00091 |
| monocarboxylic acid catabolic process (GO:0072329) | 117 | - | 0.0000253 | 0.000923 |
| regulation of protein ubiquitination (GO:0031396) | 191 | + | 0.0000262 | 0.000956 |
| positive regulation of cellular biosynthetic process (GO:0031328) | 1941 | + | 0.0000266 | 0.000967 |
| positive regulation of cytokine-mediated signaling pathway (GO:0001961) | 48 | + | 0.0000273 | 0.000989 |
| vesicle budding from membrane (GO:0006900) | 96 | + | 0.0000273 | 0.000988 |
| positive regulation of biosynthetic process (GO:0009891) | 1973 | + | 0.0000274 | 0.000989 |
| protein folding (GO:0006457) | 217 | + | 0.0000275 | 0.000989 |
| maturation of SSU-rRNA (GO:0030490) | 44 | + | 0.0000275 | 0.000987 |
| fatty acid metabolic process (GO:0006631) | 307 | - | 0.0000278 | 0.000998 |
| regulation of peptide transport (GO:0090087) | 711 | + | 0.0000281 | 0.001 |
| regulation of establishment of protein localization (GO:0070201) | 725 | + | 0.0000283 | 0.00101 |
| regulation of multicellular organismal process (GO:0051239) | 3062 | + | 0.0000283 | 0.00101 |
| antigen processing and presentation of exogenous peptide antigen via MHC class I, TAP-independent (GO:0002480) | 9 | + | 0.0000296 | 0.00105 |
| MyD88-independent toll-like receptor signaling pathway (GO:0002756) | 31 | + | 0.0000301 | 0.00107 |
| positive regulation of supramolecular fiber organization (GO:1902905) | 198 | + | 0.0000317 | 0.00112 |
| cellular amide metabolic process (GO:0043603) | 765 | + | 0.0000324 | 0.00114 |
| membrane docking (GO:0022406) | 177 | - | 0.0000328 | 0.00115 |
| negative regulation of protein modification by small protein conjugation or removal (GO:1903321) | 84 | + | 0.0000336 | 0.00118 |
| regulation of viral genome replication (GO:0045069) | 92 | + | 0.0000341 | 0.0012 |
| regulation of proteasomal ubiquitin-dependent protein catabolic process (GO:0032434) | 120 | + | 0.0000348 | 0.00122 |
| lipid catabolic process (GO:0016042) | 297 | - | 0.0000349 | 0.00122 |
| acyl-CoA metabolic process (GO:0006637) | 94 | - | 0.0000359 | 0.00125 |
| thioester metabolic process (GO:0035383) | 94 | - | 0.0000359 | 0.00125 |
| regulation of transcription from RNA polymerase II promoter in response to stress (GO:0043618) | 116 | + | 0.000036 | 0.00125 |
| vesicle targeting (GO:0006903) | 89 | + | 0.0000386 | 0.00134 |
| positive regulation of proteasomal protein catabolic process (GO:1901800) | 105 | + | 0.0000393 | 0.00136 |
| mitotic nuclear envelope disassembly (GO:0007077) | 12 | - | 0.0000394 | 0.00136 |
| positive regulation of molecular function (GO:0044093) | 1756 | + | 0.0000395 | 0.00136 |
| interleukin-1-mediated signaling pathway (GO:0070498) | 96 | + | 0.00004 | 0.00137 |
| regulation of cysteine-type endopeptidase activity involved in apoptotic signaling pathway (GO:2001267) | 17 | + | 0.0000404 | 0.00138 |
| regulation of interleukin-1 beta production (GO:0032651) | 69 | + | 0.0000406 | 0.00139 |
| negative regulation of molecular function (GO:0044092) | 1118 | + | 0.0000422 | 0.00144 |
| positive regulation of ubiquitin-dependent protein catabolic process (GO:2000060) | 94 | + | 0.0000424 | 0.00144 |
| regulation of protein deubiquitination (GO:0090085) | 12 | + | 0.0000445 | 0.00151 |
| regulation of developmental process (GO:0050793) | 2529 | + | 0.0000448 | 0.00152 |
| autophagy (GO:0006914) | 254 | + | 0.0000455 | 0.00154 |
| process utilizing autophagic mechanism (GO:0061919) | 254 | + | 0.0000455 | 0.00153 |
| DNA duplex unwinding (GO:0032508) | 83 | - | 0.0000457 | 0.00154 |
| necrotic cell death (GO:0070265) | 28 | + | 0.0000462 | 0.00155 |
| positive regulation of chromosome segregation (GO:0051984) | 29 | - | 0.0000463 | 0.00155 |
| lipopolysaccharide-mediated signaling pathway (GO:0031663) | 34 | + | 0.0000476 | 0.00159 |
| regulation of necrotic cell death (GO:0010939) | 26 | + | 0.0000488 | 0.00163 |
| negative regulation of RNA splicing (GO:0033119) | 26 | + | 0.000051 | 0.0017 |
| regulation of cytokine biosynthetic process (GO:0042035) | 100 | + | 0.0000523 | 0.00174 |
| positive regulation of macromolecule biosynthetic process (GO:0010557) | 1855 | + | 0.0000525 | 0.00174 |
| regulation of catalytic activity (GO:0050790) | 2284 | + | 0.0000531 | 0.00175 |
| humoral immune response (GO:0006959) | 220 | + | 0.000054 | 0.00178 |
| fatty acid catabolic process (GO:0009062) | 94 | - | 0.0000543 | 0.00179 |
| TRIF-dependent toll-like receptor signaling pathway (GO:0035666) | 29 | + | 0.0000552 | 0.00181 |
| positive regulation of nucleic acid-templated transcription (GO:1903508) | 1597 | + | 0.0000555 | 0.00182 |
| response to interferon-beta (GO:0035456) | 29 | + | 0.0000557 | 0.00182 |
| regulation of cell population proliferation (GO:0042127) | 1604 | + | 0.0000562 | 0.00184 |
| regulation of nuclear division (GO:0051783) | 213 | - | 0.0000585 | 0.0019 |
| regulation of peptidyl-tyrosine phosphorylation (GO:0050730) | 247 | + | 0.0000597 | 0.00194 |
| negative regulation of viral genome replication (GO:0045071) | 57 | + | 0.00006 | 0.00195 |
| negative regulation of cysteine-type endopeptidase activity involved in apoptotic process (GO:0043154) | 86 | + | 0.0000603 | 0.00195 |
| DNA strand elongation involved in DNA replication (GO:0006271) | 14 | - | 0.0000621 | 0.00201 |
| regulation of catabolic process (GO:0009894) | 966 | + | 0.0000629 | 0.00203 |
| positive regulation of RNA biosynthetic process (GO:1902680) | 1598 | + | 0.0000634 | 0.00204 |
| negative regulation of mitotic nuclear division (GO:0045839) | 43 | - | 0.0000654 | 0.0021 |
| ISG15-protein conjugation (GO:0032020) | 6 | + | 0.0000669 | 0.00215 |
| protein localization to microtubule cytoskeleton (GO:0072698) | 33 | - | 0.0000675 | 0.00216 |
| negative regulation of immune response (GO:0050777) | 146 | + | 0.0000682 | 0.00218 |
| interleukin-12-mediated signaling pathway (GO:0035722) | 46 | + | 0.0000689 | 0.00219 |
| cell cycle checkpoint (GO:0000075) | 193 | - | 0.0000715 | 0.00227 |
| Ras protein signal transduction (GO:0007265) | 237 | + | 0.0000715 | 0.00227 |
| secondary alcohol biosynthetic process (GO:1902653) | 42 | - | 0.0000722 | 0.00229 |
| cholesterol biosynthetic process (GO:0006695) | 42 | - | 0.0000722 | 0.00228 |
| tRNA transport (GO:0051031) | 36 | - | 0.0000726 | 0.00229 |
| regulation of cholesterol biosynthetic process (GO:0045540) | 43 | - | 0.0000734 | 0.00231 |
| regulation of sterol biosynthetic process (GO:0106118) | 43 | - | 0.0000734 | 0.00231 |
| glycerophospholipid metabolic process (GO:0006650) | 298 | - | 0.0000756 | 0.00237 |
| ATP generation from ADP (GO:0006757) | 40 | + | 0.0000764 | 0.00239 |
| positive regulation of cellular catabolic process (GO:0031331) | 354 | + | 0.0000775 | 0.00242 |
| protein localization (GO:0008104) | 2079 | + | 0.0000802 | 0.0025 |
| regulation of cell differentiation (GO:0045595) | 1780 | + | 0.0000803 | 0.0025 |
| regulation of hemopoiesis (GO:1903706) | 443 | + | 0.0000825 | 0.00256 |
| positive regulation of proteolysis involved in cellular protein catabolic process (GO:1903052) | 121 | + | 0.0000825 | 0.00256 |
| regulation of lymphocyte migration (GO:2000401) | 59 | + | 0.0000877 | 0.00271 |
| nitrogen compound metabolic process (GO:0006807) | 6778 | + | 0.0000887 | 0.00274 |
| positive regulation of cytokine production involved in immune response (GO:0002720) | 55 | + | 0.0000934 | 0.00288 |
| negative regulation of protein modification process (GO:0031400) | 579 | + | 0.0000937 | 0.00288 |
| programmed necrotic cell death (GO:0097300) | 27 | + | 0.000094 | 0.00288 |
| purine ribonucleoside diphosphate metabolic process (GO:0009179) | 60 | + | 0.0000981 | 0.00301 |
| purine nucleoside diphosphate metabolic process (GO:0009135) | 60 | + | 0.0000981 | 0.003 |
| regulation of phosphorus metabolic process (GO:0051174) | 1738 | + | 0.000103 | 0.00315 |
| negative regulation of cysteine-type endopeptidase activity (GO:2000117) | 93 | + | 0.000105 | 0.0032 |
| vesicle targeting, to, from or within Golgi (GO:0048199) | 70 | + | 0.000106 | 0.00323 |
| protection from natural killer cell mediated cytotoxicity (GO:0042270) | 6 | + | 0.000107 | 0.00326 |
| leukocyte aggregation (GO:0070486) | 10 | + | 0.000108 | 0.00326 |
| Golgi vesicle budding (GO:0048194) | 77 | + | 0.000108 | 0.00326 |
| negative regulation of protein ubiquitination (GO:0031397) | 73 | + | 0.000111 | 0.00335 |
| protein localization to centrosome (GO:0071539) | 20 | - | 0.000112 | 0.00338 |
| protein localization to microtubule organizing center (GO:1905508) | 20 | - | 0.000112 | 0.00338 |
| positive regulation of MAPK cascade (GO:0043410) | 530 | + | 0.000117 | 0.00352 |
| regulation of protein localization (GO:0032880) | 987 | + | 0.000119 | 0.00356 |
| glycolytic process (GO:0006096) | 38 | + | 0.00012 | 0.0036 |
| chromosome organization involved in meiotic cell cycle (GO:0070192) | 65 | - | 0.00012 | 0.00359 |
| neutrophil chemotaxis (GO:0030593) | 82 | + | 0.000121 | 0.0036 |
| release of cytochrome c from mitochondria (GO:0001836) | 21 | + | 0.000122 | 0.00361 |
| regulation of type I interferon-mediated signaling pathway (GO:0060338) | 30 | + | 0.000124 | 0.00366 |
| positive regulation of type I interferon production (GO:0032481) | 78 | + | 0.000124 | 0.00368 |
| regulation of growth (GO:0040008) | 684 | + | 0.000126 | 0.00373 |
| regulation of phosphate metabolic process (GO:0019220) | 1736 | + | 0.000129 | 0.00381 |
| positive regulation of cellular amide metabolic process (GO:0034250) | 147 | + | 0.000131 | 0.00385 |
| regulation of T cell activation (GO:0050863) | 311 | + | 0.000132 | 0.00389 |
| regulation of mitotic cell cycle (GO:0007346) | 638 | - | 0.000132 | 0.00388 |
| nuclear DNA replication (GO:0033260) | 40 | - | 0.000134 | 0.00393 |
| regulation of cellular protein catabolic process (GO:1903362) | 246 | + | 0.000136 | 0.00398 |
| fatty acid beta-oxidation (GO:0006635) | 58 | - | 0.000136 | 0.00397 |
| ribonucleoside diphosphate metabolic process (GO:0009185) | 62 | + | 0.000136 | 0.00397 |
| response to growth factor (GO:0070848) | 526 | + | 0.000138 | 0.004 |
| antimicrobial humoral response (GO:0019730) | 108 | + | 0.00014 | 0.00405 |
| glycerolipid metabolic process (GO:0046486) | 371 | - | 0.000145 | 0.00418 |
| vesicle coating (GO:0006901) | 67 | + | 0.000145 | 0.00419 |
| glucose catabolic process (GO:0006007) | 29 | + | 0.000146 | 0.00422 |
| cell cycle DNA replication (GO:0044786) | 41 | - | 0.000147 | 0.00422 |
| positive regulation of hydrolase activity (GO:0051345) | 756 | + | 0.000147 | 0.00422 |
| regulation of gene expression (GO:0010468) | 4373 | + | 0.000148 | 0.00423 |
| phospholipid biosynthetic process (GO:0008654) | 244 | - | 0.000149 | 0.00427 |
| translational elongation (GO:0006414) | 124 | + | 0.000149 | 0.00427 |
| meiotic chromosome separation (GO:0051307) | 21 | - | 0.000151 | 0.0043 |
| organic substance transport (GO:0071702) | 2137 | + | 0.000152 | 0.00432 |
| interstrand cross-link repair (GO:0036297) | 51 | - | 0.000153 | 0.00435 |
| cellular macromolecule localization (GO:0070727) | 1542 | + | 0.000156 | 0.00443 |
| positive regulation of catabolic process (GO:0009896) | 419 | + | 0.000157 | 0.00446 |
| macromolecule biosynthetic process (GO:0009059) | 1712 | + | 0.000161 | 0.00454 |
| mitochondrion organization (GO:0007005) | 414 | + | 0.000161 | 0.00455 |
| positive regulation of nucleobase-containing compound metabolic process (GO:0045935) | 1837 | + | 0.000162 | 0.00457 |
| positive regulation of protein modification process (GO:0031401) | 1198 | + | 0.000164 | 0.00461 |
| regulation of centrosome cycle (GO:0046605) | 59 | - | 0.000165 | 0.00463 |
| organonitrogen compound biosynthetic process (GO:1901566) | 1365 | + | 0.00017 | 0.00476 |
| nucleotide-binding oligomerization domain containing signaling pathway (GO:0070423) | 27 | + | 0.000171 | 0.00478 |
| positive regulation of protein phosphorylation (GO:0001934) | 986 | + | 0.000172 | 0.00479 |
| ncRNA export from nucleus (GO:0097064) | 39 | - | 0.000172 | 0.0048 |
| negative regulation of cell population proliferation (GO:0008285) | 678 | + | 0.000174 | 0.00483 |
| cellular response to growth factor stimulus (GO:0071363) | 495 | + | 0.000175 | 0.00485 |
| reciprocal meiotic recombination (GO:0007131) | 49 | - | 0.000175 | 0.00484 |
| negative regulation of proteolysis (GO:0045861) | 346 | + | 0.000177 | 0.0049 |
| regulation of angiogenesis (GO:0045765) | 286 | + | 0.000178 | 0.00491 |
| cellular protein localization (GO:0034613) | 1535 | + | 0.000183 | 0.00506 |
| homologous recombination (GO:0035825) | 50 | - | 0.000185 | 0.0051 |
| negative regulation of intrinsic apoptotic signaling pathway (GO:2001243) | 95 | + | 0.000191 | 0.00525 |
| centriole replication (GO:0007099) | 18 | - | 0.000194 | 0.00533 |
| regulation of macromolecule metabolic process (GO:0060255) | 6010 | + | 0.000197 | 0.00539 |
| myeloid leukocyte migration (GO:0097529) | 124 | + | 0.000199 | 0.00543 |
| ncRNA metabolic process (GO:0034660) | 455 | + | 0.000199 | 0.00543 |
| organic hydroxy compound metabolic process (GO:1901615) | 442 | - | 0.000199 | 0.00542 |
| regulation of endoplasmic reticulum unfolded protein response (GO:1900101) | 27 | + | 0.000201 | 0.00546 |
| antigen processing and presentation (GO:0019882) | 212 | + | 0.000202 | 0.00549 |
| negative regulation of type I interferon production (GO:0032480) | 44 | + | 0.000203 | 0.0055 |
| intracellular receptor signaling pathway (GO:0030522) | 165 | + | 0.000204 | 0.00553 |
| regulation of phosphorylation (GO:0042325) | 1550 | + | 0.000205 | 0.00555 |
| positive regulation of transcription, DNA-templated (GO:0045893) | 1513 | + | 0.000206 | 0.00555 |
| cellular macromolecule biosynthetic process (GO:0034645) | 1658 | + | 0.000206 | 0.00555 |
| negative regulation of multicellular organismal process (GO:0051241) | 1173 | + | 0.000209 | 0.00563 |
| positive regulation of cytokinesis (GO:0032467) | 39 | - | 0.000211 | 0.00565 |
| organonitrogen compound metabolic process (GO:1901564) | 5170 | + | 0.000215 | 0.00576 |
| kinetochore assembly (GO:0051382) | 12 | - | 0.000215 | 0.00575 |
| COPII-coated vesicle budding (GO:0090114) | 69 | + | 0.000219 | 0.00585 |
| protein localization to cytoskeleton (GO:0044380) | 39 | - | 0.000221 | 0.00588 |
| homophilic cell adhesion via plasma membrane adhesion molecules (GO:0007156) | 167 | - | 0.000221 | 0.00588 |
| negative regulation of catalytic activity (GO:0043086) | 781 | + | 0.000225 | 0.00599 |
| regulation of response to biotic stimulus (GO:0002831) | 137 | + | 0.000228 | 0.00604 |
| organophosphate biosynthetic process (GO:0090407) | 516 | - | 0.000229 | 0.00605 |
| positive regulation of T cell mediated immunity (GO:0002711) | 46 | + | 0.000229 | 0.00606 |
| regulation of vasculature development (GO:1901342) | 316 | + | 0.000236 | 0.00622 |
| regulation of ribonuclease activity (GO:0060700) | 9 | + | 0.000238 | 0.00626 |
| regulation of extrinsic apoptotic signaling pathway in absence of ligand (GO:2001239) | 48 | + | 0.000242 | 0.00637 |
| regulation of necroptotic process (GO:0060544) | 17 | + | 0.000243 | 0.00639 |
| regulation of mRNA catabolic process (GO:0061013) | 200 | + | 0.000244 | 0.00639 |
| granulocyte chemotaxis (GO:0071621) | 89 | + | 0.000249 | 0.00651 |
| nucleotide-binding domain, leucine rich repeat containing receptor signaling pathway (GO:0035872) | 28 | + | 0.000252 | 0.00658 |
| protein localization to chromosome, centromeric region (GO:0071459) | 17 | - | 0.000253 | 0.0066 |
| regulation of DNA-dependent DNA replication (GO:0090329) | 53 | - | 0.000259 | 0.00675 |
| regulation of DNA replication (GO:0006275) | 108 | - | 0.00026 | 0.00675 |
| positive regulation of intrinsic apoptotic signaling pathway (GO:2001244) | 58 | + | 0.00026 | 0.00675 |
| regulation of retrograde protein transport, ER to cytosol (GO:1904152) | 13 | + | 0.000264 | 0.00683 |
| regulation of transport (GO:0051049) | 1812 | + | 0.000266 | 0.00687 |
| positive regulation of phosphorus metabolic process (GO:0010562) | 1104 | + | 0.000267 | 0.00689 |
| positive regulation of phosphate metabolic process (GO:0045937) | 1104 | + | 0.000267 | 0.00688 |
| regulation of interferon-gamma production (GO:0032649) | 100 | + | 0.000282 | 0.00726 |
| positive regulation of viral process (GO:0048524) | 104 | + | 0.000285 | 0.00734 |
| endosome organization (GO:0007032) | 75 | + | 0.000289 | 0.00741 |
| neutrophil migration (GO:1990266) | 91 | + | 0.0003 | 0.00767 |
| regulation of chemokine production (GO:0032642) | 78 | + | 0.000309 | 0.00791 |
| COPII vesicle coating (GO:0048208) | 63 | + | 0.00032 | 0.00818 |
| vesicle targeting, rough ER to cis-Golgi (GO:0048207) | 63 | + | 0.00032 | 0.00816 |
| sister chromatid cohesion (GO:0007062) | 47 | - | 0.000322 | 0.0082 |
| positive regulation of viral entry into host cell (GO:0046598) | 10 | + | 0.000323 | 0.00821 |
| positive regulation of leukocyte activation (GO:0002696) | 314 | + | 0.000324 | 0.00821 |
| aminoacyl-tRNA metabolism involved in translational fidelity (GO:0106074) | 12 | - | 0.000325 | 0.00822 |
| regulation of RNA stability (GO:0043487) | 184 | + | 0.00033 | 0.00833 |
| negative regulation of innate immune response (GO:0045824) | 56 | + | 0.000331 | 0.00835 |
| negative regulation of phosphorus metabolic process (GO:0010563) | 544 | + | 0.000336 | 0.00848 |
| leukocyte migration (GO:0050900) | 304 | + | 0.000338 | 0.0085 |
| cell cycle G1/S phase transition (GO:0044843) | 119 | - | 0.000338 | 0.00849 |
| positive regulation of hemopoiesis (GO:1903708) | 181 | + | 0.00034 | 0.00854 |
| protein exit from endoplasmic reticulum (GO:0032527) | 22 | + | 0.000342 | 0.00856 |
| regulation of tumor necrosis factor superfamily cytokine production (GO:1903555) | 138 | + | 0.000344 | 0.0086 |
| response to nitrogen compound (GO:1901698) | 1002 | + | 0.000345 | 0.00861 |
| negative regulation of extrinsic apoptotic signaling pathway in absence of ligand (GO:2001240) | 37 | + | 0.000346 | 0.00863 |
| negative regulation of signal transduction in absence of ligand (GO:1901099) | 37 | + | 0.000346 | 0.00861 |
| Rab protein signal transduction (GO:0032482) | 69 | + | 0.000348 | 0.00864 |
| positive regulation of T cell cytokine production (GO:0002726) | 19 | + | 0.000349 | 0.00866 |
| negative regulation of necrotic cell death (GO:0060547) | 16 | + | 0.00035 | 0.00868 |
| regulation of reactive oxygen species biosynthetic process (GO:1903426) | 84 | + | 0.000351 | 0.00869 |
| cell chemotaxis (GO:0060326) | 201 | + | 0.000357 | 0.00882 |
| regulation of cell growth (GO:0001558) | 408 | + | 0.000362 | 0.00892 |
| NIK/NF-kappaB signaling (GO:0038061) | 81 | + | 0.000364 | 0.00895 |
| negative regulation of peptidase activity (GO:0010466) | 252 | + | 0.000364 | 0.00895 |
| supramolecular fiber organization (GO:0097435) | 440 | + | 0.000365 | 0.00895 |
| cellular nitrogen compound metabolic process (GO:0034641) | 3283 | + | 0.000365 | 0.00895 |
| negative regulation of mRNA splicing, via spliceosome (GO:0048025) | 21 | + | 0.000366 | 0.00896 |
| protein-DNA complex assembly (GO:0065004) | 193 | - | 0.000369 | 0.00901 |
| positive regulation of actin filament polymerization (GO:0030838) | 93 | + | 0.000372 | 0.00907 |
| positive regulation of chemotaxis (GO:0050921) | 133 | + | 0.000376 | 0.00915 |
| DNA integrity checkpoint (GO:0031570) | 143 | - | 0.000377 | 0.00917 |
| regulation of transcription from RNA polymerase II promoter in response to hypoxia (GO:0061418) | 76 | + | 0.000379 | 0.00919 |
| negative regulation of extrinsic apoptotic signaling pathway (GO:2001237) | 104 | + | 0.000389 | 0.00944 |
| multivesicular body organization (GO:0036257) | 31 | + | 0.00039 | 0.00945 |
| positive regulation of inflammatory response (GO:0050729) | 136 | + | 0.000391 | 0.00944 |
| chemotaxis (GO:0006935) | 533 | + | 0.000397 | 0.00957 |
| positive regulation of transport (GO:0051050) | 956 | + | 0.000403 | 0.0097 |
| vesicle fusion with endoplasmic reticulum-Golgi intermediate compartment (ERGIC) membrane (GO:1990668) | 6 | + | 0.000403 | 0.00969 |
| negative regulation of phosphate metabolic process (GO:0045936) | 543 | + | 0.000404 | 0.0097 |
| positive regulation of DNA-directed DNA polymerase activity (GO:1900264) | 7 | - | 0.000407 | 0.00976 |
| regulation of DNA-directed DNA polymerase activity (GO:1900262) | 7 | - | 0.000407 | 0.00975 |
| alcohol metabolic process (GO:0006066) | 306 | - | 0.000412 | 0.00985 |
| positive regulation of reactive oxygen species metabolic process (GO:2000379) | 95 | + | 0.000415 | 0.0099 |
| regulation of reactive oxygen species metabolic process (GO:2000377) | 175 | + | 0.000415 | 0.00988 |
| protein localization to chromosome (GO:0034502) | 63 | - | 0.000415 | 0.00988 |
| response to oxidative stress (GO:0006979) | 374 | + | 0.000418 | 0.00993 |
| positive regulation of cellular protein catabolic process (GO:1903364) | 141 | + | 0.000422 | 0.01 |
| metaphase/anaphase transition of mitotic cell cycle (GO:0007091) | 5 | - | 0.000425 | 0.0101 |
| metaphase/anaphase transition of cell cycle (GO:0044784) | 5 | - | 0.000425 | 0.0101 |
| viral budding via host ESCRT complex (GO:0039702) | 20 | + | 0.000429 | 0.0101 |
| positive regulation of phosphorylation (GO:0042327) | 1037 | + | 0.00043 | 0.0101 |
| cellular catabolic process (GO:0044248) | 1715 | + | 0.000435 | 0.0103 |
| mitotic cell cycle checkpoint (GO:0007093) | 156 | - | 0.00044 | 0.0103 |
| positive regulation of miRNA metabolic process (GO:2000630) | 5 | + | 0.000442 | 0.0104 |
| positive regulation of leukocyte chemotaxis (GO:0002690) | 85 | + | 0.000444 | 0.0104 |
| regulation of leukocyte activation (GO:0002694) | 499 | + | 0.000445 | 0.0104 |
| interleukin-35-mediated signaling pathway (GO:0070757) | 11 | + | 0.000453 | 0.0106 |
| taxis (GO:0042330) | 536 | + | 0.000455 | 0.0106 |
| positive regulation of lymphocyte activation (GO:0051251) | 271 | + | 0.000456 | 0.0106 |
| positive regulation of cell activation (GO:0050867) | 326 | + | 0.000458 | 0.0107 |
| regulation of cholesterol metabolic process (GO:0090181) | 56 | - | 0.000461 | 0.0107 |
| ATP-dependent chromatin remodeling (GO:0043044) | 71 | - | 0.000466 | 0.0108 |
| regulation of lymphocyte activation (GO:0051249) | 417 | + | 0.000475 | 0.011 |
| secondary alcohol metabolic process (GO:1902652) | 126 | - | 0.000476 | 0.011 |
| regulation of microtubule cytoskeleton organization (GO:0070507) | 189 | - | 0.000478 | 0.0111 |
| CDP-diacylglycerol biosynthetic process (GO:0016024) | 13 | - | 0.00048 | 0.0111 |
| neurotrophin TRK receptor signaling pathway (GO:0048011) | 20 | + | 0.000482 | 0.0111 |
| meiotic chromosome condensation (GO:0010032) | 6 | - | 0.000483 | 0.0111 |
| regulation of interleukin-1 production (GO:0032652) | 82 | + | 0.000497 | 0.0114 |
| positive regulation of translation (GO:0045727) | 127 | + | 0.000518 | 0.0119 |
| interleukin-27-mediated signaling pathway (GO:0070106) | 11 | + | 0.000518 | 0.0119 |
| cellular response to stimulus (GO:0051716) | 6280 | + | 0.000518 | 0.0119 |
| negative regulation of intracellular signal transduction (GO:1902532) | 488 | + | 0.000534 | 0.0122 |
| regulation of mRNA stability (GO:0043488) | 178 | + | 0.000545 | 0.0124 |
| nuclear pore organization (GO:0006999) | 14 | - | 0.000546 | 0.0124 |
| mRNA 3'-end processing (GO:0031124) | 81 | + | 0.00055 | 0.0125 |
| telomere maintenance via semi-conservative replication (GO:0032201) | 26 | - | 0.000559 | 0.0127 |
| NAD metabolic process (GO:0019674) | 42 | + | 0.00056 | 0.0127 |
| regulation of cellular protein localization (GO:1903827) | 526 | + | 0.000561 | 0.0127 |
| response to stimulus (GO:0050896) | 7888 | + | 0.000565 | 0.0128 |
| negative regulation of defense response (GO:0031348) | 200 | + | 0.000566 | 0.0128 |
| regulation of cellular response to growth factor stimulus (GO:0090287) | 266 | + | 0.000571 | 0.0129 |
| G1/S transition of mitotic cell cycle (GO:0000082) | 118 | - | 0.000578 | 0.013 |
| protein sumoylation (GO:0016925) | 64 | - | 0.000582 | 0.0131 |
| negative regulation of viral entry into host cell (GO:0046597) | 19 | + | 0.000589 | 0.0132 |
| cholesterol metabolic process (GO:0008203) | 117 | - | 0.000589 | 0.0132 |
| regulation of cell-substrate adhesion (GO:0010810) | 206 | + | 0.000591 | 0.0132 |
| nucleotide phosphorylation (GO:0046939) | 59 | + | 0.000593 | 0.0133 |
| DNA replication checkpoint (GO:0000076) | 16 | - | 0.000593 | 0.0132 |
| membrane organization (GO:0061024) | 748 | + | 0.000595 | 0.0133 |
| positive regulation of reactive oxygen species biosynthetic process (GO:1903428) | 51 | + | 0.000601 | 0.0134 |
| nucleotide-sugar biosynthetic process (GO:0009226) | 23 | + | 0.000612 | 0.0136 |
| granulocyte migration (GO:0097530) | 99 | + | 0.000614 | 0.0136 |
| leukocyte chemotaxis (GO:0030595) | 139 | + | 0.00063 | 0.014 |
| positive regulation of receptor signaling pathway via STAT (GO:1904894) | 87 | + | 0.00063 | 0.014 |
| metabolic process (GO:0008152) | 8128 | + | 0.000639 | 0.0141 |
| cellular response to endogenous stimulus (GO:0071495) | 1143 | + | 0.000645 | 0.0142 |
| negative regulation of programmed necrotic cell death (GO:0062099) | 12 | + | 0.000654 | 0.0144 |
| negative regulation of necroptotic process (GO:0060546) | 12 | + | 0.000654 | 0.0144 |
| CDP-diacylglycerol metabolic process (GO:0046341) | 14 | - | 0.000655 | 0.0144 |
| histone exchange (GO:0043486) | 45 | - | 0.000657 | 0.0144 |
| DNA-dependent DNA replication maintenance of fidelity (GO:0045005) | 40 | - | 0.000661 | 0.0145 |
| lipid modification (GO:0030258) | 198 | - | 0.000666 | 0.0146 |
| protein import into mitochondrial matrix (GO:0030150) | 19 | + | 0.000667 | 0.0146 |
| monocarboxylic acid metabolic process (GO:0032787) | 498 | - | 0.000673 | 0.0147 |
| microtubule-based movement (GO:0007018) | 260 | - | 0.000679 | 0.0148 |
| macromolecule localization (GO:0033036) | 2397 | + | 0.00068 | 0.0148 |
| positive regulation of peptidyl-tyrosine phosphorylation (GO:0050731) | 185 | + | 0.000683 | 0.0148 |
| negative regulation of endopeptidase activity (GO:0010951) | 243 | + | 0.000686 | 0.0149 |
| autocrine signaling (GO:0035425) | 7 | + | 0.0007 | 0.0152 |
| regulation of tumor necrosis factor production (GO:0032680) | 134 | + | 0.000701 | 0.0152 |
| regulation of intracellular protein transport (GO:0033157) | 241 | + | 0.000702 | 0.0152 |
| T cell activation (GO:0042110) | 226 | + | 0.000714 | 0.0154 |
| viral budding (GO:0046755) | 24 | + | 0.000717 | 0.0155 |
| regulation of stress-activated protein kinase signaling cascade (GO:0070302) | 225 | + | 0.000728 | 0.0157 |
| regulation of stress-activated MAPK cascade (GO:0032872) | 223 | + | 0.000731 | 0.0157 |
| alcohol biosynthetic process (GO:0046165) | 113 | - | 0.000733 | 0.0157 |
| sensory perception (GO:0007600) | 893 | - | 0.000748 | 0.016 |
| catabolic process (GO:0009056) | 1981 | + | 0.000749 | 0.016 |
| regulation of leukocyte chemotaxis (GO:0002688) | 110 | + | 0.000752 | 0.0161 |
| positive regulation of protein catabolic process (GO:0045732) | 218 | + | 0.000753 | 0.0161 |
| antigen processing and presentation via MHC class Ib (GO:0002475) | 16 | + | 0.000776 | 0.0165 |
| cellular response to drug (GO:0035690) | 330 | + | 0.000812 | 0.0173 |
| detection of chemical stimulus (GO:0009593) | 465 | - | 0.000829 | 0.0176 |
| positive regulation of cell-substrate adhesion (GO:0010811) | 121 | + | 0.000834 | 0.0177 |
| resolution of meiotic recombination intermediates (GO:0000712) | 17 | - | 0.000847 | 0.018 |
| negative regulation of phosphorylation (GO:0042326) | 431 | + | 0.000852 | 0.018 |
| membrane lipid metabolic process (GO:0006643) | 200 | - | 0.000852 | 0.018 |
| toll-like receptor 2 signaling pathway (GO:0034134) | 5 | + | 0.000863 | 0.0182 |
| multivesicular body assembly (GO:0036258) | 30 | + | 0.000902 | 0.019 |
| sterol metabolic process (GO:0016125) | 132 | - | 0.000921 | 0.0194 |
| regulation of cell activation (GO:0050865) | 536 | + | 0.000929 | 0.0196 |
| nucleic acid metabolic process (GO:0090304) | 2180 | + | 0.000952 | 0.02 |
| regulation of metabolic process (GO:0019222) | 6522 | + | 0.000956 | 0.0201 |
| sterol biosynthetic process (GO:0016126) | 47 | - | 0.000956 | 0.02 |
| regulation of chemotaxis (GO:0050920) | 208 | + | 0.00096 | 0.0201 |
| vesicle fusion with Golgi apparatus (GO:0048280) | 8 | + | 0.000973 | 0.0203 |
| ATP synthesis coupled electron transport (GO:0042773) | 77 | + | 0.000977 | 0.0204 |
| acyl-CoA biosynthetic process (GO:0071616) | 43 | - | 0.00101 | 0.0211 |
| thioester biosynthetic process (GO:0035384) | 43 | - | 0.00101 | 0.0211 |
| regulation of T cell migration (GO:2000404) | 41 | + | 0.00102 | 0.0211 |
| regulation of protein folding (GO:1903332) | 10 | + | 0.00102 | 0.0211 |
| positive regulation of cell cycle process (GO:0090068) | 287 | - | 0.00102 | 0.0212 |
| respiratory electron transport chain (GO:0022904) | 96 | + | 0.00103 | 0.0213 |
| cellular response to DNA damage stimulus (GO:0006974) | 749 | - | 0.00103 | 0.0213 |
| response to endogenous stimulus (GO:0009719) | 1416 | + | 0.00103 | 0.0213 |
| regulation of cytokine production involved in immune response (GO:0002718) | 85 | + | 0.00103 | 0.0213 |
| RNA localization (GO:0006403) | 204 | + | 0.00104 | 0.0214 |
| regulation of protein catabolic process (GO:0042176) | 385 | + | 0.00105 | 0.0217 |
| enzyme linked receptor protein signaling pathway (GO:0007167) | 707 | + | 0.00106 | 0.0217 |
| intrinsic apoptotic signaling pathway (GO:0097193) | 150 | + | 0.00106 | 0.0217 |
| positive regulation of adaptive immune response based on somatic recombination of immune receptors built from immunoglobulin superfamily domains (GO:0002824) | 97 | + | 0.00107 | 0.0219 |
| protein transmembrane transport (GO:0071806) | 62 | + | 0.00108 | 0.022 |
| extracellular matrix organization (GO:0030198) | 335 | + | 0.00108 | 0.022 |
| positive regulation of cytoskeleton organization (GO:0051495) | 219 | + | 0.00108 | 0.0221 |
| electron transport chain (GO:0022900) | 164 | + | 0.00108 | 0.022 |
| membrane fusion (GO:0061025) | 126 | + | 0.0011 | 0.0223 |
| localization (GO:0051179) | 5427 | + | 0.00112 | 0.0228 |
| amino-acid betaine metabolic process (GO:0006577) | 17 | - | 0.00112 | 0.0228 |
| response to muramyl dipeptide (GO:0032495) | 20 | + | 0.00112 | 0.0228 |
| organic acid catabolic process (GO:0016054) | 259 | - | 0.00112 | 0.0228 |
| carboxylic acid catabolic process (GO:0046395) | 259 | - | 0.00112 | 0.0227 |
| positive regulation of interferon-gamma production (GO:0032729) | 65 | + | 0.00115 | 0.0232 |
| alpha-linolenic acid metabolic process (GO:0036109) | 13 | - | 0.00116 | 0.0234 |
| positive regulation of growth (GO:0045927) | 263 | + | 0.00116 | 0.0233 |
| regulation of Wnt signaling pathway (GO:0030111) | 350 | + | 0.00116 | 0.0234 |
| regulation of cellular localization (GO:0060341) | 889 | + | 0.00117 | 0.0235 |
| negative regulation of cellular response to growth factor stimulus (GO:0090288) | 144 | + | 0.00117 | 0.0235 |
| regulation of IRE1-mediated unfolded protein response (GO:1903894) | 13 | + | 0.00118 | 0.0237 |
| response to organonitrogen compound (GO:0010243) | 912 | + | 0.00118 | 0.0237 |
| negative regulation of cell killing (GO:0031342) | 23 | + | 0.00119 | 0.0237 |
| cellular metabolic process (GO:0044237) | 7404 | + | 0.00119 | 0.0238 |
| establishment of localization in cell (GO:0051649) | 1759 | + | 0.0012 | 0.024 |
| DNA packaging (GO:0006323) | 171 | - | 0.00121 | 0.0241 |
| sensory perception of smell (GO:0007608) | 409 | - | 0.00122 | 0.0242 |
| positive regulation of tyrosine phosphorylation of STAT protein (GO:0042531) | 67 | + | 0.00123 | 0.0244 |
| positive regulation of multi-organism process (GO:0043902) | 185 | + | 0.00123 | 0.0244 |
| protein localization to kinetochore (GO:0034501) | 12 | - | 0.00124 | 0.0246 |
| T cell proliferation (GO:0042098) | 34 | + | 0.00124 | 0.0246 |
| positive regulation of T cell migration (GO:2000406) | 30 | + | 0.00125 | 0.0248 |
| positive regulation of viral life cycle (GO:1903902) | 60 | + | 0.00126 | 0.0248 |
| purine nucleoside bisphosphate biosynthetic process (GO:0034033) | 58 | - | 0.00126 | 0.0248 |
| ribonucleoside bisphosphate biosynthetic process (GO:0034030) | 58 | - | 0.00126 | 0.0248 |
| nucleoside bisphosphate biosynthetic process (GO:0033866) | 58 | - | 0.00126 | 0.0248 |
| regulation of microtubule-based process (GO:0032886) | 219 | - | 0.00127 | 0.025 |
| endoplasmic reticulum to Golgi vesicle-mediated transport (GO:0006888) | 202 | + | 0.00129 | 0.0253 |
| positive regulation of cysteine-type endopeptidase activity involved in apoptotic signaling pathway (GO:2001269) | 11 | + | 0.0013 | 0.0254 |
| regulation of DNA endoreduplication (GO:0032875) | 9 | - | 0.00131 | 0.0256 |
| negative regulation of biosynthetic process (GO:0009890) | 1509 | + | 0.00131 | 0.0257 |
| organelle membrane fusion (GO:0090174) | 75 | + | 0.00132 | 0.0258 |
| cornification (GO:0070268) | 112 | + | 0.00132 | 0.0258 |
| positive regulation of cell population proliferation (GO:0008284) | 927 | + | 0.00133 | 0.026 |
| cellular response to virus (GO:0098586) | 31 | + | 0.00135 | 0.0264 |
| regulation of cytokinesis (GO:0032465) | 88 | - | 0.00136 | 0.0264 |
| epithelial cell differentiation (GO:0030855) | 669 | + | 0.00136 | 0.0265 |
| sensory perception of chemical stimulus (GO:0007606) | 481 | - | 0.00138 | 0.0267 |
| regulation of T cell mediated cytotoxicity (GO:0001914) | 33 | + | 0.00138 | 0.0268 |
| regulation of programmed necrotic cell death (GO:0062098) | 18 | + | 0.00139 | 0.0268 |
| coenzyme metabolic process (GO:0006732) | 243 | - | 0.00139 | 0.0268 |
| NADH metabolic process (GO:0006734) | 36 | + | 0.00141 | 0.0273 |
| positive regulation of cellular component organization (GO:0051130) | 1183 | + | 0.00143 | 0.0275 |
| glycerophospholipid biosynthetic process (GO:0046474) | 205 | - | 0.00144 | 0.0278 |
| immune response-activating cell surface receptor signaling pathway (GO:0002429) | 310 | + | 0.00146 | 0.0281 |
| response to arsenic-containing substance (GO:0046685) | 30 | + | 0.00147 | 0.0282 |
| positive regulation of protein polymerization (GO:0032273) | 128 | + | 0.0015 | 0.0287 |
| negative regulation of cellular biosynthetic process (GO:0031327) | 1485 | + | 0.0015 | 0.0287 |
| regulation of nitric oxide biosynthetic process (GO:0045428) | 58 | + | 0.0015 | 0.0287 |
| vesicle localization (GO:0051648) | 234 | + | 0.00154 | 0.0295 |
| signal transduction in absence of ligand (GO:0038034) | 31 | + | 0.00156 | 0.0298 |
| extrinsic apoptotic signaling pathway in absence of ligand (GO:0097192) | 31 | + | 0.00156 | 0.0297 |
| negative regulation of leukocyte mediated cytotoxicity (GO:0001911) | 20 | + | 0.00156 | 0.0297 |
| morphogenesis of an epithelial sheet (GO:0002011) | 48 | + | 0.00156 | 0.0297 |
| nucleoside diphosphate phosphorylation (GO:0006165) | 57 | + | 0.00158 | 0.03 |
| negative regulation of response to oxidative stress (GO:1902883) | 45 | + | 0.00158 | 0.03 |
| mitochondrial ATP synthesis coupled electron transport (GO:0042775) | 76 | + | 0.0016 | 0.0303 |
| fatty acid beta-oxidation using acyl-CoA oxidase (GO:0033540) | 15 | - | 0.00161 | 0.0306 |
| establishment of vesicle localization (GO:0051650) | 224 | + | 0.00161 | 0.0305 |
| cellular response to oxidative stress (GO:0034599) | 236 | + | 0.00162 | 0.0306 |
| negative regulation of nuclear division (GO:0051784) | 52 | - | 0.00162 | 0.0306 |
| stress-activated MAPK cascade (GO:0051403) | 105 | + | 0.00162 | 0.0306 |
| establishment of protein localization to plasma membrane (GO:0061951) | 48 | + | 0.00163 | 0.0306 |
| extracellular structure organization (GO:0043062) | 381 | + | 0.00164 | 0.0308 |
| regulation of MAP kinase activity (GO:0043405) | 332 | + | 0.00165 | 0.031 |
| regulation of chromosome organization (GO:0033044) | 343 | - | 0.00168 | 0.0315 |
| maintenance of protein location in mitochondrion (GO:0072656) | 5 | + | 0.0017 | 0.0318 |
| chromatin organization (GO:0006325) | 670 | - | 0.0017 | 0.0318 |
| negative regulation of macromolecule biosynthetic process (GO:0010558) | 1433 | + | 0.00173 | 0.0324 |
| nucleobase-containing compound metabolic process (GO:0006139) | 2667 | + | 0.00174 | 0.0325 |
| regulation of intracellular transport (GO:0032386) | 433 | + | 0.00178 | 0.0332 |
| negative regulation of viral release from host cell (GO:1902187) | 16 | + | 0.00178 | 0.0331 |
| positive regulation of nitric oxide biosynthetic process (GO:0045429) | 41 | + | 0.00179 | 0.0333 |
| positive regulation of nitric oxide metabolic process (GO:1904407) | 41 | + | 0.00179 | 0.0332 |
| negative regulation of extrinsic apoptotic signaling pathway via death domain receptors (GO:1902042) | 35 | + | 0.00189 | 0.035 |
| epiboly involved in wound healing (GO:0090505) | 24 | + | 0.0019 | 0.0352 |
| wound healing, spreading of cells (GO:0044319) | 24 | + | 0.0019 | 0.0351 |
| positive regulation of immune effector process (GO:0002699) | 213 | + | 0.00191 | 0.0352 |
| mitotic sister chromatid cohesion (GO:0007064) | 14 | - | 0.00191 | 0.0353 |
| mitotic cytokinesis (GO:0000281) | 68 | - | 0.00193 | 0.0356 |
| detection of chemical stimulus involved in sensory perception of smell (GO:0050911) | 379 | - | 0.00194 | 0.0357 |
| positive regulation of receptor signaling pathway via JAK-STAT (GO:0046427) | 84 | + | 0.00194 | 0.0357 |
| positive regulation of production of molecular mediator of immune response (GO:0002702) | 97 | + | 0.00194 | 0.0357 |
| regulation of leukocyte differentiation (GO:1902105) | 270 | + | 0.00195 | 0.0358 |
| aging (GO:0007568) | 278 | + | 0.00196 | 0.0359 |
| positive regulation of lymphocyte migration (GO:2000403) | 36 | + | 0.00201 | 0.0368 |
| regulation of actin cytoskeleton organization (GO:0032956) | 336 | + | 0.00202 | 0.0369 |
| purine nucleoside triphosphate metabolic process (GO:0009144) | 56 | + | 0.00203 | 0.0371 |
| positive regulation of protein complex assembly (GO:0031334) | 241 | + | 0.00203 | 0.037 |
| RNA transport (GO:0050658) | 183 | + | 0.00205 | 0.0374 |
| nucleic acid transport (GO:0050657) | 183 | + | 0.00205 | 0.0374 |
| regulation of trophoblast cell migration (GO:1901163) | 11 | + | 0.00206 | 0.0375 |
| regulation of adaptive immune response (GO:0002819) | 159 | + | 0.00207 | 0.0377 |
| cellular component disassembly (GO:0022411) | 386 | + | 0.00208 | 0.0377 |
| response to interleukin-18 (GO:0070673) | 10 | + | 0.00208 | 0.0377 |
| sulfur compound metabolic process (GO:0006790) | 354 | - | 0.00209 | 0.0379 |
| cellular component biogenesis (GO:0044085) | 2647 | + | 0.0021 | 0.0379 |
| kidney vasculature morphogenesis (GO:0061439) | 7 | - | 0.00211 | 0.0381 |
| renal system vasculature morphogenesis (GO:0061438) | 7 | - | 0.00211 | 0.0381 |
| regulation of interleukin-6 production (GO:0032675) | 130 | + | 0.00211 | 0.038 |
| very long-chain fatty acid metabolic process (GO:0000038) | 31 | - | 0.00212 | 0.0382 |
| actin filament bundle organization (GO:0061572) | 60 | + | 0.00218 | 0.0392 |
| regulation of multicellular organismal development (GO:2000026) | 1997 | + | 0.00221 | 0.0397 |
| negative regulation of cytokine production (GO:0001818) | 263 | + | 0.00221 | 0.0397 |
| response to host defenses (GO:0052200) | 7 | + | 0.00221 | 0.0397 |
| response to defenses of other organism involved in symbiotic interaction (GO:0052173) | 7 | + | 0.00221 | 0.0396 |
| response to host (GO:0075136) | 7 | + | 0.00221 | 0.0396 |
| nucleoside diphosphate metabolic process (GO:0009132) | 79 | + | 0.00222 | 0.0396 |
| mRNA splice site selection (GO:0006376) | 30 | + | 0.00222 | 0.0396 |
| cellular response to chemokine (GO:1990869) | 88 | + | 0.00223 | 0.0397 |
| response to chemokine (GO:1990868) | 88 | + | 0.00223 | 0.0397 |
| tissue development (GO:0009888) | 1721 | + | 0.00228 | 0.0405 |
| cellular response to arsenic-containing substance (GO:0071243) | 18 | + | 0.00232 | 0.0412 |
| regulation of chemokine biosynthetic process (GO:0045073) | 14 | + | 0.00233 | 0.0414 |
| antimicrobial humoral immune response mediated by antimicrobial peptide (GO:0061844) | 65 | + | 0.00235 | 0.0416 |
| regulation of T cell mediated immunity (GO:0002709) | 68 | + | 0.00238 | 0.0421 |
| negative regulation of protein kinase activity by regulation of protein phosphorylation (GO:0044387) | 8 | + | 0.00239 | 0.0422 |
| positive regulation of cell growth (GO:0030307) | 162 | + | 0.00239 | 0.0422 |
| endosomal transport (GO:0016197) | 209 | + | 0.00239 | 0.0422 |
| regulation of hematopoietic stem cell differentiation (GO:1902036) | 71 | + | 0.00239 | 0.0422 |
| regulation of calcidiol 1-monooxygenase activity (GO:0060558) | 7 | + | 0.00241 | 0.0424 |
| negative regulation of I-kappaB kinase/NF-kappaB signaling (GO:0043124) | 43 | + | 0.00241 | 0.0424 |
| negative regulation of protein phosphorylation (GO:0001933) | 393 | + | 0.00243 | 0.0427 |
| regulation of tyrosine phosphorylation of STAT protein (GO:0042509) | 77 | + | 0.00244 | 0.0428 |
| ciliary basal body organization (GO:0032053) | 4 | - | 0.00244 | 0.0428 |
| regulation of ERK1 and ERK2 cascade (GO:0070372) | 287 | + | 0.00244 | 0.0428 |
| regulation of protein complex assembly (GO:0043254) | 434 | + | 0.00244 | 0.0427 |
| macroautophagy (GO:0016236) | 151 | + | 0.00246 | 0.0429 |
| mitotic spindle elongation (GO:0000022) | 8 | - | 0.00249 | 0.0434 |
| maternal placenta development (GO:0001893) | 35 | + | 0.00249 | 0.0434 |
| phosphatidic acid biosynthetic process (GO:0006654) | 42 | - | 0.00252 | 0.0438 |
| detection of stimulus (GO:0051606) | 638 | - | 0.00254 | 0.0442 |
| response to reactive oxygen species (GO:0000302) | 188 | + | 0.00255 | 0.0442 |
| T cell migration (GO:0072678) | 24 | + | 0.00256 | 0.0445 |
| regulation of viral release from host cell (GO:1902186) | 32 | + | 0.0026 | 0.045 |
| epiboly (GO:0090504) | 25 | + | 0.00261 | 0.0451 |
| phosphatidic acid metabolic process (GO:0046473) | 43 | - | 0.00261 | 0.0451 |
| negative regulation of lipid storage (GO:0010888) | 19 | + | 0.00262 | 0.0452 |
| decidualization (GO:0046697) | 24 | + | 0.00263 | 0.0453 |
| response to vitamin D (GO:0033280) | 31 | + | 0.00263 | 0.0454 |
| positive regulation of neuron projection development (GO:0010976) | 272 | + | 0.00264 | 0.0454 |
| positive regulation of angiogenesis (GO:0045766) | 164 | + | 0.00264 | 0.0454 |
| immune response-regulating cell surface receptor signaling pathway (GO:0002768) | 345 | + | 0.00266 | 0.0457 |
| actin filament-based process (GO:0030029) | 548 | + | 0.00266 | 0.0457 |
| regulation of centriole replication (GO:0046599) | 22 | - | 0.00268 | 0.046 |
| steroid metabolic process (GO:0008202) | 251 | - | 0.00268 | 0.0459 |
| fatty-acyl-CoA metabolic process (GO:0035337) | 40 | - | 0.0027 | 0.0462 |
| regulation of transmembrane receptor protein serine/threonine kinase signaling pathway (GO:0090092) | 227 | + | 0.00271 | 0.0462 |
| protein localization to organelle (GO:0033365) | 702 | + | 0.00271 | 0.0463 |
| positive regulation of leukocyte differentiation (GO:1902107) | 144 | + | 0.00276 | 0.047 |
| positive regulation of cell projection organization (GO:0031346) | 373 | + | 0.00276 | 0.047 |
| regulation of mitotic centrosome separation (GO:0046602) | 10 | - | 0.00276 | 0.047 |
| oxidative phosphorylation (GO:0006119) | 93 | + | 0.00277 | 0.0471 |
| cellular response to transforming growth factor beta stimulus (GO:0071560) | 152 | + | 0.00277 | 0.0471 |
| anion homeostasis (GO:0055081) | 58 | - | 0.00281 | 0.0476 |
| regulation of DNA duplex unwinding (GO:1905462) | 5 | - | 0.00282 | 0.0478 |
| regulation of protein exit from endoplasmic reticulum (GO:0070861) | 25 | + | 0.00284 | 0.0481 |
| TRAIL-activated apoptotic signaling pathway (GO:0036462) | 5 | + | 0.00285 | 0.0482 |
| regulation of intrinsic apoptotic signaling pathway in response to osmotic stress (GO:1902218) | 6 | + | 0.00287 | 0.0485 |
| regulation of cellular response to osmotic stress (GO:0106049) | 6 | + | 0.00287 | 0.0485 |
| regulation of leukocyte adhesion to vascular endothelial cell (GO:1904994) | 17 | + | 0.00288 | 0.0484 |
| positive regulation of T cell apoptotic process (GO:0070234) | 15 | + | 0.00288 | 0.0484 |
| actin filament bundle assembly (GO:0051017) | 58 | + | 0.00288 | 0.0485 |
| positive regulation of T cell differentiation (GO:0045582) | 81 | + | 0.00289 | 0.0485 |
| vesicle fusion (GO:0006906) | 69 | + | 0.00291 | 0.0488 |
| positive regulation of adaptive immune response (GO:0002821) | 102 | + | 0.00292 | 0.0489 |
| leukocyte differentiation (GO:0002521) | 325 | + | 0.00292 | 0.0488 |
| nuclear envelope disassembly (GO:0051081) | 16 | - | 0.00293 | 0.049 |
| membrane disassembly (GO:0030397) | 16 | - | 0.00293 | 0.049 |
| neurotrophin signaling pathway (GO:0038179) | 28 | + | 0.00295 | 0.0492 |
| positive regulation of T cell mediated cytotoxicity (GO:0001916) | 26 | + | 0.00298 | 0.0497 |
| negative regulation of hydrolase activity (GO:0051346) | 448 | + | 0.003 | 0.0499 |

**Supplementary Table 2**

| **Analysis Type:** | **PANTHER Enrichment Test (release 20190701)** |  |  |  |
| --- | --- | --- | --- | --- |
| **Annotation Version and Release Date:** | **GO Ontology database Released 2019-02-02** |  |  |  |
| **Analyzed List:** | **H661_GFold.txt (Homo sapiens)** |  |  |  |
| **Correction:** | **FDR** |  |  |  |
| **GO biological process complete** | **number** | **Over/Under** | **p-value** | **fdr** |
| organelle organization (GO:0006996) | 3262 | - | 0 | 0 |
| cellular response to DNA damage stimulus (GO:0006974) | 749 | - | 0 | 0 |
| regulation of mitotic cell cycle (GO:0007346) | 638 | - | 0 | 0 |
| mitotic cell cycle process (GO:1903047) | 575 | - | 0 | 0 |
| cellular aromatic compound metabolic process (GO:0006725) | 2883 | - | 0 | 0 |
| organic substance metabolic process (GO:0071704) | 7612 | - | 0 | 0 |
| regulation of mitotic cell cycle phase transition (GO:1901990) | 412 | - | 0 | 0 |
| regulation of cell cycle phase transition (GO:1901987) | 441 | - | 0 | 0 |
| cell cycle (GO:0007049) | 1298 | - | 0 | 0 |
| mitotic cell cycle phase transition (GO:0044772) | 260 | - | 0 | 0 |
| cell cycle phase transition (GO:0044770) | 268 | - | 0 | 0 |
| mRNA processing (GO:0006397) | 454 | - | 0 | 0 |
| RNA processing (GO:0006396) | 837 | - | 0 | 0 |
| regulation of cell cycle (GO:0051726) | 1174 | - | 0 | 0 |
| regulation of chromosome organization (GO:0033044) | 343 | - | 0 | 0 |
| nucleic acid metabolic process (GO:0090304) | 2180 | - | 0 | 0 |
| nucleobase-containing compound metabolic process (GO:0006139) | 2667 | - | 0 | 0 |
| mitotic nuclear division (GO:0140014) | 137 | - | 0 | 0 |
| organic cyclic compound metabolic process (GO:1901360) | 3107 | - | 0 | 0 |
| cellular macromolecule metabolic process (GO:0044260) | 4924 | - | 0 | 0 |
| primary metabolic process (GO:0044238) | 7270 | - | 0 | 0 |
| cellular metabolic process (GO:0044237) | 7404 | - | 0 | 0 |
| cell division (GO:0051301) | 480 | - | 0 | 0 |
| chromosome organization (GO:0051276) | 1014 | - | 0 | 0 |
| mRNA splicing, via spliceosome (GO:0000398) | 284 | - | 0 | 0 |
| RNA splicing, via transesterification reactions with bulged adenosine as nucleophile (GO:0000377) | 284 | - | 0 | 0 |
| RNA splicing, via transesterification reactions (GO:0000375) | 287 | - | 0 | 0 |
| mitotic cell cycle (GO:0000278) | 670 | - | 0 | 0 |
| macromolecule metabolic process (GO:0043170) | 5990 | - | 0 | 0 |
| RNA metabolic process (GO:0016070) | 1602 | - | 0 | 0 |
| cellular nitrogen compound metabolic process (GO:0034641) | 3283 | - | 0 | 0 |
| RNA splicing (GO:0008380) | 382 | - | 0 | 0 |
| ribonucleoprotein complex biogenesis (GO:0022613) | 428 | - | 0 | 0 |
| posttranscriptional regulation of gene expression (GO:0010608) | 517 | - | 0 | 0 |
| regulation of cell cycle process (GO:0010564) | 759 | - | 0 | 0 |
| heterocycle metabolic process (GO:0046483) | 2841 | - | 0 | 0 |
| gene expression (GO:0010467) | 1940 | - | 0 | 0 |
| cell cycle process (GO:0022402) | 946 | - | 0 | 0 |
| regulation of mRNA metabolic process (GO:1903311) | 323 | - | 0 | 0 |
| nitrogen compound metabolic process (GO:0006807) | 6778 | - | 0 | 0 |
| DNA metabolic process (GO:0006259) | 713 | - | 0 | 0 |
| regulation of cell cycle G2/M phase transition (GO:1902749) | 210 | - | 0 | 0 |
| RNA localization (GO:0006403) | 204 | - | 0 | 0 |
| protein-containing complex subunit organization (GO:0043933) | 1790 | - | 0 | 0 |
| cellular response to stress (GO:0033554) | 1642 | - | 0 | 0 |
| negative regulation of cell cycle (GO:0045786) | 574 | - | 0 | 0 |
| negative regulation of cell cycle process (GO:0010948) | 323 | - | 0 | 0 |
| microtubule cytoskeleton organization involved in mitosis (GO:1902850) | 93 | - | 0 | 0 |
| cellular component biogenesis (GO:0044085) | 2647 | - | 0 | 0 |
| ncRNA metabolic process (GO:0034660) | 455 | - | 0 | 0 |
| cellular component organization or biogenesis (GO:0071840) | 5576 | - | 0 | 0 |
| regulation of G2/M transition of mitotic cell cycle (GO:0010389) | 195 | - | 0 | 0 |
| regulation of mRNA catabolic process (GO:0061013) | 200 | - | 0 | 0 |
| mRNA metabolic process (GO:0016071) | 662 | - | 0 | 0 |
| regulation of organelle organization (GO:0033043) | 1268 | - | 0 | 0 |
| regulation of RNA stability (GO:0043487) | 184 | - | 0 | 0 |
| metabolic process (GO:0008152) | 8128 | - | 0 | 0 |
| organelle fission (GO:0048285) | 296 | - | 0 | 0 |
| mitotic sister chromatid segregation (GO:0000070) | 104 | - | 0 | 0 |
| sister chromatid segregation (GO:0000819) | 136 | - | 0 | 0 |
| nuclear transport (GO:0051169) | 252 | - | 0 | 0 |
| regulation of mRNA stability (GO:0043488) | 178 | - | 0 | 0 |
| nucleocytoplasmic transport (GO:0006913) | 249 | - | 0 | 0 |
| chromosome segregation (GO:0007059) | 260 | - | 0 | 0 |
| negative regulation of cell cycle phase transition (GO:1901988) | 228 | - | 0 | 0 |
| nuclear export (GO:0051168) | 156 | - | 0 | 0 |
| negative regulation of mitotic cell cycle phase transition (GO:1901991) | 215 | - | 0 | 0 |
| RNA transport (GO:0050658) | 183 | - | 0 | 0 |
| nucleic acid transport (GO:0050657) | 183 | - | 0 | 0 |
| nuclear division (GO:0000280) | 270 | - | 0 | 0 |
| establishment of RNA localization (GO:0051236) | 185 | - | 0 | 0 |
| protein export from nucleus (GO:0006611) | 147 | - | 0 | 0 |
| RNA export from nucleus (GO:0006405) | 127 | - | 0 | 0 |
| spindle organization (GO:0007051) | 135 | - | 0 | 0 |
| nucleobase-containing compound transport (GO:0015931) | 227 | - | 0 | 0 |
| ncRNA processing (GO:0034470) | 372 | - | 0 | 0 |
| Unclassified (UNCLASSIFIED) | 2483 | + | 0 | 0.000000000111 |
| regulation of nucleobase-containing compound metabolic process (GO:0019219) | 3977 | - | 0 | 0.000000000113 |
| cellular nitrogen compound biosynthetic process (GO:0044271) | 1571 | - | 0 | 0.000000000116 |
| regulation of chromosome segregation (GO:0051983) | 105 | - | 0 | 0.000000000136 |
| cellular component organization (GO:0016043) | 5400 | - | 0 | 0.000000000255 |
| ribonucleoprotein complex localization (GO:0071166) | 125 | - | 0 | 0.000000000281 |
| negative regulation of mitotic cell cycle (GO:0045930) | 304 | - | 0 | 0.000000000323 |
| regulation of catabolic process (GO:0009894) | 966 | - | 0 | 0.000000000327 |
| SRP-dependent cotranslational protein targeting to membrane (GO:0006614) | 96 | + | 0 | 0.000000000371 |
| ribonucleoprotein complex export from nucleus (GO:0071426) | 124 | - | 0 | 0.000000000488 |
| mitotic spindle organization (GO:0007052) | 70 | - | 0 | 0.000000000589 |
| nucleobase-containing compound biosynthetic process (GO:0034654) | 1042 | - | 0 | 0.000000000718 |
| organic cyclic compound biosynthetic process (GO:1901362) | 1252 | - | 0 | 0.00000000083 |
| regulation of DNA metabolic process (GO:0051052) | 336 | - | 0 | 0.00000000086 |
| ribosome biogenesis (GO:0042254) | 279 | - | 0 | 0.00000000104 |
| protein-containing complex assembly (GO:0065003) | 1511 | - | 0 | 0.00000000103 |
| heterocycle biosynthetic process (GO:0018130) | 1108 | - | 0 | 0.00000000151 |
| mRNA transport (GO:0051028) | 146 | - | 0 | 0.00000000167 |
| nuclear chromosome segregation (GO:0098813) | 210 | - | 0 | 0.00000000192 |
| positive regulation of chromosome organization (GO:2001252) | 171 | - | 0 | 0.00000000226 |
| DNA repair (GO:0006281) | 484 | - | 0 | 0.00000000227 |
| regulation of mitotic sister chromatid segregation (GO:0033047) | 71 | - | 0 | 0.00000000283 |
| G2/M transition of mitotic cell cycle (GO:0000086) | 135 | - | 0 | 0.0000000029 |
| organic substance biosynthetic process (GO:1901576) | 2767 | - | 0 | 0.00000000314 |
| protein targeting to ER (GO:0045047) | 109 | + | 0 | 0.00000000311 |
| regulation of primary metabolic process (GO:0080090) | 5882 | - | 0 | 0.00000000327 |
| establishment of protein localization to endoplasmic reticulum (GO:0072599) | 113 | + | 0 | 0.00000000435 |
| cellular macromolecule biosynthetic process (GO:0034645) | 1658 | - | 0 | 0.00000000436 |
| cell cycle G2/M phase transition (GO:0044839) | 137 | - | 0 | 0.00000000438 |
| aromatic compound biosynthetic process (GO:0019438) | 1119 | - | 0 | 0.0000000047 |
| cell cycle checkpoint (GO:0000075) | 193 | - | 0 | 0.00000000466 |
| cotranslational protein targeting to membrane (GO:0006613) | 100 | + | 0 | 0.00000000479 |
| regulation of sister chromatid segregation (GO:0033045) | 83 | - | 0 | 0.00000000514 |
| regulation of cellular metabolic process (GO:0031323) | 6075 | - | 0 | 0.0000000064 |
| regulation of mitotic nuclear division (GO:0007088) | 189 | - | 0 | 0.00000000668 |
| regulation of telomere maintenance (GO:0032204) | 82 | - | 0 | 0.00000000761 |
| biosynthetic process (GO:0009058) | 2825 | - | 0 | 0.0000000083 |
| cellular protein-containing complex assembly (GO:0034622) | 797 | - | 0 | 0.0000000092 |
| mitochondrial gene expression (GO:0140053) | 137 | - | 0 | 0.0000000112 |
| regulation of mRNA processing (GO:0050684) | 135 | - | 0 | 0.0000000116 |
| cellular component assembly (GO:0022607) | 2432 | - | 0.00000000016 | 0.0000000217 |
| cellular biosynthetic process (GO:0044249) | 2650 | - | 0.000000000177 | 0.0000000237 |
| microtubule cytoskeleton organization (GO:0000226) | 450 | - | 0.000000000186 | 0.0000000247 |
| regulation of nitrogen compound metabolic process (GO:0051171) | 5705 | - | 0.000000000197 | 0.0000000259 |
| positive regulation of cell cycle (GO:0045787) | 396 | - | 0.000000000253 | 0.000000033 |
| regulation of cellular macromolecule biosynthetic process (GO:2000112) | 3863 | - | 0.00000000026 | 0.0000000337 |
| regulation of translation (GO:0006417) | 346 | - | 0.000000000271 | 0.0000000349 |
| regulation of gene silencing (GO:0060968) | 126 | - | 0.00000000029 | 0.0000000369 |
| protein sumoylation (GO:0016925) | 64 | - | 0.000000000323 | 0.0000000408 |
| DNA conformation change (GO:0071103) | 266 | - | 0.000000000326 | 0.0000000409 |
| ncRNA export from nucleus (GO:0097064) | 39 | - | 0.000000000357 | 0.0000000444 |
| tRNA-containing ribonucleoprotein complex export from nucleus (GO:0071431) | 34 | - | 0.000000000449 | 0.0000000555 |
| tRNA export from nucleus (GO:0006409) | 34 | - | 0.000000000449 | 0.000000055 |
| mRNA export from nucleus (GO:0006406) | 107 | - | 0.000000000555 | 0.0000000675 |
| mRNA-containing ribonucleoprotein complex export from nucleus (GO:0071427) | 107 | - | 0.000000000555 | 0.0000000669 |
| regulation of biosynthetic process (GO:0009889) | 4191 | - | 0.000000000623 | 0.0000000746 |
| ribonucleoprotein complex subunit organization (GO:0071826) | 233 | - | 0.000000000646 | 0.0000000767 |
| microtubule-based process (GO:0007017) | 636 | - | 0.00000000069 | 0.0000000814 |
| regulation of RNA metabolic process (GO:0051252) | 3740 | - | 0.000000000716 | 0.0000000838 |
| macromolecule biosynthetic process (GO:0009059) | 1712 | - | 0.000000000749 | 0.0000000871 |
| cell cycle G1/S phase transition (GO:0044843) | 119 | - | 0.000000000784 | 0.0000000904 |
| RNA biosynthetic process (GO:0032774) | 634 | - | 0.000000000963 | 0.00000011 |
| positive regulation of cell cycle process (GO:0090068) | 287 | - | 0.00000000097 | 0.00000011 |
| regulation of macromolecule biosynthetic process (GO:0010556) | 3988 | - | 0.00000000104 | 0.000000117 |
| regulation of cellular catabolic process (GO:0031329) | 807 | - | 0.00000000113 | 0.000000127 |
| chromatin organization (GO:0006325) | 670 | - | 0.00000000117 | 0.000000131 |
| regulation of cellular response to stress (GO:0080135) | 696 | - | 0.00000000122 | 0.000000135 |
| regulation of mitotic sister chromatid separation (GO:0010965) | 60 | - | 0.00000000125 | 0.000000137 |
| negative regulation of mRNA metabolic process (GO:1903312) | 81 | - | 0.00000000134 | 0.000000146 |
| organelle localization (GO:0051640) | 589 | - | 0.00000000142 | 0.000000154 |
| nucleic acid-templated transcription (GO:0097659) | 623 | - | 0.00000000162 | 0.000000174 |
| proteasomal protein catabolic process (GO:0010498) | 332 | - | 0.00000000164 | 0.000000175 |
| regulation of cellular response to heat (GO:1900034) | 79 | - | 0.00000000168 | 0.000000178 |
| anaphase-promoting complex-dependent catabolic process (GO:0031145) | 82 | - | 0.0000000017 | 0.000000179 |
| tRNA transport (GO:0051031) | 36 | - | 0.00000000173 | 0.000000181 |
| G1/S transition of mitotic cell cycle (GO:0000082) | 118 | - | 0.00000000178 | 0.000000185 |
| regulation of cellular biosynthetic process (GO:0031326) | 4116 | - | 0.00000000199 | 0.000000205 |
| transcription, DNA-templated (GO:0006351) | 622 | - | 0.0000000021 | 0.000000216 |
| DNA replication (GO:0006260) | 211 | - | 0.00000000214 | 0.000000218 |
| regulation of metabolic process (GO:0019222) | 6522 | - | 0.00000000235 | 0.000000238 |
| protein-containing complex disassembly (GO:0032984) | 229 | - | 0.00000000245 | 0.000000247 |
| mitotic cell cycle checkpoint (GO:0007093) | 156 | - | 0.00000000245 | 0.000000245 |
| protein-containing complex localization (GO:0031503) | 252 | - | 0.00000000288 | 0.000000286 |
| regulation of nuclear division (GO:0051783) | 213 | - | 0.00000000299 | 0.000000296 |
| negative regulation of chromosome organization (GO:2001251) | 136 | - | 0.00000000358 | 0.000000351 |
| ribonucleoprotein complex assembly (GO:0022618) | 219 | - | 0.00000000424 | 0.000000414 |
| regulation of macromolecule metabolic process (GO:0060255) | 6010 | - | 0.00000000443 | 0.000000429 |
| negative regulation of RNA catabolic process (GO:1902369) | 59 | - | 0.00000000445 | 0.000000429 |
| cellular protein catabolic process (GO:0044257) | 588 | - | 0.00000000504 | 0.000000483 |
| cellular protein complex disassembly (GO:0043624) | 138 | - | 0.00000000532 | 0.000000506 |
| cellular localization (GO:0051641) | 2354 | - | 0.00000000751 | 0.000000711 |
| regulation of chromosome separation (GO:1905818) | 65 | - | 0.00000000779 | 0.000000733 |
| modification-dependent macromolecule catabolic process (GO:0043632) | 516 | - | 0.00000000879 | 0.000000822 |
| proteasome-mediated ubiquitin-dependent protein catabolic process (GO:0043161) | 308 | - | 0.00000000926 | 0.000000861 |
| positive regulation of organelle organization (GO:0010638) | 612 | - | 0.00000000937 | 0.000000866 |
| regulation of cellular component organization (GO:0051128) | 2423 | - | 0.0000000111 | 0.00000102 |
| ubiquitin-dependent protein catabolic process (GO:0006511) | 499 | - | 0.0000000165 | 0.0000015 |
| rRNA metabolic process (GO:0016072) | 211 | - | 0.0000000169 | 0.00000153 |
| regulation of posttranscriptional gene silencing (GO:0060147) | 88 | - | 0.0000000184 | 0.00000166 |
| regulation of gene silencing by RNA (GO:0060966) | 88 | - | 0.0000000184 | 0.00000165 |
| regulation of gene expression (GO:0010468) | 4373 | - | 0.0000000184 | 0.00000165 |
| negative regulation of nucleobase-containing compound metabolic process (GO:0045934) | 1393 | - | 0.0000000199 | 0.00000177 |
| regulation of mitotic metaphase/anaphase transition (GO:0030071) | 54 | - | 0.0000000213 | 0.00000188 |
| modification-dependent protein catabolic process (GO:0019941) | 505 | - | 0.0000000224 | 0.00000196 |
| regulation of telomerase RNA localization to Cajal body (GO:1904872) | 18 | - | 0.0000000254 | 0.00000221 |
| positive regulation of telomere maintenance (GO:0032206) | 50 | - | 0.0000000303 | 0.00000263 |
| rRNA processing (GO:0006364) | 201 | - | 0.0000000315 | 0.00000272 |
| proteolysis involved in cellular protein catabolic process (GO:0051603) | 560 | - | 0.0000000329 | 0.00000282 |
| tRNA metabolic process (GO:0006399) | 182 | - | 0.0000000352 | 0.00000301 |
| protein catabolic process (GO:0030163) | 651 | - | 0.0000000383 | 0.00000325 |
| protein localization to nucleus (GO:0034504) | 152 | - | 0.0000000396 | 0.00000334 |
| negative regulation of cell cycle G2/M phase transition (GO:1902750) | 98 | - | 0.0000000409 | 0.00000344 |
| regulation of telomere maintenance via telomere lengthening (GO:1904356) | 63 | - | 0.0000000417 | 0.00000349 |
| mitochondrial translation (GO:0032543) | 112 | - | 0.0000000549 | 0.00000457 |
| cellular macromolecule localization (GO:0070727) | 1542 | - | 0.0000000565 | 0.00000467 |
| mitochondrial translational elongation (GO:0070125) | 88 | - | 0.0000000634 | 0.00000522 |
| regulation of gene silencing by miRNA (GO:0060964) | 86 | - | 0.0000000714 | 0.00000585 |
| chromosome localization (GO:0050000) | 72 | - | 0.0000000778 | 0.00000633 |
| DNA integrity checkpoint (GO:0031570) | 143 | - | 0.000000083 | 0.00000673 |
| cellular protein localization (GO:0034613) | 1535 | - | 0.0000000882 | 0.00000711 |
| cellular protein metabolic process (GO:0044267) | 3626 | - | 0.0000000929 | 0.00000745 |
| positive regulation of DNA metabolic process (GO:0051054) | 188 | - | 0.0000000952 | 0.0000076 |
| nuclear-transcribed mRNA catabolic process, nonsense-mediated decay (GO:0000184) | 119 | + | 0.0000000988 | 0.00000785 |
| positive regulation of nucleobase-containing compound metabolic process (GO:0045935) | 1837 | - | 0.000000111 | 0.00000876 |
| extracellular matrix organization (GO:0030198) | 335 | + | 0.000000115 | 0.00000904 |
| regulation of cellular amide metabolic process (GO:0034248) | 391 | - | 0.000000116 | 0.0000091 |
| regulation of metaphase/anaphase transition of cell cycle (GO:1902099) | 56 | - | 0.000000129 | 0.00001 |
| translational elongation (GO:0006414) | 124 | - | 0.00000013 | 0.00001 |
| negative regulation of RNA metabolic process (GO:0051253) | 1300 | - | 0.000000158 | 0.0000122 |
| establishment of chromosome localization (GO:0051303) | 71 | - | 0.000000174 | 0.0000133 |
| regulation of RNA splicing (GO:0043484) | 134 | - | 0.000000195 | 0.0000149 |
| metaphase plate congression (GO:0051310) | 53 | - | 0.000000205 | 0.0000156 |
| positive regulation of establishment of protein localization to telomere (GO:1904851) | 10 | - | 0.000000216 | 0.0000163 |
| macromolecule modification (GO:0043412) | 3227 | - | 0.00000026 | 0.0000195 |
| translational termination (GO:0006415) | 97 | - | 0.000000294 | 0.0000221 |
| regulation of cellular protein localization (GO:1903827) | 526 | - | 0.000000301 | 0.0000225 |
| positive regulation of telomere maintenance via telomere lengthening (GO:1904358) | 37 | - | 0.000000345 | 0.0000256 |
| regulation of telomere maintenance via telomerase (GO:0032210) | 55 | - | 0.000000353 | 0.0000261 |
| nucleus organization (GO:0006997) | 129 | - | 0.000000383 | 0.0000282 |
| positive regulation of telomerase RNA localization to Cajal body (GO:1904874) | 15 | - | 0.000000385 | 0.0000281 |
| protein localization to chromosome (GO:0034502) | 63 | - | 0.000000403 | 0.0000294 |
| regulation of response to DNA damage stimulus (GO:2001020) | 207 | - | 0.000000435 | 0.0000315 |
| negative regulation of G2/M transition of mitotic cell cycle (GO:0010972) | 88 | - | 0.000000467 | 0.0000337 |
| establishment of organelle localization (GO:0051656) | 396 | - | 0.000000469 | 0.0000337 |
| protein localization to endoplasmic reticulum (GO:0070972) | 136 | + | 0.00000048 | 0.0000343 |
| mitochondrial translational termination (GO:0070126) | 89 | - | 0.000000494 | 0.0000351 |
| regulation of mRNA splicing, via spliceosome (GO:0048024) | 98 | - | 0.000000625 | 0.0000443 |
| negative regulation of sister chromatid segregation (GO:0033046) | 35 | - | 0.000000648 | 0.0000457 |
| chromatin remodeling (GO:0006338) | 160 | - | 0.000000655 | 0.000046 |
| nuclear pore organization (GO:0006999) | 14 | - | 0.000000696 | 0.0000486 |
| cellular macromolecule catabolic process (GO:0044265) | 861 | - | 0.000000697 | 0.0000486 |
| regulation of protein localization to chromosome, telomeric region (GO:1904814) | 15 | - | 0.000000728 | 0.0000504 |
| protein modification by small protein conjugation or removal (GO:0070647) | 954 | - | 0.000000769 | 0.0000531 |
| mitochondrial membrane organization (GO:0007006) | 119 | - | 0.000000831 | 0.0000571 |
| extracellular structure organization (GO:0043062) | 381 | + | 0.000000848 | 0.000058 |
| mitotic metaphase plate congression (GO:0007080) | 43 | - | 0.000000862 | 0.0000587 |
| multi-organism localization (GO:1902579) | 68 | - | 0.000000863 | 0.0000585 |
| multi-organism transport (GO:0044766) | 68 | - | 0.000000863 | 0.0000583 |
| Wnt signaling pathway, planar cell polarity pathway (GO:0060071) | 97 | - | 0.000000887 | 0.0000597 |
| regulation of establishment of protein localization to telomere (GO:0070203) | 11 | - | 0.000000916 | 0.0000613 |
| positive regulation of protein localization to chromosome, telomeric region (GO:1904816) | 13 | - | 0.000000961 | 0.0000641 |
| positive regulation of protein localization to Cajal body (GO:1904871) | 9 | - | 0.000000967 | 0.0000642 |
| regulation of protein localization to Cajal body (GO:1904869) | 9 | - | 0.000000967 | 0.0000639 |
| positive regulation of cellular component organization (GO:0051130) | 1183 | - | 0.000000996 | 0.0000656 |
| negative regulation of mitotic sister chromatid separation (GO:2000816) | 30 | - | 0.000000999 | 0.0000655 |
| negative regulation of chromosome segregation (GO:0051985) | 36 | - | 0.00000102 | 0.0000667 |
| negative regulation of mitotic metaphase/anaphase transition (GO:0045841) | 27 | - | 0.00000104 | 0.0000677 |
| RNA stabilization (GO:0043489) | 48 | - | 0.00000107 | 0.0000693 |
| negative regulation of mitotic sister chromatid segregation (GO:0033048) | 33 | - | 0.00000108 | 0.0000694 |
| negative regulation of mRNA catabolic process (GO:1902373) | 52 | - | 0.00000109 | 0.00007 |
| system process (GO:0003008) | 1869 | + | 0.0000011 | 0.0000706 |
| regulation of nucleic acid-templated transcription (GO:1903506) | 3489 | - | 0.0000013 | 0.000083 |
| regulation of RNA biosynthetic process (GO:2001141) | 3497 | - | 0.0000013 | 0.0000827 |
| positive regulation of macromolecule biosynthetic process (GO:0010557) | 1855 | - | 0.00000134 | 0.0000846 |
| import into nucleus (GO:0051170) | 105 | - | 0.00000137 | 0.0000864 |
| pore complex assembly (GO:0046931) | 15 | - | 0.00000138 | 0.0000868 |
| cytoskeleton-dependent cytokinesis (GO:0061640) | 95 | - | 0.00000144 | 0.0000902 |
| positive regulation of metabolic process (GO:0009893) | 3527 | - | 0.00000151 | 0.0000942 |
| negative regulation of organelle organization (GO:0010639) | 369 | - | 0.00000154 | 0.0000955 |
| positive regulation of cellular biosynthetic process (GO:0031328) | 1941 | - | 0.0000016 | 0.0000991 |
| positive regulation of cellular metabolic process (GO:0031325) | 3236 | - | 0.00000162 | 0.0000993 |
| DNA replication-independent nucleosome organization (GO:0034724) | 40 | - | 0.00000163 | 0.0000997 |
| positive regulation of macromolecule metabolic process (GO:0010604) | 3259 | - | 0.00000163 | 0.0000995 |
| negative regulation of chromosome separation (GO:1905819) | 31 | - | 0.00000165 | 0.000101 |
| intracellular transport of virus (GO:0075733) | 52 | - | 0.00000166 | 0.0001 |
| negative regulation of metaphase/anaphase transition of cell cycle (GO:1902100) | 28 | - | 0.00000181 | 0.000109 |
| positive regulation of telomere maintenance via telomerase (GO:0032212) | 34 | - | 0.00000195 | 0.000117 |
| regulation of transcription, DNA-templated (GO:0006355) | 3433 | - | 0.00000201 | 0.00012 |
| cytokinesis (GO:0000910) | 97 | - | 0.00000204 | 0.000121 |
| spliceosomal snRNP assembly (GO:0000387) | 37 | - | 0.00000212 | 0.000126 |
| establishment of localization in cell (GO:0051649) | 1759 | - | 0.00000218 | 0.000129 |
| cytoskeleton organization (GO:0007010) | 1049 | - | 0.00000224 | 0.000132 |
| positive regulation of protein insertion into mitochondrial membrane involved in apoptotic signaling pathway (GO:1900740) | 27 | - | 0.00000232 | 0.000137 |
| regulation of protein insertion into mitochondrial membrane involved in apoptotic signaling pathway (GO:1900739) | 27 | - | 0.00000232 | 0.000136 |
| DNA damage checkpoint (GO:0000077) | 131 | - | 0.00000233 | 0.000136 |
| mitochondrial transport (GO:0006839) | 203 | - | 0.00000236 | 0.000137 |
| mitochondrial RNA metabolic process (GO:0000959) | 37 | - | 0.00000236 | 0.000137 |
| membrane organization (GO:0061024) | 748 | - | 0.00000251 | 0.000145 |
| regulation of gene expression, epigenetic (GO:0040029) | 232 | - | 0.00000251 | 0.000144 |
| membrane docking (GO:0022406) | 177 | - | 0.00000259 | 0.000148 |
| retrograde vesicle-mediated transport, Golgi to endoplasmic reticulum (GO:0006890) | 83 | - | 0.00000265 | 0.000151 |
| antigen processing and presentation of exogenous antigen (GO:0019884) | 183 | - | 0.00000287 | 0.000163 |
| regulation of establishment of protein localization to chromosome (GO:0070202) | 12 | - | 0.00000306 | 0.000173 |
| Fc receptor signaling pathway (GO:0038093) | 176 | - | 0.00000307 | 0.000173 |
| spindle assembly (GO:0051225) | 80 | - | 0.00000308 | 0.000173 |
| ciliary basal body-plasma membrane docking (GO:0097711) | 95 | - | 0.0000031 | 0.000174 |
| positive regulation of biosynthetic process (GO:0009891) | 1973 | - | 0.00000314 | 0.000175 |
| positive regulation of chromosome segregation (GO:0051984) | 29 | - | 0.0000034 | 0.000189 |
| establishment of mitotic spindle localization (GO:0040001) | 27 | - | 0.00000351 | 0.000194 |
| positive regulation of mitotic cell cycle (GO:0045931) | 154 | - | 0.00000363 | 0.0002 |
| mRNA 3-end processing (GO:0031124) | 81 | - | 0.00000368 | 0.000203 |
| positive regulation of mRNA processing (GO:0050685) | 31 | - | 0.00000368 | 0.000202 |
| positive regulation of mRNA metabolic process (GO:1903313) | 77 | - | 0.00000381 | 0.000208 |
| protein polyubiquitination (GO:0000209) | 296 | - | 0.00000382 | 0.000208 |
| regulation of DNA repair (GO:0006282) | 114 | - | 0.0000039 | 0.000212 |
| transport of virus (GO:0046794) | 55 | - | 0.00000399 | 0.000216 |
| protein targeting to membrane (GO:0006612) | 158 | + | 0.00000419 | 0.000226 |
| cell death (GO:0008219) | 1064 | - | 0.00000419 | 0.000225 |
| antigen processing and presentation of exogenous peptide antigen (GO:0002478) | 176 | - | 0.00000419 | 0.000225 |
| DNA replication-independent nucleosome assembly (GO:0006336) | 39 | - | 0.00000425 | 0.000227 |
| transcription by RNA polymerase II (GO:0006366) | 469 | - | 0.00000433 | 0.000231 |
| mitotic spindle assembly (GO:0090307) | 33 | - | 0.00000486 | 0.000257 |
| ATP-dependent chromatin remodeling (GO:0043044) | 71 | - | 0.00000546 | 0.000289 |
| DNA-dependent DNA replication (GO:0006261) | 117 | - | 0.0000055 | 0.00029 |
| DNA packaging (GO:0006323) | 171 | - | 0.00000555 | 0.000291 |
| nervous system process (GO:0050877) | 1305 | + | 0.00000555 | 0.00029 |
| intracellular transport (GO:0046907) | 1509 | - | 0.00000558 | 0.000291 |
| regulation of microtubule cytoskeleton organization (GO:0070507) | 189 | - | 0.0000056 | 0.000291 |
| phosphate-containing compound metabolic process (GO:0006796) | 2080 | - | 0.00000571 | 0.000296 |
| protein folding (GO:0006457) | 217 | - | 0.00000574 | 0.000296 |
| negative regulation of macromolecule biosynthetic process (GO:0010558) | 1433 | - | 0.00000579 | 0.000298 |
| positive regulation of nitrogen compound metabolic process (GO:0051173) | 3094 | - | 0.00000584 | 0.0003 |
| regulation of cell cycle G1/S phase transition (GO:1902806) | 165 | - | 0.00000634 | 0.000324 |
| negative regulation of mitotic nuclear division (GO:0045839) | 43 | - | 0.00000638 | 0.000325 |
| regulation of DNA replication (GO:0006275) | 108 | - | 0.00000643 | 0.000327 |
| regulation of mitochondrial outer membrane permeabilization involved in apoptotic signaling pathway (GO:1901028) | 46 | - | 0.00000667 | 0.000338 |
| regulation of cytokinesis (GO:0032465) | 88 | - | 0.00000691 | 0.000349 |
| positive regulation of cellular process (GO:0048522) | 5197 | - | 0.00000701 | 0.000353 |
| negative regulation of cellular macromolecule biosynthetic process (GO:2000113) | 1351 | - | 0.00000734 | 0.000368 |
| double-strand break repair (GO:0006302) | 167 | - | 0.00000743 | 0.000371 |
| mitotic spindle assembly checkpoint (GO:0007094) | 25 | - | 0.00000754 | 0.000376 |
| mitotic spindle checkpoint (GO:0071174) | 25 | - | 0.00000754 | 0.000374 |
| spindle assembly checkpoint (GO:0071173) | 25 | - | 0.00000754 | 0.000373 |
| spindle checkpoint (GO:0031577) | 25 | - | 0.00000754 | 0.000372 |
| protein modification by small protein conjugation (GO:0032446) | 753 | - | 0.00000763 | 0.000376 |
| intracellular protein transport (GO:0006886) | 970 | - | 0.00000804 | 0.000394 |
| mitotic DNA integrity checkpoint (GO:0044774) | 106 | - | 0.00000829 | 0.000406 |
| negative regulation of cellular biosynthetic process (GO:0031327) | 1485 | - | 0.00000866 | 0.000422 |
| positive regulation of cellular protein localization (GO:1903829) | 324 | - | 0.00000885 | 0.00043 |
| cellular protein modification process (GO:0006464) | 3023 | - | 0.00000909 | 0.000441 |
| protein modification process (GO:0036211) | 3023 | - | 0.00000909 | 0.000439 |
| programmed cell death (GO:0012501) | 1030 | - | 0.0000093 | 0.000448 |
| mitotic cytokinesis (GO:0000281) | 68 | - | 0.00000943 | 0.000453 |
| positive regulation of viral process (GO:0048524) | 104 | - | 0.00000948 | 0.000454 |
| organelle localization by membrane tethering (GO:0140056) | 168 | - | 0.00000963 | 0.00046 |
| phosphorus metabolic process (GO:0006793) | 2107 | - | 0.0000104 | 0.000496 |
| negative regulation of biosynthetic process (GO:0009890) | 1509 | - | 0.0000106 | 0.000503 |
| regulation of nucleocytoplasmic transport (GO:0046822) | 109 | - | 0.0000107 | 0.000505 |
| macromolecule catabolic process (GO:0009057) | 1003 | - | 0.0000109 | 0.000516 |
| G protein-coupled receptor signaling pathway (GO:0007186) | 1249 | + | 0.0000117 | 0.000549 |
| protein import (GO:0017038) | 133 | - | 0.0000123 | 0.000578 |
| spliceosomal complex assembly (GO:0000245) | 55 | - | 0.000013 | 0.000607 |
| protein localization (GO:0008104) | 2079 | - | 0.0000136 | 0.000632 |
| protein localization to chromosome, centromeric region (GO:0071459) | 17 | - | 0.0000138 | 0.000642 |
| positive regulation of mitochondrial outer membrane permeabilization involved in apoptotic signaling pathway 1(GO:1901030) | 37 | - | 0.000014 | 0.000648 |
| telomere organization (GO:0032200) | 102 | - | 0.000014 | 0.000647 |
| DNA geometric change (GO:0032392) | 93 | - | 0.0000141 | 0.000647 |
| symbiont process (GO:0044403) | 757 | - | 0.0000142 | 0.000651 |
| centrosome cycle (GO:0007098) | 83 | - | 0.0000142 | 0.00065 |
| regulation of G1/S transition of mitotic cell cycle (GO:2000045) | 151 | - | 0.0000142 | 0.000649 |
| regulation of ATP metabolic process (GO:1903578) | 107 | - | 0.0000144 | 0.000656 |
| regulation of DNA biosynthetic process (GO:2000278) | 109 | - | 0.0000147 | 0.000668 |
| negative regulation of cellular metabolic process (GO:0031324) | 2481 | - | 0.000015 | 0.000679 |
| antigen processing and presentation of peptide antigen (GO:0048002) | 185 | - | 0.0000159 | 0.000716 |
| non-canonical Wnt signaling pathway (GO:0035567) | 134 | - | 0.0000161 | 0.000723 |
| telomere maintenance (GO:0000723) | 99 | - | 0.0000162 | 0.000725 |
| protein-DNA complex subunit organization (GO:0071824) | 230 | - | 0.0000163 | 0.000731 |
| positive regulation of RNA metabolic process (GO:0051254) | 1683 | - | 0.000017 | 0.000759 |
| regulation of cyclin-dependent protein serine/threonine kinase activity (GO:0000079) | 98 | - | 0.0000171 | 0.000763 |
| tRNA processing (GO:0008033) | 128 | - | 0.0000173 | 0.000769 |
| alternative mRNA splicing, via spliceosome (GO:0000380) | 17 | - | 0.0000177 | 0.000784 |
| regulation of generation of precursor metabolites and energy (GO:0043467) | 143 | - | 0.0000182 | 0.000801 |
| protein modification by small protein removal (GO:0070646) | 285 | - | 0.0000195 | 0.000858 |
| regulation of signaling receptor activity (GO:0010469) | 600 | + | 0.0000196 | 0.00086 |
| RNA 3-end processing (GO:0031123) | 129 | - | 0.0000203 | 0.000887 |
| protein import into nucleus (GO:0006606) | 86 | - | 0.0000211 | 0.000921 |
| protein localization to organelle (GO:0033365) | 702 | - | 0.0000214 | 0.000933 |
| antigen processing and presentation (GO:0019882) | 212 | - | 0.000022 | 0.000953 |
| centromere complex assembly (GO:0034508) | 36 | - | 0.0000225 | 0.000976 |
| spindle localization (GO:0051653) | 42 | - | 0.0000229 | 0.000987 |
| signal transduction in response to DNA damage (GO:0042770) | 103 | - | 0.0000229 | 0.000988 |
| signal transduction by p53 class mediator (GO:0072331) | 121 | - | 0.0000249 | 0.00107 |
| regulation of establishment of planar polarity (GO:0090175) | 111 | - | 0.000025 | 0.00107 |
| negative regulation of nuclear division (GO:0051784) | 52 | - | 0.000026 | 0.00111 |
| regulation of cyclin-dependent protein kinase activity (GO:1904029) | 102 | - | 0.000027 | 0.00115 |
| macromolecule localization (GO:0033036) | 2397 | - | 0.0000274 | 0.00116 |
| SCF-dependent proteasomal ubiquitin-dependent protein catabolic process (GO:0031146) | 89 | - | 0.0000276 | 0.00117 |
| regulation of hematopoietic progenitor cell differentiation (GO:1901532) | 83 | - | 0.000028 | 0.00118 |
| homophilic cell adhesion via plasma membrane adhesion molecules (GO:0007156) | 167 | + | 0.0000285 | 0.0012 |
| regulation of small molecule metabolic process (GO:0062012) | 414 | - | 0.0000286 | 0.0012 |
| regulation of translational initiation (GO:0006446) | 77 | - | 0.0000295 | 0.00124 |
| regulation of transcription elongation from RNA polymerase II promoter (GO:0034243) | 28 | - | 0.0000303 | 0.00127 |
| cellular process (GO:0009987) | 13883 | - | 0.0000303 | 0.00126 |
| regulation of stem cell differentiation (GO:2000736) | 113 | - | 0.0000307 | 0.00128 |
| mitochondrion organization (GO:0007005) | 414 | - | 0.0000309 | 0.00128 |
| nuclear pore complex assembly (GO:0051292) | 9 | - | 0.0000317 | 0.00131 |
| positive regulation of gene expression (GO:0010628) | 1945 | - | 0.0000324 | 0.00134 |
| cell adhesion (GO:0007155) | 911 | + | 0.0000327 | 0.00135 |
| positive regulation of mitotic sister chromatid segregation (GO:0062033) | 17 | - | 0.0000329 | 0.00135 |
| cellular component disassembly (GO:0022411) | 386 | - | 0.0000351 | 0.00144 |
| meiotic cell cycle (GO:0051321) | 219 | - | 0.0000359 | 0.00147 |
| microtubule organizing center organization (GO:0031023) | 93 | - | 0.0000367 | 0.0015 |
| regulation of cellular protein metabolic process (GO:0032268) | 2550 | - | 0.0000372 | 0.00151 |
| regulation of attachment of spindle microtubules to kinetochore (GO:0051988) | 12 | - | 0.0000373 | 0.00151 |
| positive regulation of telomerase activity (GO:0051973) | 36 | - | 0.0000374 | 0.00151 |
| positive regulation of cell cycle phase transition (GO:1901989) | 94 | - | 0.0000378 | 0.00153 |
| tRNA aminoacylation for protein translation (GO:0006418) | 45 | - | 0.0000392 | 0.00157 |
| positive regulation of translation (GO:0045727) | 127 | - | 0.000045 | 0.0018 |
| viral process (GO:0016032) | 681 | - | 0.0000461 | 0.00184 |
| negative regulation of nitrogen compound metabolic process (GO:0051172) | 2281 | - | 0.0000466 | 0.00186 |
| mRNA stabilization (GO:0048255) | 43 | - | 0.0000468 | 0.00186 |
| protein deubiquitination (GO:0016579) | 268 | - | 0.0000471 | 0.00187 |
| establishment of mitotic spindle orientation (GO:0000132) | 23 | - | 0.0000471 | 0.00187 |
| female meiotic nuclear division (GO:0007143) | 29 | - | 0.000048 | 0.0019 |
| histone mRNA metabolic process (GO:0008334) | 25 | - | 0.0000496 | 0.00195 |
| biological adhesion (GO:0022610) | 917 | + | 0.0000501 | 0.00197 |
| cornification (GO:0070268) | 112 | - | 0.0000513 | 0.00201 |
| sensory perception (GO:0007600) | 893 | + | 0.0000544 | 0.00213 |
| regulation of DNA-templated transcription, elongation (GO:0032784) | 47 | - | 0.0000545 | 0.00213 |
| cell population proliferation (GO:0008283) | 659 | - | 0.0000549 | 0.00214 |
| positive regulation of viral genome replication (GO:0045070) | 35 | - | 0.000057 | 0.00221 |
| regulation of protein localization to nucleus (GO:1900180) | 115 | - | 0.0000571 | 0.00221 |
| positive regulation of DNA biosynthetic process (GO:2000573) | 68 | - | 0.0000573 | 0.00221 |
| signal transduction involved in DNA damage checkpoint (GO:0072422) | 71 | - | 0.0000595 | 0.0023 |
| signal transduction involved in DNA integrity checkpoint (GO:0072401) | 71 | - | 0.0000595 | 0.00229 |
| peptidyl-lysine modification (GO:0018205) | 296 | - | 0.0000598 | 0.00229 |
| chromosome condensation (GO:0030261) | 41 | - | 0.0000631 | 0.00242 |
| G1 DNA damage checkpoint (GO:0044783) | 67 | - | 0.0000645 | 0.00246 |
| antigen processing and presentation of exogenous peptide antigen via MHC class II (GO:0019886) | 98 | - | 0.0000648 | 0.00247 |
| DNA biosynthetic process (GO:0071897) | 100 | - | 0.0000665 | 0.00253 |
| regulation of ion transport (GO:0043269) | 677 | + | 0.0000682 | 0.00259 |
| positive regulation of biological process (GO:0048518) | 5901 | - | 0.0000691 | 0.00261 |
| regulation of microtubule-based process (GO:0032886) | 219 | - | 0.0000697 | 0.00263 |
| regulation of signal transduction by p53 class mediator (GO:1901796) | 179 | - | 0.0000698 | 0.00263 |
| positive regulation of mRNA 3-end processing (GO:0031442) | 11 | - | 0.0000702 | 0.00264 |
| signal transduction involved in cell cycle checkpoint (GO:0072395) | 72 | - | 0.000071 | 0.00266 |
| positive regulation of protein localization to nucleus (GO:1900182) | 77 | - | 0.0000731 | 0.00273 |
| interspecies interaction between organisms (GO:0044419) | 800 | - | 0.0000739 | 0.00275 |
| antigen processing and presentation of peptide antigen via MHC class II (GO:0002495) | 99 | - | 0.0000741 | 0.00275 |
| Fc-epsilon receptor signaling pathway (GO:0038095) | 112 | - | 0.0000767 | 0.00285 |
| negative regulation of G1/S transition of mitotic cell cycle (GO:2000134) | 105 | - | 0.0000834 | 0.00309 |
| negative regulation of nucleic acid-templated transcription (GO:1903507) | 1209 | - | 0.0000842 | 0.00311 |
| tRNA aminoacylation (GO:0043039) | 48 | - | 0.0000843 | 0.00311 |
| antigen processing and presentation of peptide or polysaccharide antigen via MHC class II (GO:0002504) | 100 | - | 0.0000844 | 0.0031 |
| positive regulation of cytosolic calcium ion concentration (GO:0007204) | 280 | + | 0.0000856 | 0.00314 |
| positive regulation of mitotic cell cycle phase transition (GO:1901992) | 79 | - | 0.0000858 | 0.00314 |
| tricarboxylic acid cycle (GO:0006099) | 35 | - | 0.0000867 | 0.00317 |
| phosphorylation (GO:0016310) | 1258 | - | 0.0000878 | 0.0032 |
| negative regulation of RNA biosynthetic process (GO:1902679) | 1211 | - | 0.0000878 | 0.00319 |
| secondary alcohol biosynthetic process (GO:1902653) | 42 | - | 0.0000938 | 0.0034 |
| cholesterol biosynthetic process (GO:0006695) | 42 | - | 0.0000938 | 0.00339 |
| regulation of cellular amino acid metabolic process (GO:0006521) | 63 | - | 0.000103 | 0.00373 |
| regulation of sister chromatid cohesion (GO:0007063) | 22 | - | 0.000104 | 0.00376 |
| amino acid activation (GO:0043038) | 49 | - | 0.000107 | 0.00383 |
| negative regulation of cell cycle G1/S phase transition (GO:1902807) | 107 | - | 0.00011 | 0.00393 |
| peptidyl-amino acid modification (GO:0018193) | 837 | - | 0.000111 | 0.00397 |
| transcription elongation from RNA polymerase II promoter (GO:0006368) | 73 | - | 0.000112 | 0.00399 |
| meiotic chromosome condensation (GO:0010032) | 6 | - | 0.000115 | 0.00409 |
| regulation of protein metabolic process (GO:0051246) | 2718 | - | 0.000118 | 0.0042 |
| ribosome localization (GO:0033750) | 13 | - | 0.00012 | 0.00427 |
| ribosomal subunit export from nucleus (GO:0000054) | 13 | - | 0.00012 | 0.00426 |
| mitotic G1/S transition checkpoint (GO:0044819) | 66 | - | 0.000124 | 0.00439 |
| mitotic G1 DNA damage checkpoint (GO:0031571) | 66 | - | 0.000124 | 0.00438 |
| sterol biosynthetic process (GO:0016126) | 47 | - | 0.000125 | 0.00437 |
| protein-DNA complex assembly (GO:0065004) | 193 | - | 0.000129 | 0.00453 |
| response to ionizing radiation (GO:0010212) | 143 | - | 0.000135 | 0.0047 |
| DNA duplex unwinding (GO:0032508) | 83 | - | 0.000135 | 0.0047 |
| negative regulation of mRNA processing (GO:0050686) | 30 | - | 0.000139 | 0.00483 |
| macromolecule methylation (GO:0043414) | 236 | - | 0.000139 | 0.00482 |
| regulation of binding (GO:0051098) | 371 | - | 0.000139 | 0.00481 |
| protein metabolic process (GO:0019538) | 4198 | - | 0.000142 | 0.0049 |
| establishment of spindle localization (GO:0051293) | 37 | - | 0.000142 | 0.0049 |
| viral transcription (GO:0019083) | 115 | + | 0.000155 | 0.00532 |
| nucleic acid phosphodiester bond hydrolysis (GO:0090305) | 276 | - | 0.000155 | 0.00533 |
| establishment of spindle orientation (GO:0051294) | 29 | - | 0.000161 | 0.00552 |
| pyruvate metabolic process (GO:0006090) | 68 | - | 0.000164 | 0.00562 |
| mitotic DNA damage checkpoint (GO:0044773) | 97 | - | 0.000165 | 0.00563 |
| regulation of mRNA 3-end processing (GO:0031440) | 26 | - | 0.000169 | 0.00575 |
| intracellular steroid hormone receptor signaling pathway (GO:0030518) | 69 | - | 0.000169 | 0.00573 |
| regulation of membrane permeability (GO:0090559) | 82 | - | 0.000171 | 0.00579 |
| tRNA modification (GO:0006400) | 86 | - | 0.000177 | 0.00599 |
| double-strand break repair via nonhomologous end joining (GO:0006303) | 52 | - | 0.000184 | 0.00622 |
| regulation of hematopoietic stem cell differentiation (GO:1902036) | 71 | - | 0.000185 | 0.00624 |
| signal transduction involved in mitotic G1 DNA damage checkpoint (GO:0072431) | 59 | - | 0.000191 | 0.00643 |
| signal transduction involved in mitotic cell cycle checkpoint (GO:0072413) | 59 | - | 0.000191 | 0.00642 |
| signal transduction involved in mitotic DNA integrity checkpoint (GO:1902403) | 59 | - | 0.000191 | 0.0064 |
| signal transduction involved in mitotic DNA damage checkpoint (GO:1902402) | 59 | - | 0.000191 | 0.00639 |
| intracellular signal transduction involved in G1 DNA damage checkpoint (GO:1902400) | 59 | - | 0.000191 | 0.00638 |
| cellular glucan metabolic process (GO:0006073) | 53 | - | 0.000198 | 0.0066 |
| glucan metabolic process (GO:0044042) | 53 | - | 0.000198 | 0.00659 |
| RNA polyadenylation (GO:0043631) | 31 | - | 0.000199 | 0.00659 |
| mitotic chromosome condensation (GO:0007076) | 17 | - | 0.000201 | 0.00664 |
| Golgi vesicle transport (GO:0048193) | 357 | - | 0.000204 | 0.00673 |
| cell-cell adhesion via plasma-membrane adhesion molecules (GO:0098742) | 257 | + | 0.000207 | 0.00682 |
| RNA-dependent DNA biosynthetic process (GO:0006278) | 26 | - | 0.000219 | 0.0072 |
| negative regulation of metabolic process (GO:0009892) | 2815 | - | 0.00022 | 0.00722 |
| chromatin remodeling at centromere (GO:0031055) | 32 | - | 0.000225 | 0.00736 |
| centrosome separation (GO:0051299) | 8 | - | 0.000225 | 0.00735 |
| positive regulation of transcription elongation from RNA polymerase II promoter (GO:0032968) | 17 | - | 0.000246 | 0.00802 |
| somatic recombination of immunoglobulin gene segments (GO:0016447) | 22 | - | 0.000252 | 0.00821 |
| DNA-templated transcription, elongation (GO:0006354) | 90 | - | 0.000253 | 0.00822 |
| regulation of telomerase activity (GO:0051972) | 50 | - | 0.000259 | 0.00837 |
| DNA damage response, signal transduction by p53 class mediator (GO:0030330) | 81 | - | 0.000259 | 0.00837 |
| regulation of cell cycle arrest (GO:0071156) | 111 | - | 0.000261 | 0.00843 |
| energy derivation by oxidation of organic compounds (GO:0015980) | 221 | - | 0.00027 | 0.0087 |
| centrosome duplication (GO:0051298) | 29 | - | 0.000275 | 0.00882 |
| viral life cycle (GO:0019058) | 196 | - | 0.000283 | 0.00907 |
| non-recombinational repair (GO:0000726) | 58 | - | 0.000284 | 0.0091 |
| ribosomal large subunit export from nucleus (GO:0000055) | 9 | - | 0.00029 | 0.00927 |
| attachment of spindle microtubules to kinetochore (GO:0008608) | 19 | - | 0.000293 | 0.00932 |
| organonitrogen compound metabolic process (GO:1901564) | 5170 | - | 0.000295 | 0.00937 |
| CENP-A containing nucleosome assembly (GO:0034080) | 30 | - | 0.000295 | 0.00936 |
| CENP-A containing chromatin organization (GO:0061641) | 30 | - | 0.000295 | 0.00934 |
| regulation of cellular localization (GO:0060341) | 889 | - | 0.000306 | 0.00966 |
| CRD-mediated mRNA stabilization (GO:0070934) | 5 | - | 0.000306 | 0.00965 |
| ATP metabolic process (GO:0046034) | 177 | - | 0.000306 | 0.00965 |
| regulation of transferase activity (GO:0051338) | 963 | - | 0.000308 | 0.00967 |
| regulation of centrosome cycle (GO:0046605) | 59 | - | 0.000315 | 0.00989 |
| regulation of spindle organization (GO:0090224) | 43 | - | 0.000316 | 0.00988 |
| establishment of protein localization (GO:0045184) | 1547 | - | 0.000318 | 0.00994 |
| purine ribonucleoside metabolic process (GO:0046128) | 58 | - | 0.000319 | 0.00993 |
| interleukin-1-mediated signaling pathway (GO:0070498) | 96 | - | 0.000319 | 0.00994 |
| positive regulation of RNA biosynthetic process (GO:1902680) | 1598 | - | 0.000338 | 0.0105 |
| response to UV (GO:0009411) | 141 | - | 0.000341 | 0.0106 |
| chromatin assembly or disassembly (GO:0006333) | 150 | - | 0.000354 | 0.0109 |
| gene silencing (GO:0016458) | 150 | - | 0.000355 | 0.011 |
| regulation of double-strand break repair (GO:2000779) | 71 | - | 0.000359 | 0.0111 |
| regulation of mitotic spindle organization (GO:0060236) | 39 | - | 0.00036 | 0.0111 |
| regulation of carbohydrate catabolic process (GO:0043470) | 81 | - | 0.00037 | 0.0114 |
| negative regulation of transcription, DNA-templated (GO:0045892) | 1156 | - | 0.000374 | 0.0115 |
| positive regulation of nucleic acid-templated transcription (GO:1903508) | 1597 | - | 0.000376 | 0.0115 |
| RNA localization to nucleus (GO:0090685) | 5 | - | 0.000379 | 0.0116 |
| RNA localization to Cajal body (GO:0090670) | 5 | - | 0.000379 | 0.0115 |
| scaRNA localization to Cajal body (GO:0090666) | 5 | - | 0.000379 | 0.0115 |
| DNA damage response, signal transduction by p53 class mediator resulting in cell cycle arrest (GO:0006977) | 58 | - | 0.000381 | 0.0116 |
| ion transport (GO:0006811) | 1303 | + | 0.000385 | 0.0117 |
| protein transport (GO:0015031) | 1467 | - | 0.0004 | 0.0121 |
| ion transmembrane transport (GO:0034220) | 877 | + | 0.000405 | 0.0122 |
| glycogen metabolic process (GO:0005977) | 52 | - | 0.000406 | 0.0122 |
| protein localization to kinetochore (GO:0034501) | 12 | - | 0.00042 | 0.0126 |
| positive regulation of viral transcription (GO:0050434) | 41 | - | 0.000424 | 0.0127 |
| regulation of glycolytic process (GO:0006110) | 73 | - | 0.000437 | 0.0131 |
| meiotic cell cycle process (GO:1903046) | 161 | - | 0.000451 | 0.0135 |
| regulation of cytosolic calcium ion concentration (GO:0051480) | 323 | + | 0.000453 | 0.0135 |
| inorganic ion transmembrane transport (GO:0098660) | 625 | + | 0.00046 | 0.0137 |
| negative regulation of mRNA splicing, via spliceosome (GO:0048025) | 21 | - | 0.000469 | 0.0139 |
| cell cycle DNA replication (GO:0044786) | 41 | - | 0.000472 | 0.014 |
| DNA-templated transcription, termination (GO:0006353) | 73 | - | 0.00052 | 0.0154 |
| purine nucleoside metabolic process (GO:0042278) | 61 | - | 0.000529 | 0.0156 |
| ncRNA transcription (GO:0098781) | 88 | - | 0.000531 | 0.0156 |
| negative regulation of macromolecule metabolic process (GO:0010605) | 2571 | - | 0.000545 | 0.016 |
| negative regulation of DNA metabolic process (GO:0051053) | 117 | - | 0.000548 | 0.0161 |
| positive regulation of multi-organism process (GO:0043902) | 185 | - | 0.000561 | 0.0165 |
| regulation of nucleobase-containing compound transport (GO:0032239) | 16 | - | 0.000573 | 0.0168 |
| positive regulation of cytokinesis (GO:0032467) | 39 | - | 0.000583 | 0.017 |
| positive regulation of response to DNA damage stimulus (GO:2001022) | 96 | - | 0.00059 | 0.0172 |
| amide biosynthetic process (GO:0043604) | 519 | - | 0.00061 | 0.0177 |
| covalent chromatin modification (GO:0016569) | 361 | - | 0.000611 | 0.0177 |
| positive regulation of cellular amide metabolic process (GO:0034250) | 147 | - | 0.000638 | 0.0185 |
| generation of precursor metabolites and energy (GO:0006091) | 395 | - | 0.000643 | 0.0186 |
| metaphase/anaphase transition of mitotic cell cycle (GO:0007091) | 5 | - | 0.000645 | 0.0186 |
| metaphase/anaphase transition of cell cycle (GO:0044784) | 5 | - | 0.000645 | 0.0186 |
| regulation of DNA-templated transcription, initiation (GO:2000142) | 38 | - | 0.00065 | 0.0187 |
| DNA recombination (GO:0006310) | 202 | - | 0.000651 | 0.0187 |
| regulation of mitochondrial membrane permeability (GO:0046902) | 72 | - | 0.000665 | 0.0191 |
| nucleotide metabolic process (GO:0009117) | 422 | - | 0.000667 | 0.0191 |
| NIK/NF-kappaB signaling (GO:0038061) | 81 | - | 0.000675 | 0.0193 |
| sister chromatid cohesion (GO:0007062) | 47 | - | 0.000682 | 0.0195 |
| somatic diversification of immunoglobulins (GO:0016445) | 32 | - | 0.000688 | 0.0196 |
| regulation of DNA-dependent DNA replication (GO:0090329) | 53 | - | 0.000689 | 0.0196 |
| nucleosome organization (GO:0034728) | 149 | - | 0.00071 | 0.0201 |
| cation transport (GO:0006812) | 809 | + | 0.000717 | 0.0203 |
| positive regulation of chromatin silencing (GO:0031937) | 14 | - | 0.000737 | 0.0208 |
| androgen receptor signaling pathway (GO:0030521) | 35 | - | 0.000739 | 0.0209 |
| amide transport (GO:0042886) | 1522 | - | 0.000763 | 0.0215 |
| positive regulation of transcription, DNA-templated (GO:0045893) | 1513 | - | 0.000766 | 0.0215 |
| viral genome replication (GO:0019079) | 27 | - | 0.000783 | 0.022 |
| positive regulation of DNA-templated transcription, elongation (GO:0032786) | 29 | - | 0.000795 | 0.0223 |
| regulation of RNA binding (GO:1905214) | 12 | - | 0.000798 | 0.0223 |
| regulation of RNA export from nucleus (GO:0046831) | 14 | - | 0.0008 | 0.0223 |
| locomotory behavior (GO:0007626) | 200 | + | 0.00082 | 0.0229 |
| telomere maintenance via telomerase (GO:0007004) | 23 | - | 0.000827 | 0.023 |
| regulation of protein localization to cell cortex (GO:1904776) | 7 | - | 0.000828 | 0.023 |
| mRNA polyadenylation (GO:0006378) | 29 | - | 0.000835 | 0.0231 |
| RNA modification (GO:0009451) | 161 | - | 0.000838 | 0.0232 |
| somatic diversification of immunoglobulins involved in immune response (GO:0002208) | 18 | - | 0.000842 | 0.0233 |
| somatic recombination of immunoglobulin genes involved in immune response (GO:0002204) | 18 | - | 0.000842 | 0.0232 |
| isotype switching (GO:0045190) | 18 | - | 0.000842 | 0.0232 |
| peptide transport (GO:0015833) | 1491 | - | 0.000862 | 0.0237 |
| nuclear DNA replication (GO:0033260) | 40 | - | 0.000863 | 0.0237 |
| nucleoside phosphate metabolic process (GO:0006753) | 429 | - | 0.000872 | 0.0239 |
| defense response (GO:0006952) | 1210 | + | 0.000919 | 0.0251 |
| negative regulation of translation (GO:0017148) | 131 | - | 0.000929 | 0.0254 |
| protein K48-linked ubiquitination (GO:0070936) | 51 | - | 0.000937 | 0.0255 |
| regulation of chromatin organization (GO:1902275) | 186 | - | 0.000946 | 0.0257 |
| regulation of transcription by RNA polymerase II (GO:0006357) | 2653 | - | 0.000971 | 0.0264 |
| histone exchange (GO:0043486) | 45 | - | 0.000979 | 0.0265 |
| mitotic sister chromatid cohesion (GO:0007064) | 14 | - | 0.00098 | 0.0265 |
| negative regulation of gene expression (GO:0010629) | 1668 | - | 0.000982 | 0.0265 |
| regulation of leukocyte proliferation (GO:0070663) | 222 | + | 0.000989 | 0.0267 |
| regulation of miRNA metabolic process (GO:2000628) | 10 | - | 0.000994 | 0.0268 |
| histone modification (GO:0016570) | 351 | - | 0.001 | 0.027 |
| negative regulation of response to DNA damage stimulus (GO:2001021) | 77 | - | 0.00103 | 0.0275 |
| regulation of organelle assembly (GO:1902115) | 189 | - | 0.00103 | 0.0275 |
| positive regulation of transcription by RNA polymerase I (GO:0045943) | 22 | - | 0.00103 | 0.0276 |
| steroid hormone mediated signaling pathway (GO:0043401) | 120 | - | 0.00104 | 0.0277 |
| regulation of response to external stimulus (GO:0032101) | 753 | + | 0.00104 | 0.0277 |
| stimulatory C-type lectin receptor signaling pathway (GO:0002223) | 112 | - | 0.00105 | 0.0278 |
| female meiosis I (GO:0007144) | 8 | - | 0.00106 | 0.028 |
| negative regulation of gene expression, epigenetic (GO:0045814) | 80 | - | 0.00108 | 0.0287 |
| regulation of morphogenesis of an epithelium (GO:1905330) | 179 | - | 0.00109 | 0.0287 |
| oxidation-reduction process (GO:0055114) | 922 | - | 0.00109 | 0.0289 |
| RNA phosphodiester bond hydrolysis (GO:0090501) | 145 | - | 0.00115 | 0.0304 |
| guanosine-containing compound metabolic process (GO:1901068) | 38 | - | 0.00115 | 0.0304 |
| myofibril assembly (GO:0030239) | 63 | + | 0.00117 | 0.0307 |
| establishment of cell polarity (GO:0030010) | 110 | - | 0.00117 | 0.0307 |
| regulation of telomere capping (GO:1904353) | 26 | - | 0.00119 | 0.0311 |
| cation transmembrane transport (GO:0098655) | 594 | + | 0.00119 | 0.0312 |
| regulation of cell aging (GO:0090342) | 46 | - | 0.00121 | 0.0317 |
| sequestering of zinc ion (GO:0032119) | 4 | - | 0.00122 | 0.0318 |
| positive regulation of DNA replication (GO:0045740) | 38 | - | 0.00124 | 0.0322 |
| regulation of interferon-gamma production (GO:0032649) | 100 | + | 0.00124 | 0.0322 |
| actomyosin contractile ring organization (GO:0044837) | 6 | - | 0.00127 | 0.0329 |
| collagen-activated tyrosine kinase receptor signaling pathway (GO:0038063) | 10 | + | 0.00127 | 0.0328 |
| positive regulation of lymphocyte proliferation (GO:0050671) | 134 | + | 0.00127 | 0.0328 |
| positive regulation of cellular catabolic process (GO:0031331) | 354 | - | 0.00127 | 0.0328 |
| positive regulation of mononuclear cell proliferation (GO:0032946) | 135 | + | 0.00128 | 0.0329 |
| inorganic cation transmembrane transport (GO:0098662) | 541 | + | 0.00129 | 0.0332 |
| positive regulation of DNA repair (GO:0045739) | 63 | - | 0.00129 | 0.0332 |
| autophagy of peroxisome (GO:0030242) | 5 | - | 0.0013 | 0.0333 |
| regulation of cell activation (GO:0050865) | 536 | + | 0.0013 | 0.0333 |
| tRNA methylation (GO:0030488) | 39 | - | 0.00131 | 0.0335 |
| positive regulation of leukocyte proliferation (GO:0070665) | 139 | + | 0.00131 | 0.0336 |
| apoptotic process (GO:0006915) | 900 | - | 0.00132 | 0.0338 |
| response to X-ray (GO:0010165) | 29 | - | 0.00132 | 0.0337 |
| nucleoside triphosphate metabolic process (GO:0009141) | 79 | - | 0.00133 | 0.0339 |
| negative regulation of cellular process (GO:0048523) | 4583 | - | 0.00134 | 0.0341 |
| RNA 5-end processing (GO:0000966) | 21 | - | 0.00135 | 0.0342 |
| innate immune response activating cell surface receptor signaling pathway (GO:0002220) | 115 | - | 0.00136 | 0.0345 |
| histone H2B ubiquitination (GO:0033523) | 10 | - | 0.00139 | 0.0352 |
| N-terminal peptidyl-methionine acetylation (GO:0017196) | 7 | - | 0.00144 | 0.0364 |
| negative regulation of kinase activity (GO:0033673) | 238 | - | 0.00145 | 0.0366 |
| positive regulation of histone ubiquitination (GO:0033184) | 5 | - | 0.00147 | 0.0368 |
| regulation of double-strand break repair via homologous recombination (GO:0010569) | 43 | - | 0.00147 | 0.0369 |
| NADH regeneration (GO:0006735) | 25 | - | 0.00147 | 0.0369 |
| glucose catabolic process to pyruvate (GO:0061718) | 25 | - | 0.00147 | 0.0369 |
| canonical glycolysis (GO:0061621) | 25 | - | 0.00147 | 0.0368 |
| somatic diversification of immune receptors via germline recombination within a single locus (GO:0002562) | 31 | - | 0.00149 | 0.037 |
| somatic cell DNA recombination (GO:0016444) | 31 | - | 0.00149 | 0.037 |
| positive regulation of cytokine production (GO:0001819) | 436 | + | 0.00149 | 0.0369 |
| regulation of ion transmembrane transport (GO:0034765) | 465 | + | 0.0015 | 0.0371 |
| rRNA-containing ribonucleoprotein complex export from nucleus (GO:0071428) | 15 | - | 0.00151 | 0.0373 |
| glucose catabolic process (GO:0006007) | 29 | - | 0.00152 | 0.0375 |
| calcium ion homeostasis (GO:0055074) | 440 | + | 0.00153 | 0.0379 |
| regulation of cytokine production (GO:0001817) | 679 | + | 0.00154 | 0.038 |
| response to stress (GO:0006950) | 3330 | - | 0.00154 | 0.038 |
| attachment of mitotic spindle microtubules to kinetochore (GO:0051315) | 11 | - | 0.00159 | 0.0392 |
| positive regulation of RNA splicing (GO:0033120) | 35 | - | 0.0016 | 0.0392 |
| regulation of cellular process (GO:0050794) | 10501 | - | 0.00161 | 0.0395 |
| positive regulation of nucleocytoplasmic transport (GO:0046824) | 64 | - | 0.00162 | 0.0396 |
| substantia nigra development (GO:0021762) | 45 | - | 0.00163 | 0.0397 |
| cellular calcium ion homeostasis (GO:0006874) | 426 | + | 0.00163 | 0.0399 |
| gene silencing by RNA (GO:0031047) | 86 | - | 0.00167 | 0.0406 |
| purine-containing compound metabolic process (GO:0072521) | 348 | - | 0.00169 | 0.041 |
| positive regulation of mammary gland epithelial cell proliferation (GO:0033601) | 10 | - | 0.00176 | 0.0427 |
| regulation of transcription by RNA polymerase I (GO:0006356) | 32 | - | 0.00177 | 0.0429 |
| interleukin-12-mediated signaling pathway (GO:0035722) | 46 | - | 0.0018 | 0.0436 |
| inflammatory response (GO:0006954) | 483 | + | 0.0018 | 0.0435 |
| proteasomal ubiquitin-independent protein catabolic process (GO:0010499) | 23 | - | 0.00183 | 0.0442 |
| organonitrogen compound catabolic process (GO:1901565) | 1012 | - | 0.00185 | 0.0447 |
| protein dephosphorylation (GO:0006470) | 206 | - | 0.00186 | 0.0446 |
| chromatin assembly (GO:0031497) | 129 | - | 0.00187 | 0.0449 |
| sequestering of metal ion (GO:0051238) | 11 | - | 0.00187 | 0.0449 |
| methylation (GO:0032259) | 298 | - | 0.00189 | 0.0452 |
| regulation of inflammatory response (GO:0050727) | 325 | + | 0.00189 | 0.0453 |
| protein localization to cytoskeleton (GO:0044380) | 39 | - | 0.00189 | 0.0452 |
| regulation of viral process (GO:0050792) | 200 | - | 0.0019 | 0.0452 |
| ribose phosphate metabolic process (GO:0019693) | 316 | - | 0.0019 | 0.0453 |
| female meiosis chromosome segregation (GO:0016321) | 6 | - | 0.00191 | 0.0454 |
| nucleobase-containing small molecule metabolic process (GO:0055086) | 511 | - | 0.00191 | 0.0454 |
| regulation of metal ion transport (GO:0010959) | 377 | + | 0.00195 | 0.0461 |
| regulation of mononuclear cell proliferation (GO:0032944) | 213 | + | 0.00195 | 0.0462 |
| negative regulation of phosphorus metabolic process (GO:0010563) | 544 | - | 0.00196 | 0.0462 |
| regulation of lymphocyte proliferation (GO:0050670) | 212 | + | 0.00196 | 0.0462 |
| GTP metabolic process (GO:0046039) | 24 | - | 0.00196 | 0.0462 |
| glycolytic process through glucose-6-phosphate (GO:0061620) | 26 | - | 0.00197 | 0.0462 |
| glycolytic process through fructose-6-phosphate (GO:0061615) | 26 | - | 0.00197 | 0.0461 |
| regulation of alternative mRNA splicing, via spliceosome (GO:0000381) | 64 | - | 0.00199 | 0.0466 |
| nitrogen compound transport (GO:0071705) | 1774 | - | 0.00203 | 0.0476 |
| somatic diversification of immune receptors (GO:0002200) | 41 | - | 0.00207 | 0.0483 |
| embryonic cleavage (GO:0040016) | 6 | - | 0.00209 | 0.0488 |
| nucleotide-excision repair (GO:0006289) | 108 | - | 0.0021 | 0.0489 |
| pentose biosynthetic process (GO:0019322) | 4 | - | 0.00212 | 0.0493 |
| glycosyl compound metabolic process (GO:1901657) | 130 | - | 0.00213 | 0.0496 |
| antigen processing and presentation of peptide antigen via MHC class I (GO:0002474) | 94 | - | 0.00215 | 0.05 |
